# Supplementary material for: Perpetrators of gender-based workplace violence amongst nurses and physicians–A scoping review of the literature
Source: PLOS Glob Public Health. 2024 Sep 6;4(9):e0003646. doi: 10.1371/journal.pgph.0003646 (PMC11379169; doi:10.1371/journal.pgph.0003646)
Supplement: S1 Text — (PDF) [file pgph.0003646.s002.pdf]

Definitions of forms of workplace violence against healthcare providers

Physical violence (assault, attack)	Attempt at physical injury on a person leading to physical harm. This may include hitting, punching, kicking, stabbing, shooting, biting, sexual assault, and rape.
Threat	Threats of violence or use of force resulting in fear of physical, sexual, or psychological harm or other negative consequences.
Abuse	Behaviour that is outside of reasonable conduct and involves the misuse of strength or power. This may include harassment, bullying/mobbing.
Harassment	Unwanted conduct – including verbal, non-verbal, psychological, or physical – is directed at an individual based on their age, disability, race, sex, sexual orientation, race, language, religion, or other status that negatively affects the dignity of the targeted individual at work. This includes sexual harassment.
Sexual harassment	Unwanted conduct of a sexual nature that is perceived by the victim as placing a condition on her/his employment, or that might be perceived by the victim as an offence, humiliation, or a threat to his/her well-being.
Bullying/mobbing	A form of harassment that consists in persecutory behaviour that targets an individual or group through malicious attempts to humiliate or undermine them, including unjustified, constant negative criticism, isolating a person from the rest of the group, gossiping, or spreading false information about the person.

Di Martino, V. (2003). Relationship of Work Stress and Workplace Violence in the Health Sector. ILO, ICN, WHO, PSI Joint Programme on Workplace Violence in the Health Sector. Geneva
