# Supplementary material for: Perpetrators of gender-based workplace violence amongst nurses and physicians–A scoping review of the literature
Source: PLOS Glob Public Health. 2024 Sep 6;4(9):e0003646. doi: 10.1371/journal.pgph.0003646 (PMC11379169; doi:10.1371/journal.pgph.0003646)
Supplement: S2 Text — (PDF) [file pgph.0003646.s003.pdf]

Workplace Violence Amongst the Global Health Workforce Based on Victims' Gender—A Scoping Review of the Literature

In order to contribute to achieving universal health coverage by 2030, the World Health Organization (WHO) and the Global Health Workforce Alliance proposed the global strategy on human resources for health, which was adopted in 2016. A foundational principle of the strategy is to "uphold the personal, employment and professional rights of all health workers, including safe and decent working environments and freedom from all kinds of discrimination, coercion, and violence" (WHO, 2016, p.8). However, globally, workplace violence (WPV) is a significant issue that impacts individuals, healthcare organizations, and society, particularly women in the health workforce (George et al., 2020). Workplace violence drew attention in the healthcare sector during the 1990s and early 2000 because of increased violent incidences. During that time, one-third of workplace violence reported was from the healthcare sector. In 2000, The International Labour Office (ILO), the International Council of Nurses (ICN), the WHO, and Public Services International (PSI) launched a joint program aiming to develop policies and guidelines for the prevention and elimination of violence in the healthcare sector (ILO, ICN, WHO & PSI, 2002).

The phenomenon of violence and harassment comprises "a range of unacceptable behaviors, practices or threats thereof, whether a single occurrence or repeated, that aim at, result in, or are likely to result in physical, psychological, sexual or economic harm" (ILO, 2020, p.8). The general definition of violence was adopted by the framework guidelines on addressing WPV by ILO, ICN, WHO and PSI (2002) is “incidents where staff are abused, threatened or assaulted in circumstances related to their work, including commuting to and from work, involving an explicit or implicit challenge to their safety, well-being or health” (p.3). Since then, this definition has been followed by different sources (George et al., 2020; Liu et al., 2019), which is comprehensive and applicable to all sectors, including healthcare

and this review. The specified incidences pose challenges to individuals (Tonso et al., 2016; Anand et al., 2016; Hassankhani et al., 2017; Han et al., 2017), the healthcare organization (Han et al., 2017; Lanctôt & Guay, 2014; George et al., 2020) and the society at large (George et al., 2020). Since the implementation of guidelines and policies by ILO, ICN, WHO, and PSI (2002) to address WPV in the health sector, several studies conducted for different aspects of WPV, including the prevalence, risk factors (Liu et al., 2019), and interventions to deescalate or eliminate the WPV, in different clinical settings, geographic locations and countries (Asi & Williams, 2020; Han et al., 2017; Newman et al., 2011) for different populations in the health workforce. The current scoping review will focus on understanding gender-based WPV and related aspects in the global health workforce, including midwives, nurses, and physicians. Because gender, a social determinant, differently influences males and females in the health workforce. However, women's experience of discrimination is higher than men's for different kinds and contexts (WHO, 2019).

Influence of Gender on Workplace Violence

As part of the joint program of ILO, ICN, WHO, and PSI, several case studies were conducted in six countries, including developed and developing countries. More than 50% of the participants in six case studies experienced physical or psychological violence once in the previous year; violence affected all healthcare providers regardless of their profession and gender (di Martino, 2003). However, it is particularly harmful to women due to their preponderance in the health workforce globally and the gender power relations, which are critical to ensure the safety and well-being of the health care providers and provide quality care (George et al., 2020), WHO, 2019). A systematic review (Lanctôt & Guay, 2014) for consequences of the exposure to WPV in the healthcare setting from 68 studies reported psychological and emotional effects such as post-traumatic stress, depression, anger, and fear impacting the level of functioning, leads to increased sick leaves, poor job satisfaction,

burnout, and higher attrition rates, particularly for women (Lanctôt & Guay, 2014). Failure to address gender-based WPV, women may not pursue a career in the health professions (George et al., 2020), which can adversely affect the universal health coverage leading to poor health outcomes for women.

Despite several efforts to overcome it, the prevalence of WPV is alarmingly high in different countries and professional groups. For instance, nurses (91.4%) experienced WPV in Lebanon (Darawad et al., 2015), China (71.9%) (Jiao et al., 2015), and in the USA (90%) (Ceravolo et al., 2012), physicians (41%) in India (Anand et al., 2016), and nurses and physicians in Barbados (Abed et al., 2016). Abed et al. (2016) found gender as a significant predictor (OR = 9; 95% CI 2–45) for WPV in primary care clinics in Barbados; female nurses and physicians were 11 times more likely to experience verbal abuse and nine times more likely to experience any type of violence than males. The study also reported nurses being twice at risk for experiencing verbal abuse compared to physicians (Abed et al., 2016). Another study from Brazil in the emergency care unit reported that female nurses were 5.8 times more likely to experience verbal violence than males (Ceballos et al., 2020). A systematic review of 253 studies and meta-analysis for the prevalence of the WPV by patients and visitors against healthcare workers reported 61.9%; non-physical violence contributed to 42.5%, and physical violence 24.4% in the last 12 months. The proportion of WPV exposure differed across countries, study location, practice settings, work schedules, and occupations. Of 253, only 68 studies reported sex-segregated results for men and women; other studies did not differentiate findings for sex/gender aspects. The meta-analysis did not determine the significant differences in the prevalence of any form of WPV according to sex (Liu et al., 2019), which attributes to the sample of studies included in the review, and only 27% of studies included in the review presented the sex-segregated findings. Instead, a narrative review reported female health workforce is affected more often, including in the

United States, the Republic of Korea, Rwanda, Nepal, Bangladesh, Malawi and Ghana, Kenya, the Democratic Republic of Congo, Pakistan, Libya, and Mexico (George et al., 2020). However, a couple of sources presented that males are at higher risk for physical violence than females, who are at higher risk for non-physical WPV (Guay et al., 2014; Liu et al., 2019). These research evidence present a need for a comprehensive review for the influence of gender on WPV and way to address it.

In the earlier case studies, presented measures for WPV focused on improving the physical environment and security rather than strategic and organizational aspects. Based on evidence, the case studies by di Martino (di Martino, 2003) recommended an integrated interventions approach at the macro, meso, and micro levels to address violence prevention. Because violence is an issue of societal structures rooted in socio-economic, cultural, and institutional factors, addressing it requires a systematic approach, which is integrated, participatory, culture/gender-sensitive, and non-discriminatory (International Labour Organization et al., 2002). To date, the focus of interventions is mostly on assessing the effectiveness of training interventions to prevent and manage WPV in healthcare settings. Example of such an intervention in a randomized controlled trial on the incidence of patient-to-worker (Type II) violence and related injuries in 41 units across seven hospitals in Midwest US. The intervention effectively decreased risks of patient-to-worker violence and related injury on the intervention units compared to controlled units. In the same study, though the follow-up survey response was low for the control group, 100% of the responded intervention units had implemented violence prevention strategies, compared to 80% of the responded control units (Arnetz et al., 20 17).

Another quality improvement project in the north-eastern US reveals nurses reporting a decrease in the experience of verbal abuse by 14% after attending a series of workshops and feeling determined to solve problems after experiencing lateral violence, which resulted in

lower turnover and vacancy rates (Ceravolo et al., 2012). An experimental study in Karachi, Pakistan, reported a training intervention's effectiveness in building confidence among healthcare professionals to cope with patients' aggression to prevent, deescalate, and manage WPV in healthcare settings. However, there were no significant differences between intervention and control groups for the frequency of violence (Baig et al., 2018). Although several studies highlighted that women's and men's experiences and responses differed about WPV, these interventional studies did not present gender aspects (Arnetz et al., 2017; Baig et al., 2018; Ceravolo et al., 2012). A recent paper by Asha George and colleagues highlights that WPV against female healthcare providers is the tip of the iceberg; the gender power relations and the intersection of other factors, including age, ethnicity, and income, hidden at the base of the iceberg are responsible for different forms and sources of violence experienced by the female healthcare providers (George et al., 2020). The women's concentration in the lowered tiers in the health professions places them in a vulnerable position for WPV, expressed by several sources over decades (George et al., 2020; International Labour Organization et al., 2002). Therefore, the structural power relations need to be addressed to prevent and eliminate gender-based violence amongst the health workforce. These structural power relations may also affect the reporting of such incidences that could lead to erroneous prevalence rates.

An essential aspect of escalating WPV healthcare providers is underreporting (Darawad et al., 2015; Han et al., 2017; Liu et al., 2019), which is considered as part of the job mostly by nurses, and participants did not feel that reporting is supported at the workplace (Child & Mentes, 2010; Han et al., 2017). In these studies, most nurses were female who also felt reporting was not supported by the organizations (Han et al., 2017), and men being at the top of the hierarchy in the society could explain the thinking and incidents of violence (George et al., 2020; International Labour Organization et al., 2002).

Summary of the literature and Rationale

Since the launching of the joint program by the ILO, the ICN, WHO, and PSI in 2000, aimed to prevent and eliminate WPV from the healthcare sector in 2002, several sources, including systematic reviews, have acknowledged increased WPV across the world among various categories of the healthcare professionals. WPV is also varyingly high in different locations and healthcare settings for physical and non-physical violence and its consequences at all levels. Some studies indicate WPV is more prevalent among the female health workforce, while others claim it is prevalent among males. Hence, it is crucial to explore the factors and forces behind these contradictory findings in a diverse context to provide a clear picture of the gender-based WPV phenomenon. Various studies focused on interventions preventing or managing WPV with positive outcomes; however, they are not gendered. On balance, the influence of gender on experiences of WPV has not been widely studied. Therefore, we propose conducting a scoping review of the literature to map existing evidence on aspects of gender-based violence, including the prevalence, risk factors and perpetrators, and preventive interventions that could lead to effective recommendations.

Aim of the scoping review

We aim to map the available evidence on the prevalence of WPV based on gender, including physical and non-physical violence among nurses, midwives, and physicians across different contexts. In addition, it will explore the reasons behind gender-based WPV amongst the identified healthcare professionals leading to providing gender-based recommendations to guide future research and practice.

Review Objectives

The specific objectives will be:

1. Map the most frequent forms and prevalence of Gender-based WPV for midwives, nurses, and physicians in different contexts and clinical settings.

2. Identify the gendered dimensions of the health workforce that underpin violence against female health workers.
3. Determine successful and gender-sensitive strategies/interventions in different contexts for policy recommendations.
4. Identify gaps in the state of knowledge to recommend empirical research studies.

Review Questions

What are the differences in exposure to gender-based violence at the workplace among the health workforce globally? Are men or women at greater risk in different professions, including physicians, nurses, and midwives in different contexts and healthcare facilities? What interventions are implemented in different contexts, professions, geographic locations, and clinical settings? Are the implemented interventions successful and gender-sensitive?

Methods

Study design

The scoping literature review design will be used to address the questions mentioned above, adapting the Joanna Briggs Institute's (JBI) revised guidelines. Because scoping reviewed is an appropriate method to explore the extent of the literature, map and summarize the evidence, and identify and analyze the knowledge gap to inform future research (Peters et al., 2020). This framework consists of eight steps, originated from the seminal framework (six steps) of Arksey and O'Malley's scoping review, which was advanced by Levac and collogued (Arksey & O'Malley, 2005; Levac et al., 2010). In the revised guidelines, JBI aligned these eight steps with the Preferred Reporting Items for Systematic and Meta-Analyses extension for Scoping Reviews (PRISMA-ScR) (Tricco et al., 2018) will be used to report the conduct of the scoping review that will provide rigor, transparency, and trustworthiness (Peters et al., 2020).

The first step of the scoping review framework is to align research objectives and question/s described in the earlier paragraphs in this protocol.

Inclusion Criteria for Studies

1. Participants, including midwives, nurses, and physicians, experienced workplace violence during their careers.
2. Participants experienced violence (any kind) in the workplace during their careers.
3. Provided sex segregated data or presented findings of gender as a construct for any kind of violence among midwives, nurses, and physicians, including students in the global context.
4. Published in English and after 2010 except for seminal works.

Exclusion criteria

1. Studies that did not provide sex-segregated data or and did not report findings of violence based on gender
2. Exclude systematic/ scoping reviews, concept or theoretical paper, and thesis.

Note: Appendix "A" specifies search terms rated to the population, concepts, and context for the scoping review.

Search Strategy

The research team will develop a comprehensive search strategy in consultation and assistance from the health sciences librarian. The search will focus on the systematic search of published literature in the databases, including Ovid MEDLINE: Epub Ahead of Print, In-Process and Other Non-Indexed Citations, which will be translated in CINAHL Plus, APA PsycINFO, Web of Sciences, and Gender Studies Databases, including Applied Social Sciences Index & Abstracts (ASSIA) and Sociological Abstracts. The search terms in appendix ‘A’ will be used keeping in mind the PCC pneumonic for searching for the scoping review, which denotes population, concept, and context in appropriate combination (Peters et

al., 2020). These terms are identified from the preliminary search of the literature on different aspects of WPV in Google Scholar. The final search results will be exported to EndNote, a citation manager, for de-duplication of the sources from multiple databases. After de-duplication, these sources will be imported into the Covidence online software program to streamline the screening process by two independent reviewers.

Evidence Screening and Selection

The identified sources will be selected based on the set inclusion criteria. Two independent reviewers will screen the title and abstracts. The discrepancies will be resolved with discussion and consensus and by reviewing the complete source, followed by a full-text review for selected sources against the set inclusion criteria by two reviewers and abstraction of the information independently. The selection process will be described and presented in PRISMA diagrammatic format.

Data Extraction

Data will be extracted in a Microsoft Excel Sheet from all the selected sources. A pilot test will be conducted for five sources to chart the information regarding author/s and year, title, source, country, objective/purpose, study design and methods of analysis, sample size and category of health worker and sex/gender-segregated, key findings, and research gaps indicated by the author/s. Considering the iterative nature of the scoping review abstraction, the Excel sheet will be revised after the pilot test (Peters et al., 2020). The extraction of the stated information will be carryout by two reviewers. Tables will be created for different sources and the outcome of specified aspects of the information.

Data Analysis

Considering the objectives and intent of the scoping review, we will analyze the quantitative data utilizing descriptive statistics, including frequency and proportions, which

will be presented in table and graphs, and qualitative data will follow a description of the concepts and synthesis of themes (Peters et al., 2020).

Presentation of Results

The results section will follow the PRISMA-ScR checklist and be divided into two sections (Peters et al., 2020; Tricco et al., 2018). The first section will describe the search strategy and selection process results, which will also be presented as a PRISMA flow diagram. The second section will focus on responding to research questions for the scoping review, utilizing descriptions, themes, tables, or graphs in detail to identify evidence gaps and map available evidence (Peters et al., 2020).

Conclusion

The last step will summarize the evidence in relation to the purpose of the review leading to the conclusion and implications of the findings (Aromataris & Munn, 2020) on policy and practice.

References

Abed, M., Morris, E., & Sobers-Grannum, N. (2016). Workplace violence against medical staff in healthcare facilities in Barbados. *Occupational Medicine, 66*(7), 580–583. https://doi.org/10.1093/occmed/kqw073

Arksey, H., & O'Malley, L. (2005). Scoping studies: Towards a methodological framework. *International Journal of Social Research Methodology*, *8*(1), 19–32. https://doi.org/10.1080/1364557032000119616

Arnetz, J. E., Hamblin, L., Russell, J., Upfal, M. J., Luborsky, M., Janisse, J., & Essenmacher, L. (2017). Preventing patient-to-worker violence in hospitals: Outcome of a randomized controlled intervention. *Journal of Occupational and Environmental Medicine*, *59*(1), 18–27. https://doi.org/10.1097/JOM.0000000000000909

Aromataris, E., & Munn, Z. (Eds.). (2020). *JBI Manual for Evidence Synthesis*. JBI. https://doi.org/10.46658/JBIMES-20-01

Asi, Y. M., & Williams, C. (2020). Equality through Innovation: Promoting Women in the Workplace in Low- and Middle-Income Countries with Health Information Technology. *Journal of Social Issues*, *76*(3), 721–743. https://doi.org/10.1111/josi.12394

Baig, L., Tanzil, S., Shaikh, S., Hashmi, I., Khan, M. A., & Polkowski, M. (2018). Effectiveness of training on de-escalation of violence and management of aggressive behavior faced by health care providers in a public sector hospital of Karachi. *Pakistan Journal of Medical Sciences*, *34*(2), 294–299. https://doi.org/10.12669/pjms.342.14432

Ceballos, J. B., Frota, O. P., Nunes, H. F. S. S., Ávalos, P. L., Krügel, C. de C., Ferreira Júnior, M. A., & Teston, E. F. (2020). Physical violence and verbal abuse against nurses working with risk stratification: Characteristics, related factors, and

consequences. *Revista Brasileira de Enfermagem*, *73*(suppl 5), e20190882. https://doi.org/10.1590/0034-7167-2019-0882

Ceravolo, D. J., Schwartz, D. G., Foltz-Ramos, K. M., & Casenter, J. (2012). Strengthening communication to overcome lateral violence. *Journal of Nursing Management, 20*, 599–606. https://doi.org/10.1111/j.1365-2834.2012.01402.x

Child, R. J. H., & Mentes, J. C. (2010). Violence Against Women: The Phenomenon of Workplace Violence Against Nurses. *Issues in Mental Health Nursing*, *31*(2), 89–95. https://doi.org/10.3109/01612840903267638

Darawad, M. W., Al-Hussami, M., Saleh, A. M., Mustafa, W. M., & Odeh, H. (2015). Violence Against Nurses in Emergency Departments in Jordan: Nurses' Perspective. *Workplace Health & Safety*, *63*(1), 9–17. https://doi.org/10.1177/2165079914565348

di Martino, V. (2003). *Workplace violence in the health sector*. https://cdn.who.int/media/docs/default-source/documents/violence-against-health-workers/wvstresspaper.pdf?sfvrsn=2afb632b_2

George, A. S., McConville, F. E., de Vries, S., Nigenda, G., Sarfraz, S., & McIsaac, M. (2020). Violence against female health workers is tip of iceberg of gender power imbalances. *BMJ*, m3546. https://doi.org/10.1136/bmj.m3546

Guay, S., Goncalves, J., & Jarvis, J. (2014). Verbal violence in the workplace according to victims' sex—A systematic review of the literature. *Aggression and Violent Behavior*, *19*(5), 572–578. https://doi.org/10.1016/j.avb.2014.08.001

Han, C.-Y., Lin, C.-C., Barnard, A., Hsiao, Y.-C., Goopy, S., & Chen, L.-C. (2017). Workplace violence against emergency nurses in Taiwan: A phenomenographic study. *Nursing Outlook*, *65*(4), 428–435. https://doi.org/10.1016/j.outlook.2017.04.003

International Labour Organization, International Council of Nurses, World Health Organization, & Public Services International. (2002). *Framework guidelines for addressing workplace violence in the health sector.* International Labour Office. http://www.icn.ch/images/stories/documents/pillars/sew/sew_framework_guidelines_for_addressing_workplace_violence.pdf

Jiao, M., Ning, N., Li, Y., Gao, L., Cui, Y., Sun, H., Kang, Z., Liang, L., Wu, Q., & Hao, Y. (2015). Workplace violence against nurses in Chinese hospitals: A cross-sectional survey. *BMJ Open*, *5*(3), e006719–e006719. https://doi.org/10.1136/bmjopen-2014-006719

Lanctôt, N., & Guay, S. (2014). The aftermath of workplace violence among healthcare workers: A systematic literature review of the consequences. *Aggression and Violent Behavior*, *19*(5), 492–501. https://doi.org/10.1016/j.avb.2014.07.010

Levac, D., Colquhoun, H., & O'Brien, K. K. (2010). Scoping studies: Advancing the methodology. *Implementation Science : IS*, *5*, 69. https://doi.org/10.1186/1748-5908-5-69

Liu, J., Gan, Y., Jiang, H., Li, L., Dwyer, R., Lu, K., Yan, S., Sampson, O., Xu, H., Wang, C., Zhu, Y., Chang, Y., Yang, Y., Yang, T., Chen, Y., Song, F., & Lu, Z. (2019). Prevalence of workplace violence against healthcare workers: A systematic review and meta-analysis. *Occupational and Environmental Medicine*, *76*(12), 927–937. https://doi.org/10.1136/oemed-2019-105849

Newman, C. J., de Vries, D. H., d'Arc Kanakuze, J., & Ngendahimana, G. (2011). Workplace violence and gender discrimination in Rwanda's health workforce: Increasing safety and gender equality. *Human Resources for Health*, *9*(1), 19. https://doi.org/10.1186/1478-4491-9-19

Peters, M. D. J., Marnie, C., Tricco, A. C., Pollock, D., Munn, Z., Alexander, L., McInerney, P., Godfrey, C. M., & Khalil, H. (2020). Updated methodological guidance for the conduct of scoping reviews. *JBI Evidence Synthesis*, *18*(10), 2119–2126. https://doi.org/10.11124/JBIES-20-00167

Tricco, A. C., Lillie, E., Zarin, W., O'Brien, K. K., Colquhoun, H., Levac, D., Moher, D., Peters, M. D. J., Horsley, T., Weeks, L., Hempel, S., Akl, E. A., Chang, C., McGowan, J., Stewart, L., Hartling, L., Aldcroft, A., Wilson, M. G., Garritty, C., … Straus, S. E. (2018). PRISMA Extension for Scoping Reviews (PRISMA-ScR): Checklist and Explanation. *Annals of Internal Medicine*, *169*(7), 467–473. https://doi.org/10.7326/M18-0850

WHO. (2016). *Global strategy on human resources for health: Workforce 2030*. World Health Organization. https://apps.who.int/iris/bitstream/handle/10665/250368/9789241511131-eng.pdf?sequence=1
