# Supplementary material for: Perpetrators of gender-based workplace violence amongst nurses and physicians–A scoping review of the literature
Source: PLOS Glob Public Health. 2024 Sep 6;4(9):e0003646. doi: 10.1371/journal.pgph.0003646 (PMC11379169; doi:10.1371/journal.pgph.0003646)
Supplement: S4 Text — (PDF) [file pgph.0003646.s005.pdf]

WPV among healthcare professionals globally for shortlisted articles October 9

Search studies

?

- Latest changes
- Explore knowledge base
- Register for a webinar

FA

Fuseini Adam

fuseini.adam@mail.utoronto.ca

- Profile
- Billing and planCustom plan

- **Organizations**

- UL
  University of Toronto Libraries

- Sign out

Full text review

- Screen references0
- Resolve conflicts0
- Awaiting other reviewer0
- Excluded references70

☑ All

- Filter
- Tags
  - Tag with:
  -
  - ☐ Awaiting classification
  - ☐ Ongoing study
-

Display: 25

Most relevant

	Excluded On	Reason
☐ #298 - Sadrabad 2019<br>Residents' Experiences of Abuse and Harassment in Emergency Departments	16/11/21	Wrong outcomes

	Excluded On	Reason	Ne he

Sadrabad, Akram Zolfaghari; Bidarizerehpoosh, Farahnaz; Farahmand Rad, Reza; Kariman, Hamid; Hatamabadi, Hamidreza; Alimohammadi, Hossein

Journal of interpersonal violence 2019;34(3):642-652

2019

DOI: 10.1177/0886260516645575 · Ref ID: 38243

Full text Abstract

- 1.pdf
  Primary
- Full text uploaded by Graham Dozois
- Manage full text

1 Note History Duplicate Move to Full text

#110 - Yasukawa 2014
The perception and experience of gender-based discrimination related to professional advancement among Japanese physicians

Yasukawa, Kosuke; Nomura, Kyoko

The Tohoku journal of experimental medicine 2014;232(1):35-42

2014

Ref ID: 38371 — Excluded On: 06/11/21 — Reason: Wrong outcomes

Full text Abstract

- a.pdf
  Primary
- Full text uploaded by Graham Dozois
- Manage full text

1 Note History Duplicate Move to Full text

#158 - Frimpong 2016 — Excluded On: 06/11/21 — Reason: Academic Thesis, not published
Men in a female-dominated profession: The lived experiences of Ghanaian male nurses in the United States

Frimpong, Daniel Kwadjo

Men in a Female-dominated Profession: The Lived Experiences of Ghanaian Male Nurses in the United States 2016;():1-1

2016

Ref ID: 38337

Upload full text Abstract

	Excluded On	Reason
1 Note History Duplicate Move to Full text		
#17 - Spiegel 2010<br>Policy and attitude-related reasons for gender disparity in post allocation for graduate medical education in Austria<br><br>Spiegel, Wolfgang; Kamenski, Gustav; Sibitz, Ingrid; Schneider, Barbara; Maier, Manfred<br><br>Medical Teacher 2010;32(2):e78-84<br><br>2010<br><br>DOI: 10.3109/01421590903202488 · Ref ID: 38491<br><br>Full text Abstract<br><br>• a.pdf<br>Primary<br>• Full text uploaded by Graham Dozois<br>• Manage full text<br><br>3 Notes History Duplicate Move to Full text	16/11/21	Wrong outcomes
#303 - Wheeler 2019<br>Physician and Trainee Experiences With Patient Bias<br><br>Wheeler, M.; de Bourmont, S.; Paul-Emile, K.; Pfeffinger, A.; McMullen, A.; Critchfield, J. M.; Fernandez, A.<br><br>JAMA internal medicine 2019;179(12):1678-1685<br><br>2019<br><br>DOI: 10.1001/jamainternmed.2019.4122 · Ref ID: 38227<br><br>Full text Abstract<br><br>• 4.pdf<br>Primary<br>• Full text uploaded by Graham Dozois<br>• Manage full text<br><br>1 Note History Duplicate Move to Full text	14/11/21	Wrong outcomes
#242 - Powers 2018<br>The Lived Experience of Being a Male Nursing Student: Implications for Student Retention and Success<br><br>Powers, Kelly; Herron, Elizabeth K.; Sheeler, Cory; Sain, Amber	04/11/21	Wrong outcomes

	Excluded On	Reason
Journal of professional nursing : official journal of the American Association of Colleges of Nursing 2018;34(6):475-482<br><br>2018<br><br>DOI: 10.1016/j.profnurs.2018.04.002 · Ref ID: 38282<br>Upload full text Abstract<br>1 Note History Duplicate Move to Full text		
#320 - Chow 2020<br>Gender Discrimination Among Academic Physicians<br><br>Chow, Candace J.; Millar, Morgan M.; Lopez, Ana Maria<br><br>Women's health reports (New Rochelle, N.Y.) 2020;1(1):203-211<br><br>2020<br><br>DOI: 10.1089/whr.2020.0031 · Ref ID: 38184<br><br>Full text Abstract<br><br>• a.pdf<br>Primary<br>• Full text uploaded by Graham Dozois<br>• Manage full text<br><br>Note History Duplicate Move to Full text	11/11/21	Wrong outcomes
#372 - Urheim 2020<br>Violence rate dropped during a shift to individualized patient-oriented care in a high security forensic psychiatric ward<br><br>Urheim, R.; Palmstierna, T.; Rypdal, K.; Gjestad, R.; Senneseth, M.; Mykletun, A.<br><br>Bmc Psychiatry 2020;20(1):<br><br>2020<br><br>DOI: 10.1186/s12888-020-02524-0 · Ref ID: 38135<br><br>Full text Abstract<br><br>• a.pdf<br>Primary<br>• Full text uploaded by Graham Dozois<br>• Manage full text<br><br>1 Note History Duplicate Move to Full text	14/11/21	Wrong outcomes
#230 - Halley 2018	13/11/21	Wrong outcomes

	Excluded On	Reason
Physician mothers' experience of workplace discrimination: a qualitative analysis<br><br>Halley, Meghan C.; Rustagi, Alison S.; Torres, Jeanette S.; Linos, Elizabeth; Plaut, Victoria; Mangurian, Christina; Choo, Esther; Linos, Eleni<br><br>BMJ (Clinical research ed.) 2018;363():k4926<br><br>2018<br><br>DOI: 10.1136/bmj.k4926 · Ref ID: 38293<br><br>Full text Abstract<br><br>• 1e.pdf<br>Primary<br>• Full text uploaded by Graham Dozois<br>• Manage full text<br><br>1 Note History Duplicate Move to Full text		
#245 - Shapiro 2018<br>Midwifery student reactions to workplace violence<br><br>Shapiro, J.; Boyle, M. J.; McKenna, L.<br><br>Women and Birth 2018;31(1):E67-E71<br><br>2018<br><br>☐ DOI: 10.1016/j.wombi.2017.06.020 · Ref ID: 38242<br><br>Full text Abstract<br><br>• 1c.pdf<br>Primary<br>• Full text uploaded by Graham Dozois<br>• Manage full text<br><br>Note History Duplicate Move to Full text	13/11/21	Wrong outcomes
☐ #32 - Eliason 2011<br>Lesbian, Gay, Bisexual, Transgender, and Queer/Questioning Nurses' Experiences in the Workplace: Official Journal of the American Association of Colleges of Nurses<br><br>Eliason, Michele J. PhD; DeJoseph, Jeanne PhD C. N. M.; Dibble, Suzanne DNSc R. N.; Deevey, Sharon PhD R. N.; Chinn, Peggy PhD R. N.<br><br>Journal of Professional Nursing 2011;27(4):237<br><br>2011	13/11/21	Wrong patient population

	Excluded On	Reason
DOI: 10.1016/j.profnurs.2011.03.003 · Ref ID: 38473<br>Full text Abstract<br>• a.pdf<br>Primary<br>• Full text uploaded by Graham Dozois<br>• Manage full text<br>Note History Duplicate Move to Full text		
#229 - Gramling 2018<br>Effectiveness of Conducted Electrical Weapons to Prevent Violence-Related Injuries in the Hospital: JEN<br><br>Gramling, Joshua J.; McGovern, Patricia M.; Church, Timothy R.; Nachreiner, Nancy M.; Gaugler, Joseph E.<br><br>Journal of Emergency Nursing 2018;44(3):249-257<br><br>2018<br><br>DOI: 10.1016/j.jen.2017.06.008 · Ref ID: 38294<br><br>Upload full text Abstract<br>1 Note History Duplicate Move to Full text	06/11/21	Wrong patient population
#231 - HedayatiEmam 2018<br>Workplace Violence against Residents in Emergency Department and Reasons for not Reporting Them; a Cross Sectional Study<br><br>Hedayati Emam, Gilava; Alimohammadi, Hossein; Zolfaghari Sadrabad, Akram; Hatamabadi, Hamidreza<br><br>Emergency (Tehran, Iran) 2018;6(1):e7<br><br>2018<br><br>Ref ID: 38292<br><br>Upload full text Abstract<br>1 Note History Duplicate Move to Full text	06/11/21	Wrong outcomes
#145 - Zahid 2015<br>Gender bias in training of medical students in obstetrics and gynaecology: a myth or reality?<br><br>Zahid, Akmal Z. Mohd; Ismail, Zaliha; Abdullah, Bahiyah; Daud, Suzanna<br><br>European journal of obstetrics, gynecology, and reproductive biology 2015;186():17-21<br><br>2015	11/11/21	Wrong outcomes

	Excluded On	Reason
DOI: 10.1016/j.ejogrb.2014.12.018 · Ref ID: 38385<br>Full text Abstract<br>• 1.pdf<br>Primary<br>• Full text uploaded by Graham Dozois<br>• Manage full text<br>3 Notes History Duplicate Move to Full text		
#446 - Zhao 2021<br>Patient violence, physicians treatment decisions, and patient welfare: Evidence from China<br><br>Zhao, Xin; Li, Xiaoxue; Torgler, Benno; Dulleck, Uwe<br><br>Health Economics 2021;30(6):1461-1479<br><br>2021<br><br>DOI: 10.1002/hec.4260 · Ref ID: 38065<br><br>Full text Abstract<br>• hec.4260.pdf<br>Primary<br>• Full text uploaded by Graham Dozois<br>• Manage full text<br><br>Note History Duplicate Move to Full text	13/11/21	Wrong outcomes
#341 - Ko 2020<br>Determinants of Workplace Bullying Types and Their Relationship With Depression Among Female Nurses<br><br>Ko, Ying-Ying; Liu, Yi; Wang, Chi-Jane; Liao, Hsiu-Yun; Liao, Yu-Mei; Chen, Hsing-Mei<br><br>The journal of nursing research : JNR 2020;28(3):e92<br><br>2020<br><br>DOI: 10.1097/JNR.0000000000000367 · Ref ID: 38157<br><br>Full text Abstract<br>• Determinants_of_Workplace_Bullying_Types_and_Their.9.pdf<br>Primary<br>• Full text uploaded by Graham Dozois<br>• Manage full text	11/11/21	Wrong outcomes

	Excluded On	Reason
Note History Duplicate Move to Full text		
#428 - Pendleton 2021<br>A multi-institutional study of patient-derived gender-based discrimination experienced by resident physicians<br><br>Pendleton, Anna Alaska; McKinley, Sophia K.; Pendleton, Virginia E.; Ott, Qi C.; Petrusa, Emil R.; Srivastava, Sunita D.; Lillemoe, Keith D.; Ferrone, Cristina R.<br><br>American Journal of Surgery 2021;221(2):309-314<br><br>2021<br><br>DOI: 10.1016/j.amjsurg.2020.10.015 · Ref ID: 38079<br><br>Full text Abstract<br><br>• e.pdf<br>Primary<br>• Full text uploaded by Graham Dozois<br>• Manage full text<br><br>1 Note History Duplicate Move to Full text	10/11/21	Wrong outcomes
#359 - Procentese 2020<br>Downside: The Perpetrator of Violence in the Representations of Social and Health Professionals<br><br>Procentese, Fortuna; Fasanelli, Roberto; Carnevale, Stefania; Esposito, Ciro; Pisapia, Noemi; Arcidiacono, Caterina; Napoli, Immacolata Di<br><br>International Journal of Environmental Research and Public Health 2020;17(19):<br><br>2020<br><br>DOI: 10.3390/ijerph17197061 · Ref ID: 38189<br><br>Full text Abstract<br><br>• ad.pdf<br>Primary<br>• Full text uploaded by Graham Dozois<br>• Manage full text<br><br>1 Note History Duplicate Move to Full text	13/11/21	Wrong patient population
#339 - Kim 2020<br>Exploring barriers and facilitators for successful transition in new graduate nurses: A mixed methods study	11/11/21	Wrong outcomes

	Excluded On	Reason
Kim, J. H.; Shin, H. S.<br><br>Journal of Professional Nursing 2020;36(6):560-568<br><br>2020<br><br>DOI: 10.1016/j.profnurs.2020.08.006 · Ref ID: 38159<br><br>Full text Abstract<br><br>• 5.pdf<br>Primary<br>• Full text uploaded by Graham Dozois<br>• Manage full text<br>Note History Duplicate Move to Full text		
#87 - Ogundipe 2013<br>Violence in the emergency department: a multicentre survey of nurses' perceptions in Nigeria<br><br>Ogundipe, K. O.; Etonyeaku, A. C.; Adigun, I.; Ojo, E. O.; Aladesanmi, T.; Taiwo, J. O.; Obimakinde, O. S.<br><br>Emergency Medicine Journal 2013;30(9):758-762<br><br>2013<br><br>DOI: 10.1136/emermed-2012-201541 · Ref ID: 38420<br><br>Full text Abstract<br><br>• Violence in the emergency department- a multicentre survey of nurses' perceptions in Nigeria.pdf<br>Primary<br>• Full text uploaded by Basnama ayaz<br>• Manage full text<br><br>Note History Duplicate Move to Full text	06/11/21	Wrong outcomes
#139 - Sedgwick 2015<br>Exploring masculinity and marginalization of male undergraduate nursing students' experience of belonging during clinical experiences<br><br>Sedgwick, Monique G.; Kellett, Peter<br><br>The Journal of nursing education 2015;54(3):121-9<br><br>2015<br><br>DOI: 10.3928/01484834-20150218-15 · Ref ID: 38358<br><br>Full text Abstract	11/11/21	Wrong outcomes

	Excluded On	Reason
- 1.pdf<br>Primary<br>- Full text uploaded by Graham Dozois<br>- Manage full text<br>2 Notes History Duplicate Move to Full text		
#176 - Webster 2016<br>The erasure of gender in academic surgery: a qualitative study<br><br>Webster, Fiona; Rice, Kathleen; Christian, Jennifer; Seemann, Natashia; Baxter, Nancy; Moulton, Carol-Anne; Cil, Tulin<br><br>American Journal of Surgery 2016;212(4):559-565<br><br>2016<br><br>DOI: 10.1016/j.amjsurg.2016.06.006 · Ref ID: 38318<br><br>Full text Abstract<br><br>- a.pdf<br>Primary<br>- Full text uploaded by Graham Dozois<br>- Manage full text<br><br>1 Note History Duplicate Move to Full text	11/11/21	Wrong outcomes
#284 - Krull 2019<br>STAFF PERCEPTION OF INTERPROFESSIONAL SIMULATION FOR VERBAL DE-ESCALATION AND RESTRAINT APPLICATION TO MITIGATE VIOLENT PATIENT BEHAVIORS IN THE EMERGENCY DEPARTMENT<br><br>Krull, W.; Gusenius, T. M.; Germain, D.; Schnepper, L.<br><br>Journal of Emergency Nursing 2019;45(1):24-30<br><br>2019<br><br>DOI: 10.1016/j.jen.2018.07.001 · Ref ID: 38212<br><br>Full text Abstract<br><br>- aa.pdf<br>Primary<br>- Full text uploaded by Graham Dozois<br>- Manage full text<br><br>1 Note History Duplicate Move to Full text	16/11/21	Wrong outcomes
#199 - Karatas 2017	11/11/21	Wrong outcomes

	Excluded On	Reason
A Study of Bullying Against Nursing Students<br><br>Karatas, Hulya; Ozturk, Candan; Bektas, Murat<br><br>The journal of nursing research : JNR 2017;25(3):198-202<br><br>2017<br><br>DOI: 10.1097/JNR.0000000000000144 · Ref ID: 38309<br><br>Full text Abstract<br><br>• A_Study_of_Bullying_Against_Nursing_Students.4.pdf Primary<br>• Full text uploaded by Basnama ayaz<br>• Manage full text<br><br>Note History Duplicate Move to Full text		
#152 - Chadaga 2016<br>Bullying in the American Graduate Medical Education System: A National Cross-Sectional Survey<br><br>Chadaga, Amar R.; Villines, Dana; Krikorian, Armand<br><br>Plos One 2016;11(3):e0150246<br><br>2016<br><br>DOI: 10.1371/journal.pone.0150246 · Ref ID: 38357<br><br>Full text Abstract<br><br>• 4.pdf Primary<br>• Full text uploaded by Graham Dozois<br>• Manage full text<br><br>1 Note History Duplicate Move to Full text	11/11/21	Wrong outcomes
#82 - Khademloo 2013<br>Health care violence and abuse towards nurses in hospitals in north of Iran<br><br>Khademloo, Mohammad; Moonesi, Fatemeh Sheikh; Gholizade, Hamed<br><br>Global journal of health science 2013;5(4):211-6<br><br>2013<br><br>DOI: 10.5539/gjhs.v5n4p211 · Ref ID: 38455<br><br>Full text Abstract	15/11/21	Wrong outcomes

	Excluded On	Reason
• 4.pdf<br>Primary<br>• Full text uploaded by Graham Dozois<br>• Manage full text<br>1 Note History Duplicate Move to Full text		
#155 - Fasanya 2016<br>Workplace Violence and Safety Issues in Long-Term Medical Care Facilities: Nurses' Perspectives<br><br>Fasanya, Bankole K.; Dada, Emmanuel A.<br><br>Safety and health at work 2016;7(2):97-101<br><br>2016<br><br>DOI: 10.1016/j.shaw.2015.11.002 · Ref ID: 38354<br><br>Full text Abstract<br><br>• 1-s2.0-S2093791115000955-main.pdf<br>Primary<br>• Full text uploaded by Graham Dozois<br>• Manage full text<br><br>Note History Duplicate Move to Full text	13/11/21	Wrong outcomes
#248 - Yuh-Hsuan 2018<br>Exploration of the Association Between Workplace Bullying and Attitudes Toward Patient Safety in Female Nurses<br><br>Yuh-Hsuan, Lin; Shu, Tai; Sheen, Hsiao; Chiou-Fen, Lin; Chyn-Yng, Yang; Min-Huey, Chung<br><br>Journal of Nursing 2018;65(1):51-60<br><br>2018<br><br>Ref ID: 38269<br><br>Full text Abstract<br><br>• Exploration of the Association Between Workplace Bullying and Attitudes Toward Patient Safety in Female Nurses.pdf<br>Primary<br>• Full text uploaded by Basnama ayaz<br>• Manage full text<br><br>1 Note History Duplicate Move to Full text	06/11/21	full text not in English
#386 - Adler 2021	12/11/21	Wrong patient population

	Excluded On	Reason
Sexual Harassment by Patients, Clients, and Residents: Investigating Its Prevalence, Frequency and Associations with Impaired Well-Being among Social and Healthcare Workers in Germany<br><br>Adler, Mareike; Vincent-Hoper, Sylvie; Vaupel, Claudia; Gregersen, Sabine; Schablon, Anja; Nienhaus, Albert<br><br>International Journal of Environmental Research and Public Health 2021;18(10):<br><br>2021<br><br>DOI: 10.3390/ijerph18105198 · Ref ID: 38119<br><br>Full text Abstract<br><br>• 3.pdf Primary<br>• Full text uploaded by Graham Dozois<br>• Manage full text<br><br>1 Note History Duplicate Move to Full text		
#4 - Chang 2010<br>The effect of student gender on the obstetrics and gynecology clerkship experience<br><br>Chang, Judy C.; Odrobina, Michele R.; McIntyre-Seltman, Kathleen<br><br>Journal of women's health (2002) 2010;19(1):87-92<br><br>2010<br><br>DOI: 10.1089/jwh.2009.1357 · Ref ID: 38507<br><br>Full text Abstract<br><br>• The Effect of Student Gender on the Obstetrics and Gynecology Clerkship Experience.pdf Primary<br>• Full text uploaded by Basnama ayaz<br>• Manage full text<br><br>1 Note History Duplicate Move to Full text	16/11/21	Wrong outcomes
#31 - DeSouza 2011<br>Frequency rates and correlates of contrapower harassment in higher education<br><br>DeSouza, Eros R.<br><br>Journal of interpersonal violence 2011;26(1):158-88	16/11/21	Wrong patient population

	Excluded On	Reason
2011<br><br>DOI: 10.1177/0886260510362878 · Ref ID: 38474<br>Full text Abstract<br><br>- a.pdf<br>Primary<br>- Full text uploaded by Graham Dozois<br>- Manage full text<br><br>1 Note History Duplicate Move to Full text		
#78 - Cochran 2013<br>Perceived gender-based barriers to careers in academic surgery<br><br>Cochran, Amalia; Hauschild, Tricia; Elder, William B.; Neumayer, Leigh A.; Brasel, Karen J.; Crandall, Marie L.<br><br>American Journal of Surgery 2013;206(2):263-8<br><br>2013<br><br>DOI: 10.1016/j.amjsurg.2012.07.044 · Ref ID: 38430<br><br>Full text Abstract<br><br>- Perceived gender-based barriers to careers in academic surgery.pdf<br>Primary<br>- Full text uploaded by Basnama ayaz<br>- Manage full text<br><br>Note History Duplicate Move to Full text	13/11/21	Wrong outcomes
#282 - Kara 2019<br>Gender Differences in Attitudes Toward Sexual Harassment of Health Care Employees: A Turkish Case Study<br><br>Kara, Derya; Toygar, Sukru Anil<br><br>Journal of interpersonal violence 2019;34(17):3574-3591<br><br>2019<br><br>DOI: 10.1177/0886260518815711 · Ref ID: 38214<br><br>Upload full text Abstract<br>1 Note History Duplicate Move to Full text	13/11/21	Wrong outcomes
#296 - Rayan 2019<br>Stress, Workplace Violence, and Burnout in Nurses Working in King Abdullah Medical City During Al-Hajj Season	11/11/21	Wrong outcomes

	Excluded On	Reason
Rayan, A.; Sisan, M.; Baker, O.<br><br>Journal of Nursing Research 2019;27(3):<br><br>2019<br><br>DOI: 10.1097/jnr.0000000000000291 · Ref ID: 38232<br><br>Full text Abstract<br><br>• 4.pdf<br>Primary<br>• Full text uploaded by Graham Dozois<br>• Manage full text<br>Note History Duplicate Move to Full text		
#154 - Esinte 2016<br>Nigerian male nurses' perceptions of nursing: A qualitative case study<br><br>Esinte, Joseph<br><br>Nigerian Male Nurses' Perceptions of Nursing: A Qualitative Case Study 2016;():1-1<br><br>2016<br><br>Ref ID: 38355<br><br>Upload full text Abstract<br>1 Note History Duplicate Move to Full text	13/11/21	Wrong outcomes
#270 - Englund 2019<br>Nontraditional Students' Perceptions of Marginalization in Baccalaureate Nursing Education: Pushed to the Periphery<br><br>Englund, Heather M.<br><br>Nurse educator 2019;44(3):164-169<br><br>2019<br><br>DOI: 10.1097/NNE.0000000000000581 · Ref ID: 38226<br><br>Full text Abstract<br><br>• 12.pdf<br>Primary<br>• Full text uploaded by Graham Dozois<br>• Manage full text<br><br>2 Notes History Duplicate Move to Full text	06/11/21	Wrong patient population

	Excluded On	Reason
#85 - Lepping 2013<br>Percentage prevalence of patient and visitor violence against staff in high-risk UK medical wards<br><br>Lepping, Peter; Lanka, Srinivas Vn; Turner, Jim; Stanaway, Stephen Ers; Krishna, Murali<br><br>Clinical medicine (London, England) 2013;13(6):543-6<br><br>2013<br><br>DOI: 10.7861/clinmedicine.13-6-543 · Ref ID: 38424<br><br>Full text Abstract<br><br>• 1.pdf<br>Primary<br>• Full text uploaded by Graham Dozois<br>• Manage full text<br><br>1 Note History Duplicate Move to Full text	06/11/21	Wrong outcomes
#214 - Agrawal 2018<br>Assessing workplace discrimination among medical practitioners in Western Sydney<br><br>Agrawal, Ravindra; Foresti, Katia; Rajadurai, Jeremy; Zubaran, Carlos<br><br>Australasian psychiatry : bulletin of Royal Australian and New Zealand College of Psychiatrists 2018;26(5):491-495<br><br>2018<br><br>DOI: 10.1177/1039856218772248 · Ref ID: 38271<br><br>Full text Abstract<br><br>• 7.pdf<br>Primary<br>• Full text uploaded by Graham Dozois<br>• Manage full text<br><br>2 Notes History Duplicate Move to Full text	06/11/21	Wrong outcomes
#218 - Carnevale 2018<br>"The Good Ole' Girls' Nursing Club": The Male Student Perspective<br><br>Carnevale, Teresa; Priode, Kimberly<br><br>Journal of transcultural nursing : official journal of the Transcultural Nursing Society 2018;29(3):285-291<br><br>2018	11/11/21	Wrong outcomes

	Excluded On	Reason
DOI: 10.1177/1043659617703163 · Ref ID: 38250<br>Full text Abstract<br>• aa.pdf<br>Primary<br>• Full text uploaded by Graham Dozois<br>• Manage full text<br>1 Note History Duplicate Move to Full text		
#204 - Martinez 2017<br>Fleeing the Ivory Tower: Gender Differences in the Turnover Experiences of Women Faculty<br><br>Martinez, Larry R.; O'Brien, Katharine R.; Hebl, Michelle R.<br><br>Journal of women's health (2002) 2017;26(5):580-586<br><br>2017<br><br>DOI: 10.1089/jwh.2016.6023 · Ref ID: 38324<br><br>Full text Abstract<br>• a.pdf<br>Primary<br>• Full text uploaded by Graham Dozois<br>• Manage full text<br><br>1 Note History Duplicate Move to Full text	11/11/21	Wrong outcomes
#378 - Wesolowska 2020<br>Nativity status and workplace discrimination in registered nurses: Testing the mediating role of psychosocial work characteristics<br><br>Wesolowska, Karolina; Elovainio, Marko; Komulainen, Kaisla; Hietapakka, Laura; Heponiemi, Tarja<br><br>Journal of Advanced Nursing 2020;76(7):1594-1602<br><br>2020<br><br>DOI: 10.1111/jan.14361 · Ref ID: 38129<br><br>Full text Abstract<br>• 2.pdf<br>Primary<br>• Full text uploaded by Graham Dozois<br>• Manage full text	10/11/21	Wrong outcomes

	Excluded On	Reason
2 Notes History Duplicate Move to Full text		
#197 - Johansen 2017<br>Changes in prevalence of workplace violence against doctors in all medical specialties in Norway between 1993 and 2014: a repeated cross-sectional survey<br><br>Johansen, Ingrid Hjulstad; Baste, Valborg; Rosta, Judith; Aasland, Olaf G.; Morken, Tone<br><br>Bmj Open 2017;7(8):e017757<br><br>2017<br><br>DOI: 10.1136/bmjopen-2017-017757 · Ref ID: 38311<br><br>Full text Abstract<br><br>• aa.pdf<br>Primary<br>• Full text uploaded by Graham Dozois<br>• Manage full text<br><br>Note History Duplicate Move to Full text	16/11/21	Wrong outcomes
#77 - Chipps 2013<br>Workplace Bullying in the OR: Results of a Descriptive Study<br><br>Chipps, E.; Stelmaschuk, S.; Albert, N. M.; Bernhard, L.; Holloman, C.<br><br>AORN journal 2013;98(5):479-493<br><br>2013<br><br>DOI: 10.1016/j.aorn.2013.08.015 · Ref ID: 38431<br><br>Full text Abstract<br><br>• j.aorn.2013.08.015.pdf<br>Primary<br>• Full text uploaded by Graham Dozois<br>• Manage full text<br><br>1 Note History Duplicate Move to Full text	16/11/21	Wrong outcomes
#436 - Silva 2021<br>Gender violence against woman nursing students: a cross-sectional study<br><br>Silva, Luiza Csordas Peixinho da; Hino, Paula; Oliveira, Rebeca Nunes Guedes de; Fernandes, Hugo<br><br>Revista Brasileira De Enfermagem 2021;74(5):e20200539	13/11/21	Wrong setting

	Excluded On	Reason
2021<br><br>DOI: 10.1590/0034-7167-2020-0539 · Ref ID: 38071<br>Full text Abstract<br>• aa.pdf<br>Primary<br>• Full text uploaded by Graham Dozois<br>• Manage full text<br>1 Note History Duplicate Move to Full text		
#33 - Eliason 2011<br>Lesbian, Gay, Bisexual, and Transgender (LGBT) Physicians' Experiences in the Workplace<br><br>Eliason, Michele J. PhD; Dibble, Suzanne L. DNScRN; Robertson, Patricia A. M. D.<br><br>Journal of Homosexuality 2011;58(10):1355<br><br>2011<br><br>DOI: 10.1080/00918369.2011.614902 · Ref ID: 38472<br><br>Upload full text Abstract<br>Note History Duplicate Move to Full text	06/11/21	Wrong patient population
#128 - Ganz 2015<br>Bullying and Its Prevention Among Intensive Care Nurses<br><br>Ganz, Freda DeKeyser; Levy, Hadassa; Khalaila, Rabia; Arad, Dana; Bennaroch, Kochav; Kolpak, Orly; Drori, Yardena; Benbinishty, Julie; Raanan, Ofra<br><br>Journal of nursing scholarship : an official publication of Sigma Theta Tau International Honor Society of Nursing 2015;47(6):505-11<br><br>2015<br><br>DOI: 10.1111/jnu.12167 · Ref ID: 38400<br><br>Full text Abstract<br>• jnu.12167.pdf<br>Primary<br>• Full text uploaded by Graham Dozois<br>• Manage full text<br><br>Note History Duplicate Move to Full text	06/11/21	Wrong outcomes

	Excluded On	Reason
#294 - Osborn 2019<br>A Survey Study of Female Radiation Oncology Residents' Experiences to Inform Change<br><br>Osborn, Virginia W.; Doke, Kaleigh; Griffith, Kent A.; Jones, Rochelle; Lee, Anna; Maquilan, Genevieve; Masters, Adrianna Henson; Albert, Ashley A.; Dover, Laura L.; Puckett, Lindsay L.; Hentz, Courtney; Kahn, Jenna M.; Colbert, Lauren E.; Barry, Parul N.; Jagsi, Reshma<br><br>International journal of radiation oncology, biology, physics 2019;104(5):999-1008<br><br>2019<br><br>DOI: 10.1016/j.ijrobp.2019.05.013 · Ref ID: 38234<br><br>Full text Abstract<br><br>• 2.pdf<br>Primary<br>• Full text uploaded by Graham Dozois<br>• Manage full text<br><br>1 Note History Duplicate Move to Full text	11/11/21	Wrong outcomes
#223 - Cheng 2018<br>Lived Experiences of Novice Male Nurses in Taiwan<br><br>Cheng, Mei-Li; Tseng, Ying-Hua; Hodges, Eric; Chou, Fan-Hao<br><br>Journal of transcultural nursing : official journal of the Transcultural Nursing Society 2018;29(1):46-53<br><br>2018<br><br>DOI: 10.1177/1043659616676318 · Ref ID: 38300<br><br>Full text Abstract<br><br>• a.pdf<br>Primary<br>• Full text uploaded by Graham Dozois<br>• Manage full text<br><br>1 Note History Duplicate Move to Full text	13/11/21	Wrong outcomes
#257 - Anjum 2019<br>Antecedents of gender gap in workforce participation: A phenomenology of psychologists and medical doctors in urban Pakistan<br><br>Anjum, Gulnaz; Kamal, Anila; Bilwani, Sania<br><br>Journal of Human Behavior in the Social Environment 2019;29(2):282-299	08/11/21	Wrong outcomes

	Excluded On	Reason
2019<br><br>DOI: 10.1080/10911359.2018.1536576 · Ref ID: 38260<br>Full text Abstract<br><br>• 1.pdf<br>Primary<br>• Full text uploaded by Graham Dozois<br>• Manage full text<br>2 Notes History Duplicate Move to Full text		
#212 - Umoetok 2017<br>Does gender impact on female doctors'experiences in the training and practice of surgery? A single centre study<br><br>Umoetok, F.; Van Wyk, J. M.; Madiba, T. E.<br><br>South African journal of surgery. Suid-Afrikaanse tydskrif vir chirurgie 2017;55(3):8-12<br><br>2017<br><br>Ref ID: 38273<br><br>Full text Abstract<br><br>• 159707-Article Text-414786-1-10-20170803.pdf<br>Primary<br>• Full text uploaded by Graham Dozois<br>• Manage full text<br><br>1 Note History Duplicate Move to Full text	12/11/21	Wrong outcomes
#73 - Yang 2012<br>Psychosocial precursors and physical consequences of workplace violence towards nurses: A longitudinal examination with naturally occurring groups in hospital settings<br><br>Yang, Liu-Qin; Spector, Paul E.; Chang, Chu-Hsiang; Gallant-Roman, Mary; Powell, Julie<br><br>International Journal of Nursing Studies 2012;49(9):1091<br><br>2012<br><br>DOI: 10.1016/j.ijnurstu.2012.03.006 · Ref ID: 38437<br><br>Full text Abstract<br><br>• aw.pdf	13/11/21	Wrong outcomes

	Excluded On	Reason
Primary<br>• Full text uploaded by Graham Dozois<br>• Manage full text<br>Note History Duplicate Move to Full text		
#267 - Campbell 2019<br>Folie du systeme? Preventing Violence Against Nurses in In-patient Psychiatry<br><br>Campbell, Vashti L. S.; Foley, Holly L.; Vianna, Kevin W.; Brunger, Fern<br><br>The Psychiatric quarterly 2019;90(2):413-420<br><br>2019<br><br>DOI: 10.1007/s11126-019-09636-1 · Ref ID: 38254<br><br>Full text Abstract<br><br>• aa.pdf<br>Primary<br>• Full text uploaded by Graham Dozois<br>• Manage full text<br><br>Note History Duplicate Move to Full text	13/11/21	Wrong study design
#93 - Cavanaugh 2014<br>A Longitudinal Study of the Impact of Cumulative Violence Victimization on Comorbid Posttraumatic Stress and Depression Among Female Nurses and Nursing Personnel<br><br>Cavanaugh, C.; Campbell, J.; Messing, J. T.<br><br>Workplace Health & Safety 2014;62(6):224-232<br><br>2014<br><br>DOI: 10.3928/21650799-20140514-01 · Ref ID: 38433<br><br>Full text Abstract<br><br>• a.pdf<br>Primary<br>• Full text uploaded by Graham Dozois<br>• Manage full text<br><br>Note History Duplicate Move to Full text	11/11/21	Wrong intervention
#236 - Miller 2018<br>Gender Differences in Perception of Workplace Experience Among Anesthesiology Residents	13/11/21	Wrong outcomes

	Excluded On	Reason
Miller, Joanna; Katz, Daniel<br><br>The journal of education in perioperative medicine : JEPM 2018;20(1):E618<br><br>2018<br><br>Ref ID: 38287<br><br>Upload full text Abstract<br>1 Note History Duplicate Move to Full text		
#58 - Berry 2012<br>Novice nurse productivity following workplace bullying<br><br>Berry, Peggy A.; Gillespie, Gordon L.; Gates, Donna; Schafer, John<br><br>Journal of nursing scholarship : an official publication of Sigma Theta Tau International Honor Society of Nursing 2012;44(1):80-7<br><br>2012<br><br>DOI: 10.1111/j.1547-5069.2011.01436.x · Ref ID: 38447<br><br>Full text Abstract<br><br>• ar.pdf<br>Primary<br>• Full text uploaded by Graham Dozois<br>• Manage full text<br><br>Note History Duplicate Move to Full text	13/11/21	Wrong outcomes
#66 - Kowalenko 2012<br>Development of a Data Collection Instrument for Violent Patient Encounters against Healthcare Workers<br><br>Kowalenko, Terry; Hauff, Samantha R.; Morden, Peter C.; Smith, Barbara<br><br>The western journal of emergency medicine 2012;13(5):429-33<br><br>2012<br><br>DOI: 10.5811/westjem.2011.12.6795 · Ref ID: 38489<br><br>Full text Abstract<br><br>• eScholarship UC item 451704jb.pdf<br>Primary<br>• Full text uploaded by Graham Dozois<br>• Manage full text<br><br>1 Note History Duplicate Move to Full text	13/11/21	Wrong outcomes

	Excluded On	Reason
#175 - Trahan 2016<br>Coping Strategies of Neurology Nurses Experiencing Abuse From Patients and Families<br><br>Trahan, R. L.; Bishop, S. L.<br><br>Journal of Neuroscience Nursing 2016;48(3):118-123<br><br>2016<br><br>DOI: 10.1097/jnn.0000000000000211 · Ref ID: 38319<br><br>Full text Abstract<br><br>• Coping Strategies of Neurology Nurses Experiencing Abuse From Patients and Families.pdf<br>Primary<br>• Full text uploaded by Graham Dozois<br>• Manage full text<br><br>Note History Duplicate Move to Full text	16/11/21	Wrong outcomes
#95 - Heponiemi 2014<br>The prospective effects of workplace violence on physicians' job satisfaction and turnover intentions: the buffering effect of job control<br><br>Heponiemi, Tarja; Kouvonen, Anne; Virtanen, Marianna; Vanska, Jukka; Elovainio, Marko<br><br>Bmc Health Services Research 2014;14():19<br><br>2014<br><br>DOI: 10.1186/1472-6963-14-19 · Ref ID: 38396<br><br>Full text Abstract<br><br>• 22.pdf<br>Primary<br>• Full text uploaded by Graham Dozois<br>• Manage full text<br><br>Note History Duplicate Move to Full text	06/11/21	Wrong outcomes
#323 - DeWane 2020<br>A commentary on gender bias in dermatology and its perceived impact on career development among women dermatologists<br><br>DeWane, Madeline E.; Grant-Kels, Jane M.<br><br>International Journal of Women's Dermatology 2020;6(5):440-444<br><br>2020	13/11/21	A commentary on women underrepresented due harassment and discrimination

	Excluded On	Reason
DOI: 10.1016/j.ijwd.2020.07.010 · Ref ID: 38181<br>Full text Abstract<br>• q.pdf<br>Primary<br>• Full text uploaded by Graham Dozois<br>• Manage full text<br>Note History Duplicate Move to Full text		
#201 - Kulaylat 2017<br>Perceptions of mistreatment among trainees vary at different stages of clinical training<br><br>Kulaylat, Afif N.; Qin, Danni; Sun, Susie X.; Hollenbeak, Christopher S.; Schubart, Jane R.; Aboud, Antone J.; Flemming, Donald J.; Dillon, Peter W.; Bollard, Edward R.; Han, David C.<br><br>Bmc Medical Education 2017;17(1):14<br><br>2017<br><br>DOI: 10.1186/s12909-016-0853-4 · Ref ID: 38307<br><br>Full text Abstract<br>• af.pdf<br>Primary<br>• Full text uploaded by Graham Dozois<br>• Manage full text<br><br>Note History Duplicate Move to Full text	13/11/21	Wrong outcomes
#39 - Isaac 2011<br>Women leaders: the social world of health care<br><br>Isaac, Carol A.<br><br>Journal of Health Organization and Management 2011;25(2):159-75<br><br>2011<br><br>Ref ID: 38505<br><br>Full text Abstract<br>• Women leaders- the social world of health care.pdf<br>Primary<br>• Full text uploaded by Basnama ayaz<br>• Manage full text	06/11/21	Wrong outcomes

		Excluded On	Reason
	1 Note History Duplicate Move to Full text		
☐	#271 - Evans 2019<br>You have to be twice as good and work twice as hard: a mixed-methods study of perceptions of sexual harassment, assault and women's leadership among female faculty at a research university in the USA<br><br>Evans, Dabney P.; Sales, Jessica M.; Krause, Kathleen H.; Del Rio, Carlos<br><br>Global Health, Epidemiology and Genomics 2019;4():e6<br><br>2019<br><br>DOI: 10.1017/gheg.2019.5 · Ref ID: 38225<br><br>Full text Abstract<br><br>• 2.pdf<br>Primary<br>• Full text uploaded by Graham Dozois<br>• Manage full text<br><br>1 Note History Duplicate Move to Full text	06/11/21	Wrong patient population
☐	#123 - Eslamian 2015<br>Quality of work life and its association with workplace violence of the nurses in emergency departments<br><br>Eslamian, Jalil; Akbarpoor, Ali Akbar; Hoseini, Sayed Abbas<br><br>Iranian journal of nursing and midwifery research 2015;20(1):56-62<br><br>2015<br><br>Ref ID: 38405<br><br>Full text Abstract<br><br>• a.pdf<br>Primary<br>• Full text uploaded by Graham Dozois<br>• Manage full text<br><br>Note History Duplicate Move to Full text	11/11/21	Wrong outcomes
☐	#186 - Bouret 2017<br>Male nurses' lived experiences with lateral violence in the workplace<br><br>Bouret, Joseph<br><br>Male Nurses' Lived Experiences with Lateral Violence in the Workplace 2017;():1-1	11/11/21	Academic Thesis, not published

	Excluded On	Reason
2017<br><br>Ref ID: 38343<br>Full text Abstract<br><br>• a.pdf<br>Primary<br>• Full text uploaded by Graham Dozois<br>• Manage full text<br>Note History Duplicate Move to Full text		
#328 - Findley 2020<br>Interdisciplinary Healthcare Students' Experiences of Intimidation, Harassment, and Discrimination During Training<br><br>Findley, Patricia A.; Harris, Caroline Es<br><br>Journal of Allied Health 2020;49(1):e39-e42<br><br>2020<br><br>Ref ID: 38177<br><br>Full text Abstract<br><br>• 2.pdf<br>Primary<br>• Full text uploaded by Graham Dozois<br>• Manage full text<br><br>3 Notes History Duplicate Move to Full text	09/11/21	Wrong outcomes
#206 - Olender 2017<br>The Relationship Between and Factors Influencing Staff Nurses' Perceptions of Nurse Manager Caring and Exposure to Workplace Bullying in Multiple Healthcare Settings<br><br>Olender, Lynda<br><br>The Journal of nursing administration 2017;47(10):501-507<br><br>2017<br><br>DOI: 10.1097/NNA.0000000000000522 · Ref ID: 38305<br><br>Full text Abstract<br><br>• The Relationship Between and Factors Influencing Staff Nurses' Perceptions of Nurse Manager Caring and Exposure to Workplace Bullying in Multiple Healthcare Settings.pdf<br>Primary	11/11/21	Wrong outcomes

	Excluded On	Reason
• Full text uploaded by Graham Dozois<br>• Manage full text<br>1 Note History Duplicate Move to Full text		
#243 - Ramacciati 2018<br>Violence towards Emergency Nurses. The Italian National Survey 2016: A qualitative study<br><br>Ramacciati, N.; Ceccagnoli, A.; Addey, B.; Rasero, L.<br><br>International Journal of Nursing Studies 2018;81():21-29<br><br>2018<br><br>DOI: 10.1016/j.ijnurstu.2018.01.017 · Ref ID: 38281<br><br>Full text Abstract<br>• 1b.pdf<br>Primary<br>• Full text uploaded by Graham Dozois<br>• Manage full text<br><br>Note History Duplicate Move to Full text	13/11/21	Wrong outcomes
#291 - Moore 2019<br>Burnout and Career Satisfaction in Women Neurologists in the United States<br><br>Moore, Lauren R.; Ziegler, Craig; Hessler, Amy; Singhal, Divya; LaFaver, Kathrin<br><br>Journal of women's health (2002) 2019;28(4):515-525<br><br>2019<br><br>DOI: 10.1089/jwh.2017.6888 · Ref ID: 38237<br><br>Full text Abstract<br>• 1.pdf<br>Primary<br>• Full text uploaded by Graham Dozois<br>• Manage full text<br><br>2 Notes History Duplicate Move to Full text	14/11/21	Wrong outcomes
#369 - Tee 2020<br>Experience of Workplace Violence Toward Nursing Students in Iran: A Qualitative Study<br><br>Tee, Stephen; Valiee, Sina	13/11/21	Wrong outcomes

	Excluded On	Reason
Journal of Forensic Nursing 2020;16(2):83-89<br><br>2020<br><br>DOI: 10.1097/JFN.0000000000000285 · Ref ID: 38138<br>Full text Abstract<br><br>• aaaa.pdf<br>Primary<br>• Full text uploaded by Graham Dozois<br>• Manage full text<br>2 Notes History Duplicate Move to Full text		
#67 - MacIntosh 2012<br>Workplace Bullying Influences Women's Engagement in the Workforce<br><br>MacIntosh, Judith<br><br>Issues in Mental Health Nursing 2012;33(11):762-768<br><br>2012<br><br>☐ DOI: 10.3109/01612840.2012.708701 · Ref ID: 38488<br><br>Full text Abstract<br><br>• aaaaa.pdf<br>Primary<br>• Full text uploaded by Graham Dozois<br>• Manage full text<br><br>1 Note History Duplicate Move to Full text	14/11/21	Wrong setting

- © 2024 Covidence
- Terms
- Privacy

Feedback & Support

Find your answer, fast.

We've got a new Knowledge base that answers many frequently asked questions. Why not try that first?

View the knowledge base OR Send us an email

WPV 2024

Search studies

- Latest changes
- Explore knowledge base
- Register for a webinar

FA

Fuseini Adam

fuseini.adam@mail.utoronto.ca

- Profile
- Billing and planCustom plan

- **Organizations**

- UL
  University of Toronto Libraries

- Sign out

Full text review

- Screen references0
- Resolve conflicts0
- Awaiting other reviewer0
- Excluded references10

All

- Filter
- Tags
  - Tag with:
  - Awaiting classification
  - Ongoing study

Display: 25
Most relevant

	Excluded On	Reason
#9973 - Graham 2021	08/03/24	Wrong study design

	Excluded On	Reason	Ne he
Gender-based Barriers in the Advancement of Women Leaders in Emergency Medicine: A Multi-institutional Qualitative Study.<br><br>Graham, Emily M.; Ferrel, Meganne N.; Wells, Katie M.; Egan, Daniel J.; MacVane, Casey Z.; Gisondi, Michael A.; Burns, Boyd D.; Madsen, Troy E.; Fix, Megan L.<br><br>Western Journal of Emergency Medicine: Integrating Emergency Care with Population Health 2021;22(6):1355-1359<br><br>2021<br><br>DOI: 10.5811/westjem.2021.7.52826<br><br>Full text Abstract<br><br>• qt53c707nt.pdf Primary<br>• Full text uploaded by Covidence (Open Access)<br>• Manage full text<br><br>Note History Duplicate Move to Full text			
#10305 - Gupta 2023<br>Violence and aggression against nurses during the COVID-19 pandemic in Latin America. From the emerging leaders program of the Interamerican Society of Cardiology (SIAC).<br><br>Gupta, Shyla; Garcia-Zamora, Sebastian; Juarez-Lloclla, Jorge; Farina, Juan; Foisy, Melanie; Pulido, Laura; Ramos, Victoria; Merschon, Franco; Parodi, Josefina B; Sanchez, Maria Elena; Munera, Ana; Piskorz, Daniel; Pineiro, Daniel Jose; Tse, Gary; Lopez-Santi, Ricardo; Baranchuk, Adrian<br><br>J Adv Nurs 2023;(7609811, h3l):<br><br>2023<br><br>DOI: 10.1111/jan.15900<br><br>Upload full text Abstract<br>Note History Duplicate Move to Full text	08/03/24	Wrong intervention	
#10694 - Doehring 2023<br>Exploring verbal and physical workplace violence in a large, urban emergency department.<br><br>Doehring, Marla C; Curtice, Hanan; Hunter, Benton R; Oaxaca, Derrick M; Satorius, Ashley; Reed, Kyra; Beckman, Andrew; Vaughn, Tabitha; Palmer, Megan<br><br>Am J Emerg Med 2023;67(aa2, 8309942):1-4<br><br>2023	08/03/24	Wrong outcomes	

	Excluded On	Reason
DOI: 10.1016/j.ajem.2023.01.036<br>Upload full text Abstract<br>Note History Duplicate Move to Full text		
#10018 - Favaro 2021<br>Relationships among sex, empowerment, workplace bullying and job turnover intention of new graduate nurses.<br><br>Favaro, Aaron; Wong, Carol; Oudshoorn, Abe<br><br>Journal of Clinical Nursing (John Wiley & Sons, Inc.) 2021;30(9/10):1273-1284<br><br>2021<br><br>DOI: 10.1111/jocn.15671<br><br>Upload full text Abstract<br>Note History Duplicate Move to Full text	08/03/24	previous analyzed
#10635 - Yu 2023<br>Matters of the Heart: Examining Motivating Factors and Unconscious Bias in the Adult Cardiothoracic Anesthesiology Fellowship.<br><br>Yu, Soojie; Tavarez-Mora, Frank; Milam, Adam J; Misra, Lopa; Aljure, Oscar; Glas, Kathryn; Shillcutt, Sasha<br><br>J Cardiothorac Vasc Anesth 2023;37(7):1160-1168<br><br>2023<br><br>DOI: 10.1053/j.jvca.2023.02.044<br><br>Upload full text Abstract<br>Note History Duplicate Move to Full text	08/03/24	Wrong outcomes
#10774 - Lutzerath 2023<br>Influencing factors on the health of nurses-a regression analysis considering individual and organizational determinants in Germany.<br><br>Lutzerath, Jasmin; Bleier, Hannah; Stassen, Gerrit; Schaller, Andrea<br><br>BMC Health Serv Res 2023;23(1):100<br><br>2023<br><br>DOI: 10.1186/s12913-023-09106-2<br><br>Full text Abstract<br><br>• s12913-023-09106-2.pdf<br>Primary	08/03/24	Wrong outcomes

	Excluded On	Reason
• Full text uploaded by Covidence (Open Access)<br>• Manage full text<br>Note History Duplicate Move to Full text		
#10683 - Hu 2023<br>Specialty Gender Composition and Prevalence of Physician Harassment.<br><br>Hu, Xiaochu; Conrad, Sarah; Dill, Michael<br><br>J Womens Health (Larchmt) 2023;32(4):409-415<br><br>2023<br><br>DOI: 10.1089/jwh.2022.0380<br><br>Upload full text Abstract<br>Note History Duplicate Move to Full text	08/03/24	previous analyzed
#10342 - Berger-Estilita 2024<br>European anesthesiologists' experiences with gender-based mistreatment in the workplace: a secondary multilevel regression analysis.<br><br>Berger-Estilita, Joana; Fritsche, Luana; El-Boghdadly, Kariem; Dias, Claudia Camila; Zdravkovic, Marko<br><br>Korean Journal Anesthesiol 2024;77(1):46-57<br><br>2024<br><br>DOI: 10.4097/kja.23392<br><br>Full text Abstract<br><br>• kja-23392.pdf<br>Primary<br>• Full text uploaded by Covidence (Open Access)<br>• Manage full text<br><br>Note History Duplicate Move to Full text	08/03/24	reported results only for women
#10551 - Benjamin 2023<br>Assessing the Prevalence of Burnout Among Female Microvascular Head and Neck Surgeons.<br><br>Benjamin, Tania; Gulati, Arushi; Zebolsky, Aaron L; Seth, Rahul; Knott, P Daniel; Okuyemi, Oluwafunmilola; Park, Andrea M<br><br>Facial Plast Surg Aesthet Med 2023;25(4):298-303<br><br>2023	08/03/24	Wrong outcomes

	Excluded On	Reason
DOI: 10.1089/fpsam.2022.0312<br>Upload full text Abstract<br>Note History Duplicate Move to Full text		
#10573 - Russell 2023<br>Addressing Sexual Harassment and Gender Bias: Mandatory Modules Are Not Enough.<br><br>Russell, Holly Ann; Sanders, Mechelle; Nofziger, Anne; Fogarty, Colleen T; McDaniel, Susan H; Rosenberg, Tziporah; Fiscella, Kevin; Naumburg, Elizabeth<br><br>Fam Med 2023;55(4):253-258<br><br>☐ 2023<br><br>DOI: 10.22454/FamMed.2023.488622<br><br>Full text Abstract<br><br>• russell0200.pdf Primary<br>• Full text uploaded by Covidence (Open Access)<br>• Manage full text<br><br>Note History Duplicate Move to Full text	08/03/24	Wrong outcomes

- © 2024 Covidence
- Terms
- Privacy

Feedback & Support

Find your answer, fast.

We've got a new Knowledge base that answers many frequently asked questions. Why not try that first?

View the knowledge base OR Send us an email

WPW 2022

Search studies

- Latest changes
- Explore knowledge base
- Register for a webinar

FA

Fuseini Adam

fuseini.adam@mail.utoronto.ca

- Profile
- Billing and planCustom plan

- **Organizations**

- UL
  University of Toronto Libraries

- Sign out

Full text review

- Screen references0
- Resolve conflicts0
- Awaiting other reviewer0
- Excluded references38

All

- Filter
- Tags
  - Tag with:
  -
  - Awaiting classification
  - Ongoing study
-

Display: 25
Most relevant

	Excluded On	Reason
#157 - Alsharari 2022<br>Workplace violence towards emergency nurses: A cross-sectional multicenter study	09/03/23	Wrong outcomes

		Excluded On	Reason	Ne he
	Alsharari, Abdalkarem F.; Abu-Snieneh, Hana M.; Abuadas, Fuad H.; Elsabagh, Nahed E.; Althobaity, Abdulellah; Alshammari, Farhan F.; Alshmemri, Mohammed S.; Aroury, Ammar M.; Alkhadam, Arab Q.; Alatawi, Suliman S.<br><br>Australasian Emergency Care 2022;25(1):48-54<br><br>Australia 2022<br><br>DOI: 10.1016/j.auec.2021.01.004<br><br>Upload full text Abstract<br>Note History Duplicate Move to Full text			
☐	#200 - Rogers 2021<br>Gender Matters: Internal Medicine Resident Perceptions of Gender Bias in Medical Training<br><br>Rogers, Elizabeth A.; Moser-Bleil, E. Kendahl; Duffy, Briar L.; Gladding, Sophia; Wang, Qi; Mustapha, Taj<br><br>Journal of General Internal Medicine 2021;36(5):1448-1450<br><br>United States 2021<br><br>DOI: 10.1007/s11606-020-05772-8<br><br>Upload full text<br>1 Note History Duplicate Move to Full text	09/03/23	Wrong outcomes	
☐	#287 - Graham 2021<br>Gender-based Barriers in the Advancement of Women Leaders in Emergency Medicine: A Multi-institutional Qualitative Study<br><br>Graham, Emily M.; Ferrel, Meganne N.; Wells, Katie M.; Egan, Daniel J.; MacVane, Casey Z.; Gisondi, Michael A.; Burns, Boyd D.; Madsen, Troy E.; Fix, Megan L.<br><br>Western Journal of Emergency Medicine: Integrating Emergency Care with Population Health 2021;22(6):1355-1359<br><br>Orange, California Western Journal of Emergency Medicine: Integrating Emergency Care with Population Health 2021<br><br>DOI: 10.5811/westjem.2021.7.52826<br><br>Full text Abstract<br>• qt53c707nt.pdf Primary<br>• Full text uploaded by Covidence (Open Access)<br>• Manage full text<br><br>1 Note History Duplicate Move to Full text	09/03/23	Wrong patient population	

	Excluded On	Reason
#98 - Jenner 2022<br>Prevention Strategies for Sexual Harassment in Academic Medicine: A Qualitative Study<br><br>Jenner, Sabine C.; Djermester, Pia; Oertelt-Prigione, Sabine<br><br>Journal of interpersonal violence 2022;37(5-6):NP2490-NP2515<br><br>United States 2022<br><br>DOI: 10.1177/0886260520903130<br><br>Upload full text Abstract<br>1 Note History Duplicate Move to Full text	09/03/23	Wrong patient population
#238 - Malik 2021<br>Workplace mistreatment and mental health in female surgeons in Pakistan<br><br>Malik, M. A.; Inam, H.; Martins, R. S.; Janjua, M. B. N.; Zahid, N.; Khan, S.; Sattar, A. K.; Khan, S.; Haider, A. H.; Enam, S. A.<br><br>Bjs Open 2021;5(3):<br><br>England 2021<br><br>DOI: 10.1093/bjsopen/zrab041<br><br>Full text Abstract<br><br>• zrab041.pdf<br>Primary<br>• Full text uploaded by Covidence (Open Access)<br>• Manage full text<br><br>1 Note History Duplicate Move to Full text	09/03/23	Wrong patient population
#233 - Moshref 2021<br>Perception, Academic Performance, Gender Judgment and Barriers among Surgeons' Career Progression in Jeddah, Saudi Arabia: A Cross-Sectional Study<br><br>Moshref, Rana; Moshref, Leena; Rizk, Hisham; Fayez, Raad; Alotaibi, Abdulrahman<br><br>Annals of medicine and surgery (2012) 2021;61():48-53<br><br>England 2021<br><br>DOI: 10.1016/j.amsu.2020.12.009<br><br>Upload full text Abstract<br>1 Note History Duplicate Move to Full text	09/03/23	Wrong outcomes

	Excluded On	Reason
#331 - Bansal 2021<br>Women in Dermatology Leadership: Results from a Nationwide Survey<br><br>Bansal, Anuva; Sarkar, Rashmi<br><br>Indian dermatology online journal 2021;12(6):834-840<br><br>India 2021<br><br>DOI: 10.4103/idoj.idoj_279_21<br><br>Upload full text Abstract<br>Note History Duplicate Move to Full text	09/03/23	Wrong outcomes
#128 - Dellasega 2022<br>The Impact of Patient Prejudice on Minoritized Female Physicians<br><br>Dellasega, Cheryl; Aruma, Jane-Frances; Sood, Natasha; Andreae, Doerthe A.<br><br>Frontiers in Public Health 2022;10():902294<br><br>Switzerland 2022<br><br>DOI: 10.3389/fpubh.2022.902294<br><br>Full text Abstract<br><br>• pdf.pdf<br>Primary<br>• Full text uploaded by Covidence (Open Access)<br>• Manage full text<br><br>1 Note History Duplicate Move to Full text	09/03/23	Wrong patient population
#81 - Maso 2022<br>Qualitative description of sexual harassment and discrimination of women in emergency medicine: Giving the numbers a voice<br><br>Maso, Kristi; Theobald, Jillian L.<br><br>AEM education and training 2022;6(2):e10727<br><br>United States 2022<br><br>DOI: 10.1002/aet2.10727<br><br>Upload full text Abstract<br>1 Note History Duplicate Move to Full text	09/03/23	Wrong patient population
#76 - Methangkool 2022	09/03/23	Wrong patient

	Excluded On	Reason
Perceptions of Gender Disparities Among Women in Cardiothoracic Anesthesiology<br><br>Methangkool, Emily; Brodt, Jessica; Kolarczyk, Lavinia; Ivascu, Natalia S.; Hicks, Megan H.; Herrera, Elizabeth; Oakes, Daryl<br><br>Journal of cardiothoracic and vascular anesthesia 2022;36(7):1859-1866<br><br>United States 2022<br><br>DOI: 10.1053/j.jvca.2021.11.015<br><br>Upload full text Abstract<br>1 Note History Duplicate Move to Full text		population
#174 - WaryamSinghMalhi 2021<br>Self-perception of sexual harassment: A comparison between female medical and nursing students during clinical practice<br><br>Waryam Singh Malhi, Fatehpal Singh A. L.; Sugathan, Sandheep; Binti Azhar, Nik Ameera Syafiqa; Binti Wan Roslan, Wan Imanah Nabilah; Abu Bakar, Hanan Asrawi Binti; Binti Zolkaine, Siti Maisarah<br><br>Education for health (Abingdon, England) 2021;34(2):55-63<br><br>India 2021<br><br>DOI: 10.4103/1357-6283.332958<br><br>Upload full text Abstract<br>1 Note History Duplicate Move to Full text	09/03/23	Wrong patient population
#162 - Adar 2022<br>Gender bias in the evaluation of interns in different medical specialties: An archival study<br><br>Adar, Roy; Kahalon, Rotem; Ullrich, Johannes; Afek, Arnon; Eisenberg, Vered H.<br><br>Medical Teacher 2022;44(8):893-899<br><br>England 2022<br><br>DOI: 10.1080/0142159X.2022.2046715<br><br>Upload full text Abstract<br>Note History Duplicate Move to Full text	09/03/23	Wrong outcomes
#69 - Olsen 2022<br>Understanding the Effect of Bias on the Experience of Women Surgeons: A Qualitative Study	09/03/23	Wrong outcomes

		Excluded On	Reason
	Olsen, Bridget C.; Barron, Sivana L.; Gutheil, Caitlin M.; Blazick, Elizabeth A.; Mayo, Sara W.; Turner, Elizabeth N.; Whiting, James F.<br><br>Journal of the American College of Surgeons 2022;234(6):1064-1072<br><br>United States 2022<br><br>DOI: 10.1097/XCS.0000000000000162<br><br>Upload full text Abstract<br>1 Note History Duplicate Move to Full text		
☐	#75 - Najafzadeh 2022<br>The impact of work schedules, workplace bullying and some demographic characteristics on nurses' sleep quality in Iran<br><br>Najafzadeh, Masoomeh; Amini, Kourosh; Sadeghniiat-Haghighi, Khosro; Kamali, Koorosh<br><br>Sleep science (Sao Paulo, Brazil) 2022;15(1):62-67<br><br>Brazil 2022<br><br>DOI: 10.5935/1984-0063.20210029<br><br>Full text Abstract<br><br>• v15n1a09.pdf<br>Primary<br>• Full text uploaded by Covidence (Open Access)<br>• Manage full text<br><br>Note History Duplicate Move to Full text	09/03/23	Wrong outcomes
☐	#215 - Papantoniou 2021<br>Sexual harassment and organisational silencing in nursing: a cross-sectional study in Greece<br><br>Papantoniou, Panagiotis<br><br>Bmj Open 2021;11(11):e050850<br><br>England 2021<br><br>DOI: 10.1136/bmjopen-2021-050850<br><br>Upload full text Abstract<br>1 Note History Duplicate Move to Full text	09/03/23	Wrong patient population
☐	#106 - Hiemstra 2022<br>Experiences of Canadian Female Orthopaedic Surgeons in the Workplace: Defining the Barriers to Gender Equity	09/03/23	Wrong patient population

	Excluded On	Reason
Hiemstra, Laurie A.; Kerslake, Sarah; Clark, Marcia; Temple-Oberle, Claire; Boynton, Erin<br><br>The Journal of bone and joint surgery. American volume 2022;104(16):1455-1461<br><br>United States 2022<br><br>DOI: 10.2106/JBJS.21.01462<br><br>Upload full text Abstract<br>1 Note History Duplicate Move to Full text		
☐ #146 - Boles 2022<br>Nurse, Provider, and Emergency Department Technician: Perceptions and Experiences of Violence and Aggression in the Emergency Department<br><br>Boles, Jean M.; Maccarone, Diane; Brown, Beverly; Archer, Alexandra; Trotter, Michael G.; Friedman, Nicholas M. G.; Chittams, Jesse; Mazzone, Leighann; Ballinghoff, James; Burchill, Christian N.; Cacchione, Pamela Z.<br><br>Journal of Emergency Nursing 2022;():<br><br>United States 2022<br><br>DOI: 10.1016/j.jen.2022.07.008<br><br>Upload full text Abstract<br>1 Note History Duplicate Move to Full text	09/03/23	Wrong outcomes
☐ #236 - Martins 2021<br>Harassment and mental health in surgical training: a pilot survey of surgical trainees in Pakistan<br><br>Martins, Russell Seth; Hashmi, Syeda Amrah; Inam, Hina; Naeem Janjua, Mahin Binte; Malik, Mahim Akmal<br><br>JPMA. The Journal of the Pakistan Medical Association 2021;71(Suppl 1)(1):S23-S28<br><br>Pakistan 2021<br><br>Upload full text Abstract<br>Note History Duplicate Move to Full text	09/03/23	present in previous search
☐ #315 - Chen 2021<br>A Report of Gender Bias and Sexual Harassment in Current Plastic Surgery Training: A National Survey<br><br>Chen, Wendy; Schilling, Benjamin K.; Bourne, Debra A.; Myers, Sara; De La Cruz, Carolyn<br><br>Plastic and Reconstructive Surgery 2021;147(6):1454-1468	09/03/23	present in previous search

		Excluded On	Reason
	United States 2021<br><br>DOI: 10.1097/PRS.0000000000007994<br>Upload full text Abstract<br>Note History Duplicate Move to Full text		
☐	#153 - Ayhan 2022<br>The aggressive patient experiences of healthcare professionals exposed to physical violence in a psychiatric clinic: A phenomenological study<br><br>Ayhan, Didem; Mercan, Neşe; Doğan, Reyhan; Yüksel, Çiğdem<br><br>Perspectives in psychiatric care 2022;58(2):501-508<br><br>Malden, Massachusetts Wiley-Blackwell 2022<br><br>DOI: 10.1111/ppc.12918<br><br>Upload full text Abstract<br>Note History Duplicate Move to Full text	09/03/23	Wrong patient population
☐	#318 - Cartron 2021<br>A survey study of dermatologists' experiences of sexual harassment<br><br>Cartron, Alexander M.; Shah, Payal; Roman, Jorge; Zampella, John<br><br>International Journal of Women's Dermatology 2021;7(3):342-343<br><br>United States 2021<br><br>DOI: 10.1016/j.ijwd.2020.10.009<br><br>Upload full text<br>Note History Duplicate Move to Full text	09/03/23	an editorial
☐	#191 - Silva 2021<br>Gender violence against woman nursing students: a cross-sectional study<br><br>Silva, Luiza Csordas Peixinho da; Hino, Paula; Oliveira, Rebeca Nunes Guedes de; Fernandes, Hugo<br><br>Revista Brasileira De Enfermagem 2021;74(5):e20200539<br><br>Brazil 2021<br><br>DOI: 10.1590/0034-7167-2020-0539<br><br>Upload full text Abstract<br>1 Note History Duplicate Move to Full text	09/03/23	Wrong patient population

	Excluded On	Reason
#15 - Weldesenbet 2022<br>Sexual Harassment and Associated Factors Among Female Nurses: The Case of Addis Ababa Public Hospitals<br><br>Weldesenbet, Habtamu; Yibeltie, Jemberu; Hagos, Tsega<br><br>Psychology Research and Behavior Management 2022;15():3053-3068<br><br>New Zealand 2022<br><br>DOI: 10.2147/PRBM.S372422<br><br>Full text Abstract<br><br>• getfile.php.pdf<br>Primary<br>• Full text uploaded by Covidence (Open Access)<br>• Manage full text<br><br>1 Note History Duplicate Move to Full text	09/03/23	Wrong patient population
#197 - Santos 2021<br>PERCEPTION OF HARASSMENT AMONG FEMALE SURGEONS<br><br>Santos, Elizabeth G.; Roque, Lia; Maya, Maria Cristina; Moreira, Reni Cecilia; Lima, Fernanda Lage; Correia, M. Isabel T. D.<br><br>Revista do Colegio Brasileiro de Cirurgioes 2021;48():e20213123<br><br>Brazil 2021<br><br>DOI: 10.1590/0100-6991e-20213123<br><br>Upload full text Abstract<br>1 Note History Duplicate Move to Full text	09/03/23	Wrong patient population
#340 - Alshahrani 2021<br>Incidence and prevalence of violence toward health care workers in emergency departments: a multicenter cross-sectional survey<br><br>Alshahrani, Mohammed; Alfaisal, Razan; Alshahrani, Khalid; Alotaibi, Leyan; Alghoraibi, Hissah; Alghamdi, Eman; Almusallam, Lulwah; Saffarini, Zaineh; Alessa, Salihah; Alwayel, Faten; Saffarini, Lubna; Alrawdhan, Ali; Mapusao, Charlene; Asonto, Laila Perlas; Alsulaibikh, Amal; Aljumaan, Mohammed<br><br>International Journal of Emergency Medicine 2021;14(1):1-8<br><br>, <Blank> Springer Nature 2021<br><br>DOI: 10.1186/s12245-021-00394-1<br><br>Full text Abstract	09/03/23	Wrong outcomes

	Excluded On	Reason
• s12245-021-00394-1.pdf<br>Primary<br>• Full text uploaded by Covidence (Open Access)<br>• Manage full text<br>1 Note History Duplicate Move to Full text		
#102 - Iyer 2022<br>Why Gender-Based Bullying Is Normalized in Academic Medicine: Experiences and Perspectives of Women Physician Leaders<br><br>Iyer, Maya S.; Way, David P.; MacDowell, Doug J.; Overholser, Barbara; Spector, Nancy D.; Jagsi, Reshma<br><br>Journal of women's health (2002) 2022;():<br><br>United States 2022<br><br>DOI: 10.1089/jwh.2022.0290<br><br>Upload full text Abstract<br>1 Note History Duplicate Move to Full text	09/03/23	Wrong patient population
#91 - Kim 2022<br>Relationships between Violence Experience, Resilience, and the Nursing Performance of Emergency Room Nurses in South Korea<br><br>Kim, Sarang; Gu, Minkyung; Sok, Sohyune<br><br>International Journal of Environmental Research and Public Health 2022;19(5):<br><br>Switzerland 2022<br><br>DOI: 10.3390/ijerph19052617<br><br>Full text Abstract<br>• pdf.pdf<br>Primary<br>• Full text uploaded by Covidence (Open Access)<br>• Manage full text<br><br>1 Note History Duplicate Move to Full text	09/03/23	Wrong outcomes
#133 - Chowdhury 2022<br>Workplace violence, bullying, burnout, job satisfaction and their correlation with depression among Bangladeshi nurses: A cross-sectional survey during the COVID-19 pandemic<br><br>Chowdhury, Saifur Rahman; Kabir, Humayun; Mazumder, Sinthia; Akter, Nahida; Chowdhury, Mahmudur Rahman; Hossain, Ahmed	09/03/23	Wrong outcomes

	Excluded On	Reason
Plos One 2022;17(9):e0274965<br><br>United States 2022<br><br>DOI: 10.1371/journal.pone.0274965<br>Full text Abstract<br><br>• file.pdf<br>Primary<br>• Full text uploaded by Covidence (Open Access)<br>• Manage full text<br>1 Note History Duplicate Move to Full text		
#216 - Papantoniou 2021<br>Are male nurses sexually harassed? A cross-sectional study in the Greek Health System<br><br>Papantoniou, Panagiotis<br><br>Bmc Nursing 2021;20(1):137<br><br>England 2021<br><br>DOI: 10.1186/s12912-021-00656-6<br><br>Full text Abstract<br><br>• s12912-021-00656-6.pdf<br>Primary<br>• Full text uploaded by Covidence (Open Access)<br>• Manage full text<br><br>1 Note History Duplicate Move to Full text	09/03/23	Wrong patient population
#49 - Pitot 2022<br>You Too? Female Radiologists' Narratives on Discrimination and Harassment<br><br>Pitot, Marika A.; White, Marney A.; Edney, Elizabeth; Mogensen, Monique A.; Solberg, Agnieszka; Kattapuram, Taj; Sinha, Cynthia; Kadom, Nadja<br><br>Journal of the American College of Radiology : JACR 2022;19(2 Pt A):288-303<br><br>United States 2022<br><br>DOI: 10.1016/j.jacr.2021.09.043<br><br>Upload full text Abstract<br>1 Note History Duplicate Move to Full text	09/03/23	Wrong patient population
#9 - ZareKhafri 2022<br>Nurses' perception of workplace discrimination	09/03/23	Wrong outcomes

		Excluded On	Reason
	ZareKhafri, Fatemeh; Torabizadeh, Camellia; Jaberi, Azita<br><br>Nursing Ethics 2022;29(3):675-684<br><br>England 2022<br><br>DOI: 10.1177/09697330211015291<br><br>Upload full text Abstract<br>1 Note History Duplicate Move to Full text		
☐	#221 - Ouyang 2021<br>Persistence of Gender Bias Over Four Decades of Surgical Training<br><br>Ouyang, Kelsey; Huang, Ivy A.; Wagner, Justin P.; Wu, James; Chen, Formosa; Quach, Chi; Donahue, Timothy R.; Hines, O. Joe; Hiatt, Jonathan R.; Tillou, Areti<br><br>Journal of surgical education 2021;78(6):1868-1877<br><br>United States 2021<br><br>DOI: 10.1016/j.jsurg.2021.06.008<br><br>Upload full text Abstract<br>1 Note History Duplicate Move to Full text	09/03/23	Wrong patient population
☐	#127 - Dill 2022<br>Structural Racism And Black Women's Employment In The US Health Care Sector<br><br>Dill, Janette; Duffy, Mignon<br><br>Health affairs (Project Hope) 2022;41(2):265-272<br><br>United States 2022<br><br>DOI: 10.1377/hlthaff.2021.01400<br><br>Upload full text Abstract<br>1 Note History Duplicate Move to Full text	09/03/23	Wrong patient population
☐	#97 - Jeong 2022<br>Workplace sexual harassment toward male nurses in South Korea: a cross-sectional online survey<br><br>Jeong, Suyong; Chang, Hyoung Eun<br><br>Bmc Nursing 2022;21(1):303<br><br>England 2022<br><br>DOI: 10.1186/s12912-022-01091-x	09/03/23	Wrong patient population

	Excluded On	Reason
Full text Abstract<br>• s12912-022-01091-x.pdf Primary<br>• Full text uploaded by Covidence (Open Access)<br>• Manage full text<br>1 Note History Duplicate Move to Full text		
#101 - Iyer 2022<br>Bullying in Academic Medicine: Experiences of Women Physician Leaders<br><br>Iyer, Maya S.; Way, David P.; MacDowell, Doug J.; Overholser, Barbara M.; Spector, Nancy D.; Jagsi, Reshma<br><br>Academic medicine : journal of the Association of American Medical Colleges 2022;():<br><br>United States 2022<br><br>DOI: 10.1097/ACM.0000000000005003<br><br>Upload full text Abstract<br>1 Note History Duplicate Move to Full text	09/03/23	Wrong patient population
#308 - Crown 2021<br>The Role of Race and Gender in the Career Experiences of Black/African American Academic Surgeons: A Survey of the Society of Black Academic Surgeons and a Call to Action<br><br>Crown, Angelena; Berry, Cherisse; Khabele, Dineo; Fayanju, Oluwadamilola M.; Cobb, Adrienne; Backhus, Leah; Smith, Randi N.; Sweeting, Raeshell; Hasson, Rian M.; Johnson-Mann, Crystal; Oseni, Tawakalitu; Newman, Erika A.; Turner, Patricia; Karpeh, Martin; Pugh, Carla; Jordan, Andrea Hayes; Henry-Tillman, Ronda; Joseph, Kathie-Ann<br><br>Annals of surgery 2021;273(5):827-831<br><br>United States 2021<br><br>DOI: 10.1097/SLA.0000000000004502<br><br>Upload full text Abstract<br>1 Note History Duplicate Move to Full text	09/03/23	Wrong outcomes
#96 - Jia 2022<br>Prevalence, characteristics, and consequences of verbal and physical violence against healthcare staff in Chinese hospitals during 2010-2020<br><br>Jia, Chen; Han, Yijing; Lu, Wenping; Li, Ruofan; Liu, Weizheng; Jiang, Jianan<br><br>Journal of occupational health 2022;64(1):e12341	09/03/23	Wrong outcomes

		Excluded On	Reason
	Australia 2022<br><br>DOI: 10.1002/1348-9585.12341<br>Upload full text Abstract<br>1 Note History Duplicate Move to Full text		
☐	#60 - Pandit 2022<br>Challenges and gender-based differences for women in the Indian urological workforce: Results of a survey<br><br>Pandit, Shruti; Venugopal, P.; Keshavamurthy, Ramaiah; Chawla, Arun<br><br>Indian Journal of Urology 2022;38(4):282-286<br><br>Wolters Kluwer India Pvt Ltd 2022<br><br>DOI: 10.4103/iju.iju_143_22<br><br>Upload full text Abstract<br>1 Note History Duplicate Move to Full text	09/03/23	Wrong patient population

- © 2024 Covidence
- Terms
- Privacy

Feedback & Support

Find your answer, fast.

We've got a new Knowledge base that answers many frequently asked questions. Why not try that first?

View the knowledge base OR Send us an email
