# Supplementary material for: Perpetrators of gender-based workplace violence amongst nurses and physicians–A scoping review of the literature
Source: PLOS Glob Public Health. 2024 Sep 6;4(9):e0003646. doi: 10.1371/journal.pgph.0003646 (PMC11379169; doi:10.1371/journal.pgph.0003646)
Supplement: Attachment — Submitted filename: PGPH-D-24-01206_reviewer_SS.pdf [file pgph.0003646.s007.pdf]

PLOS Global Public Health

Perpetrators of Gender-based Workplace Violence Amongst Nurses and Physicians – A Scoping Review of the Literature

--Manuscript Draft--

Manuscript Number:	PGPH-D-24-01206
Article Type:	Research Article
Full Title:	Perpetrators of Gender-based Workplace Violence Amongst Nurses and Physicians – A Scoping Review of the Literature
Short Title:	Perpetrators for Gender-based Workplace Violence
Corresponding Author:	Basnama Ayaz, BScN, MScN, PhD (c)<br>University of Toronto Lawrence S Bloomberg Faculty of Nursing<br>Toronto, Ontario CANADA
Order of Authors:	Basnama Ayaz, BScN, MScN, PhD (c)
	Graham Dozois, RN, BSc, MN
	Andrea L Baumann, RN, MN, PhD (c)
	Adam Fuseini, RN, BSc, MN
	Sioban Nelson, R.N., PhD, FCAHS, FCAN, FAAN
Keywords:	Gender, workplace violence, perpetrator, health, workforce, nurses, physicians
Abstract:	In healthcare settings worldwide, workplace violence (WPV) has been extensively studied. However, significantly less is known about gender-based WPV and the characteristics of perpetrators. We conducted a comprehensive scoping review on Type II (directed by consumers) and Type III (perpetuated by healthcare workers) gender based-WPV among nurses and physicians globally. For the review, we followed the Preferred Reporting Items for Systematic and Meta Analyses extension for Scoping Review (PRISMA-ScR). The protocol for the comprehensive review was registered on the Open Science Framework on January 14, 2022, at https://osf.io/t4pfb/. A systematic search in five health and social science databases yielded 178 relevant studies that indicated types of perpetrators, with only 34 providing descriptive data for perpetrators' gender. Across both types of WPV, men (65.1%) were more frequently responsible for perpetuating WPV compared to women (28.2%) and both genders (6.7%). Type II WPV, demonstrated a higher incidence of violence against women; linked to the gendered roles, stereotypes, and societal expectations that allocate specific responsibilities based on gender. Type III WPV was further categorized into Type III-A (horizontal) and Type III-B (vertical). With Type III WPV, gendered power structures and stereotypes contributed to a permissive environment for violence by men that victimized women. These revelations emphasize the pressing need for gender-sensitive strategies for addressing WPV within the healthcare sector. Policymakers must prioritize the security of healthcare workers, especially women, through reforms and zero-tolerance policies. Promoting gender equality and empowerment within the workforce and leadership is pivotal. Additionally, creating a culture of inclusivity, support, and respect, led by senior leadership, acknowledging WPV as a structural issue and enabling an open dialogue across all levels are essential for combating this pervasive problem.
Additional Information:	
Question	Response
Financial Disclosure<br><br>Enter a financial disclosure statement that describes the sources of funding for the	The authors received no financial support for this article's research, authorship and/or publication.

Powered by Editorial Manager® and ProduXion Manager® from Aries Systems Corporation

work included in this submission and the role the funder(s) played. This includes grants and any commercial funding of the work or authors.<br><br>This statement will be typeset if the manuscript is accepted for publication.<br><br>*Please review the submission guidelines and the instructions link below for detailed requirements and guidance.*	
**Competing Interests**<br><br>On behalf of all authors, disclose any competing interests that could be perceived to bias this work.<br><br>This statement will be typeset if the manuscript is accepted for publication.<br><br>*Please review the instructions link below and PLOS Global Public Health's competing interests policy to determine what information must be disclosed at submission.*	The authors declared no potential conflicts of interest with respect to the research, authorship, and/or publication of this article.
**Data Availability**<br><br>Before publication, Authors are required to make fully available and without restriction all data underlying their findings. Please see our PLOS Data Policy page for detailed information on this policy.<br><br>A **Data Availability Statement**, detailing where the data can be accessed, is required at first submission. Insert your Data Availability Statement in the box below.<br><br>Please see the data reporting section of our submission guidelines for instructions	The manuscript is a scoping review of workplace violence among healthcare professionals based on gender. All the abstracted data pertinent to the manuscript (178 studies) is tabulated in Table 1. All the data is publicly available online.

on what you need to include in your Data Availability Statement.<br><br>This statement will be typeset if the manuscript is accepted for publication.<br><br>*PLOS allows rare exemptions to address legal and ethical concerns. If you have legal or ethical restrictions, please use the box below to detail these in full sentences for the Journal team to consider.*	

Manuscript

Click here to access/download;Manuscript;Manuscript On GBV-Perpetrators.docx

Title Page

Title: Perpetrators of Gender-based Workplace Violence Amongst Nurses and Physicians – A Scoping Review of the Literature

Basnama Ayaz[1]* Graham Dozois[2] Andrea L. Baumann[1], Adam Fuseini[1], Sioban Nelson[1]

[1]Lawrence S. Bloomberg Faculty of Nursing, University of Toronto, Toronto, ON, Canada.

[2]Princess Margaret Cancer Centre, Toronto, Canada

*Corresponding Author
E-mail: basnama.ayaz@mail.utoronto.ca

Perpetrators of Gender-based Workplace Violence Amongst Nurses and Physicians – A Scoping Review of the Literature

Abstract

In healthcare settings worldwide, workplace violence (WPV) has been extensively studied. However, significantly less is known about gender-based WPV and the characteristics of perpetrators. We conducted a comprehensive scoping review on Type II (directed by consumers) and Type III (perpetuated by healthcare workers) gender based-WPV among nurses and physicians globally. For the review, we followed the Preferred Reporting Items for Systematic and Meta Analyses extension for Scoping Review (PRISMA-ScR). The protocol for the comprehensive review was registered on the Open Science Framework on January 14, 2022, at https://osf.io/t4pfb/. A systematic search in five health and social science databases yielded 178 relevant studies that indicated types of perpetrators, with only 34 providing descriptive data for perpetrators' gender. Across both types of WPV, men (65.1%) were more frequently responsible for perpetuating WPV compared to women (28.2%) and both genders (6.7%). Type II WPV, demonstrated a higher incidence of violence against women; linked to the gendered roles, stereotypes, and societal expectations that allocate specific responsibilities based on gender. Type III WPV was further categorized into Type III-A (horizontal) and Type III-B (vertical). With Type III WPV, gendered power structures and stereotypes contributed to a permissive environment for violence by men that victimized women. These revelations emphasize the pressing need for gender-sensitive strategies for addressing WPV within the healthcare sector. Policymakers must prioritize the security of healthcare workers, especially women, through reforms and zero-tolerance policies. Promoting gender equality and empowerment within the workforce and leadership is pivotal. Additionally, creating a culture of inclusivity, support, and respect, led by senior

leadership, acknowledging WPV as a structural issue and enabling an open dialogue across all levels are essential for combating this pervasive problem.

Keywords: Gender, workplace violence, perpetrator, health, workforce, nurses, physicians

Introduction

The International Labour Office (ILO), the International Council of Nurses (ICN), the World Health Organization (WHO), and Public Services International (PSI) defined WPV as "incidents where staff are abused, threatened or assaulted in circumstances related to their work, including commuting to and from work, involving an explicit or implicit challenge to their safety, well-being or health" (1, p.3). The ILO (2) further defined gender-based violence as violence directed towards individuals or groups based on gender, encompassing violence against both women and men.

Irrespective of industry, workplace violence (WPV) can cause lasting trauma and injuries and is a serious threat to human and health resources. WPV includes physical and psychological violence, including physical assault, verbal abuse, sexual harassment, and bullying. Gender-based workplace violence (GB-WPV), which is experienced across operational layers of an organization (horizontal) and organizational hierarchy (vertical), reinforces the differential risk for exposure and outcomes of violence for men and women (2). Despite extensive research on workplace violence, GB-WPV, its perpetrators, and its impact on healthcare professionals remains understudied. This paper, reports on GB-WPV perpetrators.

A systematic review (3) of the consequences of exposure to WPV in the healthcare setting based on 68 studies reported psychological and emotional effects such as post-traumatic stress, depression, anger, and fear. These effects impact work productivity, leading to increased sick leaves, poor job satisfaction, burnout, and higher attrition rates, particularly for women (3). Studies have also shown that men are more likely to commit physical violence (4) and sexual harassment (5,6), while women are more often engaged in verbal abuse (7). Moreover, gender stereotypes and inequalities in the distribution of roles and responsibilities can worsen power imbalances (2). By recognizing and understanding these

issues, employers and organizations can more effectively prevent and deal with gender-based workplace violence, ensuring a safer and more equitable work environment for everyone.

The classification of workplace violence has evolved, delineating distinct categories shaped by the nature of its perpetrators. The current working taxonomy categorizes WPV into four types based on the perpetrators of violence. This typology, as shown in Table 1, emerged from a collaborative effort of a workshop on workplace violence intervention research held in Washington, DC, in 2000. The findings of this endeavour were subsequently published by the U.S. Department of Justice in 2001 (11). Since then, this framework has been adopted by multiple organizations (8,9,12), and by researchers (10,13).

Table 1: Type of Violence based on Perpetrators

**Type I**	Workplace violence committed by a person who has no legitimate business at the work site" with criminal intent.
**Type II**	Workplace violence directed at employees by customers/service consumers, including clients, patients, students, inmates, or visitors, who are patients' companions.
**Type III**	Workplace violence against an employee by a present or former employee, supervisor, or manager (8).
	**Horizontal or lateral violence** is “violence, harassment or bullying directed at colleagues who are at equal level within an organization” (9).
	**Vertical violence** perpetrated by senior colleagues, supervisors, and administrative personnel at a higher level than the victim in the organizational or professional hierarchy (9,10)
**Type IV**	Workplace violence is committed by someone who is not an employee but has or is known to have had a personal relationship with an employee (8).

This paper explores the dynamics of workplace violence by categorizing and summarizing both Type II WPV, from patients and significant others, and Type III WPV (horizontal and vertical), which pertain to violence perpetuated by colleagues, supervisors, and administrators within the organization. Additionally, we explore the nuances of GB-WPV, considering both the perpetrators and nurses and physicians as victims of WPV. Understanding the factors contributing to these types of WPV is crucial to developing effective preventive interventions and strategies. Currently, there is a dearth of information identifying the characteristics of individuals who are more likely to commit GB-WPV and the characteristics of those targeted by such offences. This review addresses this gap by

synthesizing data from studies that reported on the gender/sex of perpetrators for WPV among nurses and physicians.

While WPV affects individuals across the gender spectrum and in different professional groups, women are disproportionately affected. This is attributed to their preponderance in the health workforce, their overrepresentation in lower positions in organizational and professional hierarchies, and societal gender norms that subjugate women (7,14). Recognizing that workplace violence is fundamentally intertwined with broader societal structures rooted in socioeconomic, cultural, and institutional factors, we underscore the necessity of a systematic approach to address this issue—one that is integrated, participatory, culturally and gender-sensitive, and non-discriminatory (1). While current interventions aimed at addressing WPV primarily focus on assessing the effectiveness of training interventions to prevent and manage WPV in healthcare settings (15–17), they often lack gender-segregated findings for their effectiveness. Clarifying the existing situation on the gender of victims and perpetrators for specific Type/s of violence would help develop gender-sensitive interventions and policies to more effectively address WPV. This scoping review focuses on understanding GB-WPV and its perpetrators in the global health workforce, including nurses and physicians. Our preliminary search for a scoping review revealed that GB-WPV affects men, women, and non-binary persons. However, most studies included in this review considered gender as binary (men and women); only a few studies included non-binary personnel (for sample-see Table 2, in results section). Therefore, we defined *gender* as a binary for this review.

Our specific objectives set out for this paper are:

1. Describe the proportions of WPV and related perpetrator/s contributing to Type II (from patients/clients/families) and Type III (worker-to-worker) violence among nurses and physicians in different contexts.

2. Summarize the gendered perpetration of Type II and Type III WPV against men and women in the health workforce.
3. Identify gaps in the state of knowledge to recommend direction for future empirical research studies.

Methods

Protocol Registration and Study Design

Following the Joanna Briggs Institute (JBI) revised guidelines, we conducted a scoping review. The protocol for this review was registered on the Open Science Framework on January 14, 2022, and is accessible at https://osf.io/t4pfb/. The scoping literature review design addressed the research questions and accommodated the heterogeneous and complex nature of the literature. This method is appropriate for exploring the extent of the literature, mapping and summarizing the evidence, and identifying and analyzing knowledge gaps to inform future research. The framework used for this review consists of eight steps; they are built upon the seminal framework of Arksey and O'Malley's scoping review, which was further developed by Levac and colleagues. The revised guidelines of JBI align these eight steps with the Preferred Reporting Items for Systematic and Meta-Analyses extension for Scoping Reviews (PRISMA-ScR), ensuring rigour, transparency, and trustworthiness in Reporting the conduct of the scoping review. The first step of the scoping review framework is to align with research objectives, the title, and the inclusion criteria, as well as the exclusion criteria (see Box 1).

Box 1: Study Selection Criteria

Inclusion Criteria for Studies
1. The study participants included nurses and/ or physicians who experienced WPV during their careers.
2. Provided sex-segregated data for any form of violence and any type of perpetrators among nurses and physicians, including students, globally.
3. Published in English and after 2010.

Exclusion criteria
4. Studies that did not provide sex-segregated data and information for perpetrators.
5. Exclude systematic/ scoping reviews, concept or theoretical papers, and theses.

Search Strategy

The research team collaborated with a health sciences librarian to develop a comprehensive search strategy. The

systematic search focused on published literature in various databases, including Ovid MEDLINE: Epub Ahead of Print, In-Process and Other Non-Indexed Citations, which were translated in CINAHL Plus, APA PsycINFO, Web of Sciences, and Gender Studies Databases, Applied Social Sciences Index & Abstracts (ASSIA), and Sociological Abstracts (The OVID MEDLINE search strategy is presented in Appendix A, was translated in all other databases). The search terms related to the population (midwifery, nursing, and physicians), concepts (violence and gender-based violence), and context (healthcare) were combined appropriately based on the scoping review objectives. These terms were identified through a preliminary literature search on various aspects of workplace violence in Google Scholar. The final search results were exported to EndNote, a citation manager, to de-duplicate sources from multiple databases. After de-duplication, the sources were imported into the Covidence online software program that streamlined the screening process by two independent reviewers. The final search of the literature review for this study was conducted on 11th February 2024.

Evidence Screening and Selection

The identified sources were screened based on the inclusion criteria. Two independent reviewers screened the titles and abstracts to shortlist the sources. Discrepancies were resolved through discussion and consensus, with a complete source review conducted, if necessary, followed by a full-text review against the inclusion criteria by two reviewers. The selection process is presented in the PRISMA diagram (Figure 1). Given the overall objective of the review to map the most frequent forms and prevalence of GB-WPV for midwives, nurses, and physicians in different contexts and clinical settings, a quality assessment of the identified sources was not conducted.

Figure 1: PRISMA Flow Diagram for Screening and Selection

Data Analysis and Synthesis of Results

This paper is a component of a multi-part scoping review; it reports on the perpetrators of WPV from gender-segregated prevalence data reported from a global context among the health workforce, including nurses and physicians. The prevalence and risk factors have been reported elsewhere. This paper reports on Type II and Type III (vertical and horizontal) WPV perpetrators. Data from all sources that reported sex/gender segregated findings and provided information for the types of perpetrators were included in mapping the prevalence of GB-WPV (See Table 2) for several types/forms of WPV and the clinical setting across countries/special regions. We could not calculate a mean score for various forms of violence (presented in Appendix B) based on gender for all studies that provided information on perpetrators because of wide variability in the definition of the terms and the concepts. Only 34 studies (19%) provided the gender of perpetrators. We summarized the proportion of male and female perpetrators in those studies for Type II, Type III-A (horizontal) and Type III-B (Vertical) violence (see Table 3).

Results

After de-duplication, 8435 possible references were imported for screening in the Covidence. These studies were screened against the title by one person, 1551 were shortlisted to be screened (for title and abstract) by two independent reviewers, and 402 were assessed for full-text eligibility. After applying the inclusion and exclusion criteria, 178 (4–7,13,18–190) studies were retained (PRISMA diagram 1) and analyzed to report on perpetrators that provided gender-segregated findings for WPV and information on various types of perpetrators (Table 2). We included studies published between 2010-2024. The most common study design was quantitative, cross-sectional (n = 168), mixed methods (n = 4), and qualitative methods (n=6).

Table 2: Prevalence of Various Types/Forms of Workplace Violence by Gender and Types of Perpetuators in Different Clinical Settings and Professional Categories Across World

S. #	***Author/s, year***	***Clinical Setting***	***Professional Category/ies***	***Sample (Female; Male)***	***Country/ies***	***Prevalence of WPV by Type* (Female; *Male)***	***Perpetrators***		
							Type 2	***Type 3A***	***Type 3B***
1.	Aghajanloo et al., 2011 (4)	Iran Medical University	Nursing students	180 (72%; 28)	Iran	No significant relation between students' sex and the frequency of insult (p = 0.051)	X		X
2.	Brown et al., 2019 (5)	Gynecology	Physicians- members of an international society	907 (59%; 40%; unknown 1%)	USA and non-USA	Gender discrimination (90%; 72%)<br>Harassment (72.6; 53)<br>Sexual Harassment (84%; 14%)			X
3.	Chang et al., 2020 (6)	4 Universities	Senior nursing students	310 (87%; 13%)	Taiwan	Sexual Harassment (23.3%; 18.4%)	X		X
4.	Newman et al., 2021 (7)	Health sector	Healthcare providers, including medical personnel	294 Data presented: men and women	Uganda	Sexual coercion started during recruitment of health workers and continued after hiring, perpetrated by men in decision-making positions.			X
5.	Al-Ghabeesh & Qattom, 2019 (13)	Emergency department	Nurses	120 (35%; 65%)	Jordan	No significant differences based on the gender of the participant (p=0.07).			X
6.	Sellers et al., 2012 (18)	19 Healthcare organizations	Registered nurses	2659 (93%; 7%)	New York State, USA	Women reported significantly greater (p < .05) knowledge of and being a victim of HV than men.		X	
7.	Alameddin et al., 2015 (19)	Database of the Order of Nurses in Lebanon	Nurses	593 (79%; 21)	Lebanon	Male nurses had 2.22 times the odds of exposure to physical violence compared to females (95% CI 1.14–4.35, p- 0.019).	X	X	X
8.	Al.Surimi et al., 2020 (20)	4 hospitals in various regions	Nurses, Physicians, and others	1075 (86%; 14%)	Saudi Arabia	Bullying (66%; 49%)	X	X	X
9.	Anand et al., 2016 (21)	Tertiary care hospital	Medical residents- various departments	169 (38.5%; 61.5%)	India	WPV (40%; 41%); Threats (58%; 47%)<br>Physical (4%; 16%); Verbal (17%; 81%)	X	X	X
10.	Boafo et al., 2016 (22)	Hospitals at all levels	Nurses	592 (79%; 21%)	Ghana	Verbal Abuse (83;17.0%).	X	X	X
11.	Brooks et al., 2022 (23)	Orthopedics	Black orthopedic residents and fellows	310 Residents (18%; 82%); Attending (22%; 78%)	USA	Micro assaults (65%; 60%)	X	X	X
12.	Campbell et al., 2011(24)	4 health care institutions	Nursing personnel (all categories of nurses)	2166 (91.5%; 8.5%)	USA	Males were nearly twice as likely to have experienced physical WPV compared to females.	X	X	X
13.	Cavalcanti et al., 2018 (25)	PHC	Nurses	112 (95%; 5%)	Brazil	Workplace violence (72%; 83%)	X	X	X
14.	Ceppa et al., 2020 (26)	Cardiothoracic surgery	Attending surgeons and trainee surgeons	790 (23%; 75%; Others 2%).	Globally-professional platforms	Sexual Harassment (81%; 46%) attending surgeons)<br>Sexual harassment (90%; 32% among trainees)	X	X	X
15.	Chatziionnidis et al., 2018 (27)	20 NICUs	Nurses and Physicians	398 (87%; 13%)	Greece	Bullying (56%; 36%)	X	X	X
16.	Chen et al.,2021(28)	Plastic surgery	Plastic surgery trainees	236 (42%; 54%; Others 4%)	Pittsburgh-USA	Had been presented provocative imagery/words (42%; 32%).<br>Discomfort from sexually oriented jokes (45%; 33%)	X	X	X

17.	Cheung and Yip, 2017 (29)	Members of the Association of Hong Kong	Nurses	850<br>(88%; 12%)	Hong Kong, China	Workplace Violence (44%; 48.6%)	X	X	X
18.	Cho et al., 2020 (30)	Hospital	Early-career hospital nurses	1171<br>(86%; 14%)	USA	**Verbal Abuse from patients/family**<br>1–3 times per month (58%; 49%)<br>Once a week or more (20%; 32.7%)<br>**From Physicians:**<br>1–3 times per month (38%; 34%)<br>Once a week or more (5%;9%)		**X**	**X**
19.	Crutcher et al., 2011 (31)	Family medicine	Family medicine residency graduates	242<br>(53.2%; 46.4%), Not recorded 0.4%)	Alberta, Canada	IHD by gender (48%; 44%)<br>IHD in the form of work as punishment (20%; 38.6%)<br>IHD in the form of privileges/opportunities being taken away (26.7%; 6.8%)	X	X	X
20.	Dettmer et al., 2021 (32)	Cardiology	Physicians	567 (49%; 51%)	Germany	Experienced sexual harassment (32%; 7%)		X	X
21.	Ferri et al., 2020 (33)	Emergency Triage area	Nurses	27<br>(44%; 56%)	Italy	Verbal (83%; 100%)<br>Both verbal and physical (17%; 0%)	X		
22.	Firenze et al., 2020 (34)	Hospitals	Medical personnel	4545<br>(57%; 43%)	Italy	Males experience almost three times higher (aOR 2.09, 95% CI 1.51-2.88, $p<0.001$)	X	X	X
23.	Harthi et al., 2020 (35)	Public hospitals ED	HCWs in ED, including nursing and medical personnel	324<br>(66%; 34%)	Saudi Arabia	Workplace violence (42.8%; 57.8%)<br>Physical violence (11%; 20%); Verbal abuse (35%; 47%)	X	X	X
24.	Hu et al., 2019 (36)	General surgery residency programs.	Medical residents	7409<br>(39.6%; 59.9%; No data 0.5%	USA	Gender discrimination (65.1%;10.0%).<br>Verbal or emotional Abuse (33.0%; 28.3%)<br>Sexual harassment (19.9%; 3.9%)	X	X	X
25.	Kemper & Schwartz, 2020 (37)	Pediatrics	Pediatric residents-Resident Burnout and Resilience Study Consortium	1956<br>(70%; 30)	USA	Any type of Mistreatment 33% (36%; 25%)<br>Bullying19% (20%; 16%)<br>Discrimination 18% (21%; 11%)<br>Sexual Harassment 5.4% (6%; 4%)	X	X	X
26.	Lei et al., 2022 (38)	Emergency department	Nurses	20136<br>(89%; 11%)	China	Any type of WPV (89%; 11%)<br>Physical (85%; 15%); non-physical (89%; 11%)	X	X	X
27.	Menhaji et al., 2022 (39)	OBGYN	Trainees	366<br>(86.5%; 13.5%)	USA	Sexual harassment (68.7%; 69.6%)	X	X	X
28.	Obeidat et al., 2018 (40)	Private hospitals	Registered nurses	274<br>(49%; 51%)	Jordan	Men were more likely to report a higher overall rate of perceived workplace bullying ($p < 0.001$) than women.	X	X	X
29.	Orlino et al., 2022 (41)	Vascular surgery	Medical Trainees	132<br>(31%; 69%);	USA	Experience of Bullying (36%; 30%)	X	X	X
30.	Pinar et al., 2017 (42)	All levels healthcare institutions	Health personnel	12,944<br>(60%; 40%)	Turkey	Workplace violence in 12 months (48%; 39.5%)<br>Violence during the career (54.3%; 49.4%)	X	X	X
31.	Scruggs et al., 2020 (43)	Ophthalmology	Trainees	112<br>(47%; 53%)	USA	Sexual harassment from patients (86.8%; 44.1%).<br>Physical harassment (24.5%; 8.5%)	X	X	X
32.	Sharma et al., 2021(44)	Cardiology	Cardiologists	5931<br>23%; (77%)	Globally	Gender discrimination/sexual harassment (57%; 22%)<br>Hostile work environment (68%; 37%)<br>Sexual harassment (12%; 1%).	X	X	X

33.	Siller et al., 2017 (45)	A medical university	Students	88 (51%; 49%)	Austria	Harassment and sexual mistreatment (68.9%; 32.6%). Humiliation (77.8%; 53.5%).		X	X
34.	Simoes et al., 2020 (46)	Primary and secondary care	Nursing, medical, and other personnel	203 (71%; 29%)	Brazil	Some form of Abuse 40.4% (48%; 22%).	X	X	X
35.	Subbiah et al., 2022 (47)	Oncology	Oncologists	271 (56%; 44%)	USA	Sexual harassment-peers and superiors (80%; 56%) Sexual harassment-patients and families (67%; 35%)	X	X	X
36.	Swed et al., 2022 (48)	Graduate medical education	Medical residents and fellows	276 (58%; 38%); missing (3.6%)	Syria	Bullying (54%; 30%)	X	X	X
37.	Tekin and Bulut, 2014 (49)	Operating room	Nurses	360 (92%; 8%)	Turkey	A significant relationship- between gender and educational status ($p<0.05$); women were more exposed to verbal abuse.		X	X
38.	Urnberg et al., 2022 (50)	All sectors, public and private	Physicians	2786 (67%; 33%)	Finland	Overall aggression (73%; 27%) Physical Aggression (69%; 31%) Non-physical Aggression (77% 23%)	X	X	X
39.	Zampieron et al., 2010 (51)	All levels of health care institution	Nursing personnel	579 (79%; 21%)	Italy	Aggression (52%; 42%) Verbal aggression (82.8%; 78%)	X	X	X
40.	Speroni et al., 2014 (52)	Multiple-hospital system	Nurses	762 541 (93%; 7%)	USA	Workplace violence 76% (93%; 7%)	X		
41.	Wang et al., 2020 (53)	Vascular surgery	Vascular residents	284 (36%; 64%)	USA	Sexually harassment (25%; 1%) GBDB during training (80%; 14%) Some form of public humiliation (64%, 49%)	X		X
42.	Weldehawaryat et al., 2020 (54)	Public health facilities	Nurses	348 (57%; 43%)	Ethiopia	WPV (61%;39%)	X		X
43.	Feng et al., 2022 (55)	GPs	GPs	4376 (59%; 41%)	China	Any type (49%; 51%) Physical (37%; 63%); Non-physical (49%; 51%)	X		
44.	Özdamar Ünal et al., 2022 (56)	Various healthcare settings	Physicians and others	701 (68%; 32%)	Turkey	Workplace violence (70%; 30%)	X		
45.	Turgut et al., 2021(57)	Emergency department	Physicians	157 (37.6%; 62.4)	Turkey	Violence-reported cases (37.6%; 62.4%)	X		
46.	Vezyridis et al., 2015 (58)	emergency departments	Nursing and medical personnel and a few others	220 (62%, 38%)	Cyprus Republic	No significant differences between the participant's gender.	X	X	
47.	Elston & Gabe, 2016 (59)	Primary health care	GPs	697 (37%; 62%)	South-east England, UK	Physical assault (7%; 13%); Threat of harm (8%; 33%) Verbal abuse (78%; 74%) Afraid of becoming a victim of violence (76%;60%)	X		
48.	Oguz et al., 2020 (60)	Pediatric clinics	Medical and nursing personnel and others	182 (78.5; 21.5%)	Turkey	Violence (72%; 27%)	X		
49.	Newman et al., 2011 (61)	Rural and urban settings	Midwives, Nurses, Physicians	297 (69%; 31%)	Rwanda	Verbal abuse: (68%; 32%); Bullying: (66 %; 34%); Sexual harassment: (75%; 25%); Physical attacks: (64%; 36%)	X	X	X
50.	Arnold et al., 2020 (62)	Pediatrics, internal medicine, and surgery Residents	Physicians	381 (60%; 40%)	USA	Some sort of harassment during both medical school and residency (55.8%; 35.6%) Sexual harassment (83%; 44%)	X		X

51.	Benzil et al., 2020 (63)	Neurosurgery	Surgeons	622 (21%; 78%; others 1%)	USA-professional platforms	Sexual harassment (88%;44)		X	X
52.	Freedman-Weiss et al., 2020 (64)	Surgical training programs	Trinee residents	270 (44%; 53%) others; 3%	USA	Sexual Harassment 49% (70.8%; 30.8%)		X	X
53.	Nukala et al., 2020 (65)	Vascular surgery	Medical Trainees	133 (37%; 61%; others 2%)	USA	Sexual harassment (52%; 23%)		X	X
54.	Smed & Aulivola 2020 (66)	Vascular surgery	Faculty of training programs	149 (22%; 8%)	USA	Sexual harassment (67%;34%)		X	X
55.	Crebbin et al., 2015 (67)	Surgery	Medical personnel in surgery	3516 (19%; 81%)	Australia and New Zealand	Overall prevalence of DBSH (72%; 64%)<br>Bullying (58%; 34%); Sexual Harassment (30%; 2%)		X	X
56.	Jain et al., 2019 (68)	Ophthalmology	Ophthalmologists	282 (32%;68%)	Australian	Bullying (43%; 33%); Discrimination (31%; 8%)<br>Sexual harassment (23%; 0.5%)			X
57.	Lall et al., 2021(69)	Emergency	EM residents	8470 (35%; 62%), Unknown (3%)	USA	Gender discrimination (65%; 9%)<br>Verbal or emotional abuse (32 %; 27%)	X		X
58.	Picakciefe et al., 2017 (70)	Primary health care	Health personnel	119 (83%;17%)	Turkey	Mobbing 31% (89%; 11%)			X
59.	Vorderwulbecke et al., 2015 (71)	Primary health care	Primary care physicians	1500 (40%; 60%)	Germany	Aggression (60%; 51%);<br>Sexual harassment (25%; 15%)	X		
60.	Xie et al., 2017 (72)	Medical school	Medical students'	180 (56%; 445)	China	All types of violence (29%; 33%)<br>Sexual harassment (9%; 10%)<br>Physical violence (15%; 3%)	X		
61.	Al-Maskari et al., 2020 (73)	Emergency department	Nurses	103 (74%; 26%)	Oman	Physical: (47% 53%)<br>Non-physical: (24%; 76%)	X		
62.	Ferri et al., 2016 (74)	General Hospital	Nursing and medical personnel	419 (67%;33%)	Italy	WPV (assaulted): 45% (72%; 28%)	X		
63.	James et al., 2011(75)	Psychiatric in-patient settings	Mental health nurses	76 (71%; 31)	Nigeria	Male nurses reported significantly higher episodes of aggressive spitting behaviour ($p<0.011$) as well as physical violence ($p<0.010$).	X		
64.	Hsiao et al., 2021(76)	University of Florida College of Medicine	Medical personnel	509 (54%; 46%	USA	Sexual harassment (46.2%;19.4%)	X	X	
65.	Meyer et al., 2021(77)	Ophthalmology	Trainees and Ophthalmologist	**In 2015**-582 (29%; 71%)<br>**In 2018**-560 (29%; 71%)	Australia and New Zealand	Sexual harassment (32%; 4%).<br>Discrimination (43%;12%)<br>Bullying (51%; 31%)			X
66.	Hu et al., 2022 (78)	Association of American Medical Colleges	Physicians	6000 (29%; 66%); non-binary	USA	Greater representation of women within a specialty is associated with a lower prevalence of harassment experienced by men and women physicians (e.g., threats of physical harm for women (OR= 0.973, CI 0.954-0.992) and men (0.984, CI 0.974-0.993) and unwanted sexual advances for women (OR = 0.976, CI 0.967- 0.984) and men (0.988, CI 0.981-0.995).	X	X	X
67.	Forrest et al., 2011(79)	All urban and rural settings	General practitioners	804 (51%; 49%)	Australia	Verbal Abuse (56%; 58%); Physical Abuse (4%;7%), Stalking (3%;4%); Sexual Harassment (10%;2.5%)	X		

68.	Abed et al., 2016 (80)	Primary Care clinics	Nurses and physicians	102<br>(86%; 14%)	Barbados	Any type of violence (71%; 21%)<br>Verbal abuse: (67%; 21%)	X	X	
69.	Vyas et al., 2022 (81)	Tertiary Care Hospital	Nurses, Physicians, and others	157<br>Sample not sex-segregated	Uttarakhand North India	Overall violence (65%; 35%)<br>Verbal violence (62%; 38%); Bullying (100%; 0%)<br>Physical violence (60%; 40%)	X		
70.	Giglio et al., 2022 (82)	Orthopedics	Physicians	465<br>(28%; 72%), NB:1 (0.2%)	Canada	Gender-based harassment (98%; 68)<br>Sexual harassment (83%; 71)	X	X	X
71.	Abrams & Robinson, 2011 (83)	Urban settings	Physicians<br>(directory of physicians)	1190<br>(35%; 61%; unknown 4%)	Canadian urban area	Stalking (13.5%; 10.9%)	X		
72.	Afkhamzadeh, 2019 (84)	Teaching hospital	Physicians & medical students	321<br>(55%; 45%)	Iran	At least one type of violence (51%; 68%)	X		
73.	Alhamad et al., 2021 (85)	All kinds of hospitals	Physicians	969<br>(35%; 65%)	Jordan	Abuse: (55%; 67%)<br>Verbal abuse: (50%; 60%); Physical abuse: (01%; 08%)	X	X	
74.	Al. Shamlan et la., 2017 (86)	Teaching Hospital	Nurses	391<br>(89%; 11%)	Saudi Arabia	Verbal abuse (28%; 50%)	X	X	
75.	Balch Samora et al., 2020 (87)	Orthopedics	Surgeons	926<br>(67%; 33%)	USA	Overall DBSH: (81%; 35%); Discrimination (84%; 59%);<br>Sexual harassment (54%; 10%)			X
76.	Bhandari et al., 2021 (88)	Internal medicine	Hospitalists	336 (57%;43%)	USA	Discrimination (99%; 29%)<br>Harassment (72%; 36%,	X	X	
77.	Jaradat et al., 2016 (89)	Hospitals and primary care clinics	Nurses	343<br>(62%; 38%)	State of Palestine	Workplace Aggression (26%; 28%)<br>Physical aggression (5%; 5%)<br>Verbal Aggression 24%; 25%); Bullying (5%; 12%)	X	X	
78.	Cashmore et al., 2012 (90)	Correctional health services	Physicians and nurses, and others	208 incidents	Australia	Workplace violence incidents (66%; 34%)<br>Verbal Abuse (74%, 26%); Physical Abuse (25%; 55)	X		
79.	Chen et al., 2018 (91)	Tertiary teaching hospital	Nurses	1983<br>(92%; 8%)	China	Any type of violence (50%; 38%)<br>Non-physical (50%; 47%); Physical (6%; 9%)	X	X	
80.	Cheng et al., 2020 (92)	National Health Services	Nurses, Midwives	147<br>(28%; 18%)	England, UK	Aggression from Patients (26%; 20%)	X	X	
81.	Dal Pai et al., 2015 (93)	Hospital setting	Medical, nursing, and other health personnel	269<br>(58%; 42%)	Brazil	Exposed to Violence (71%; 52%)	X		
82.	Dehghan-chaloshtari and Ghoduosi, 2017 (94)	Hospital	Nurses	100<br>(76%; 24%)	Iran	Physical (82.5%; 17.5%);<br>Verbal violence (78.6%; 21.4%)<br>Bullying and mobbing (70.3%; 29.7%)<br>Sexual abuse (66.7%; 33.3%)	X	X	X
83.	Demeur et al., 2018 (95)	Flemish (Belgian Federal State)	GPs	248<br>(60%; 40%)	Belgium	Raising voice (71%; 29%); Scolding (63%; 37%)<br>Verbal intimidation (63%; 37%)<br>Violating privacy (60.5%; 39.5%); Touching (63%, 37%)<br>Grabbing, slapping & kicking (62%; 38%)<br>Sexual intimidation (70%; 30%)	X		
84.	Difazio et al., 2019 (96)	Diverse healthcare settings	Members of the Russian Nurses Association	438<br>(97.5%; 2.5%)	Russian Federation	Bullying (97.5%; 2.5%)		X	

85.	Fitzgerald et al., 2019 (97)	Academic teaching hospitals	Surgical Residents	76<br>(49%; 51%)	USA	At least one form of abuse and harassment (48%; 52.5%).<br>Discrimination in relation to gender (92%; 8%).	X		X
86.	Fnais et al., 2013 (98)	Residency programs in teaching hospitals	Trainee residents	213<br>(42%; 58%)	Saudi Arabia	Verbal harassment during training (76%; 51%)<br>Gender discrimination (69%; 57%)<br>Sexual harassment (28%; 13%)	X		X
87.	Fute et al., 2015 (99)	Public health facilities	Nurses	642<br>(63%; 37%)	Ethiopia	Workplace violence (36%; 20%)	X		
88.	Hills et al., 2012 (100)	Clinical medical practitioners	Medical personnel	9438<br>(43%; 57%)	Australia	Verbal or written aggression (72.6%; 69%)<br>Physical aggression (33.8%; 31.2%)	X	X	
89.	Honarvar et al., 2019 (101)	University-affiliated public hospitals	Nursing personnel	405<br>(81%; 19%)	Iran	Verbal Abuse (84%; 83%); Verbal threat (25.6%; 46.4%)<br>Physical violence (16.8%; 41.6%)<br>Sexual Harassment (9.8%; 15.6%)	X		
90.	Kisiel et al., 2020 (102)	Uppsala University	Medical students	**2002**-343<br>(55%: 45%)<br><br>**2013**-720<br>(62%; 38%)	Sweden	**2013** (pre-clinical group)<br>Discrimination (22%; 15%); Favoritism (23%; 18%)<br>Intrusive, unwelcome acts (21%; 15%)<br>**2013** (clinical group)<br>Discrimination (41% and 25%); Favoritism (53%; 33%)<br>Intrusive, unwelcome acts (26%; 20%)			**X**
91.	Lafta & Falah, 2019 (103)	Hospitals and primary healthcare centres	Medical, nursing personnel and others.	700<br>(51%; 49%)	Iraq	Physical violence (24%; 76%)<br>Verbal (53%; 47%)	X		
92.	Li et al., 2020 (104)	General Hospitals Survey	Nursing Workforce	396<br>(73%; 27%)	China	Verbal abuse from patients and/families (34%;30%)<br>Verbal abuse from staff (59%; 47%)<br>Physical abuse from patients/families (64%; 58%)<br>Physical abuse from staff (81%; 70%)	X	X	
93.	Li et al., 2010 (105)	Emergency Medicine	Residents	196<br>(53%; 47%)	USA	Sexual harassment 23% (37%; 8%).	X		
94.	Lu et al., 2020 (106)	Academic Emergency Medicine faculty	Emergency Medicine faculty	144<br>(39%; 61%)	England, UK	Discrimination based on gender (62.7%; 12.5%)<br>Unwanted sexual harassment behaviors (52.9%; 26.2%	X		X
95.	Lucas-Guerrero et al., 2020 (107)	General Surgery	Surgical residents	452<br>(66%; 34%)	Spain	Physical abuse (5.4%; 10.5%)<br>Sexual harassment (21.4%; 6.5%)<br>Discrimination (90.4%, 9.6%).	X		X
96.	Margavi et al., 2020 (108)	Emergency wards of teaching hospitals	Nurses	140<br>(61%; 39%)	Iran	Physical violence (43%; 76%)<br>Psychological violence (90%; 83%)<br>Sexual harassment (8%; 0%)<br>Bullying/mobbing (34%; 46%)	X		
97.	McKinley et al., 2019 (109)	Residency training programs	Medical residents	371<br>(46%; 53%);<br>Others 2)	Massachusetts, USA	Gender-based discrimination (93%; 24%).<br>Sexual harassment during training (34%; 5)	X		X
98.	Mirza et al., 2012 (110)	Emergency department	Physicians in training	675<br>(47%; 53%)	Pakistan	Verbal abuse (61; 63%)<br>Physical abuse (8%; 15%)	X		
99.	Moman et al., 2020 (111)	Pain management clinicians	Medical and nursing personnel	58<br>(41%; 59%)	Conference participants, USA	Experienced assault (59%; 75%)	X		

100.	Moylan et al., 2014 (112)	Psychiatric facilities	Nurses	110 (85%; 15%)	New York, USA	Physical assault 73% (80%; 20%)	X		
101.	Nieto-Gutierrez et al., 2018 (113)	Residency programs	Medical residents	1054 (38%; 62%)	Peru, South America	Workplace Violence (75%; 72%)	X	X	
102.	Park & Choi, 2020 (114)	General Hospital	Nurses	205 (89%; 11%)	South Korea	Mean experience of Verbal violence (23, 29)<br>Mean Doing Verbal violence (19, 24)			X
103.	Pol et al., 2019 (115)	Intensive Care Unit	ICU Nurse clinicians	47 Patient records	Australia	Verbal violence (20%; 66.7%)<br>Physical violence (45.7%; 25%)	X		
104.	Prajapati et al., 2013 (116)	All kinds of health facilities	Medical, nursing, midwifery, and other personnel	747	Nepal	Gender-based harassment (62.5%; 37.5%)<br>Sexual Harassment (56.5%; 43.5%)	X		
105.	Rosta & Aasland, 2018 (117)	Hospital	Medical personnel	**1993=** 2439 (28%; 72%)<br>**2004**= 730 (31.5%; 68.5)<br>**2014-15**= 1080 (43%; 67%)	Norway	Perceived Bullying in:<br>1993= (8.3%; 4.7%)<br>2004= (4.8%; 8.4%)<br>2014-15= (9.2%;5.4%)		X	X
106.	Rouse et al., 2016 (118)	Academic settings	Family physicians	1065 (43%; 57%)	USA	Ever displayed bullying behaviors (7.7%; 11.2%)<br>Ever been bullied (34%; 24.7)		X	
107.	Sachdeva et al., 2019 (119)	Emergency Department	Medical and nursing personnel	335 (34%; 66%)	India	Verbal abuse (37%; 63%); Physical abuse (32%; 68%)<br>Confrontation (12%;88%)	X	X	
108.	Sakellaropoulos et al., 2011 (120)	Anesthesia	Certified registered nurse anesthetists	205 (62%; 37%)	USA	Verbal aggression (89%; 83%)		X	X
109.	Tian et al., 2020 (121)	Various Hospitals	Nursing and medical personnel	3684 (85%; 15%)	China	Emotional abuse (47.3%; 55.4%); Threats (25%; 38%)<br>Physical Abuse (14.5%; 24%); Sexual Abuse (7%; 13%)	X		
110.	Vargas et al., 2020 (122)	University Medical School	Faculty members	705 (48; 52%)	USA	Sexual harassment from insiders (82.5%; 65.1%)<br>Sexual harassment from patients (64.4%; 44.1%)<br>Gender harassment from insiders (82.2%; 64.9%)<br>Gender harassment from patients (64.0%; 44.1%)	X	X	X
111.	Viottini et al., 2020 (123)	University Hospital Network	Midwives, nurses and physicians	364 (77.5%; 22.5)	Italy	Assaults incidences (77.5%; 18.5%)	X	X	
112.	Williams et al., 2021 (124)	Medical residency program	Internal Medicine residents	33 (41%; 59.3%)	USA	Microaggression by a patient (90.9%; 56.3%)	X		
113.	Camm et al., 2022 (125)	Cardiology	Trainees	1359 (27%; 73%)	UK	Sexist language (14%; 4%)			X
114.	Rowe et al., 2022 (126)	Large academic medical center	Physicians	1505 (49%; 42%); unknown: 143 (9%)	California, USA	Any forms of mistreatment (31.0%; 15.0%)<br>Sexual harassment (8.8%;1.5%)<br>Verbal abuse (28%; 14%); Physical abuse (6%; 4%)	X	X	
115.	Vidal-Alves et al., 2021 (127)	Public hospitals	Nursing staff	950 (78%; 22%)	Southeast of Spain	Mean- personal lateral violence (3.06; 3.41)<br>Mean- social lateral violence (1.92; 1.57)<br>Mean -work-related lateral violence (1.51; 1.28)		X	
116.	Kibunja et al., 2021(128)	Emergency department	Nurses	82 (65%; 35%)	Kenya	Physical violence (71%; 29 %);Verbal abuse (65%; 35%);<br>Sexual harassment (82%; 18%)	X		
117.	Sabak et al., 2021 (129)	ED	Physicians	362 (38%;62%)	Turkey	Verbal threats (100%; 97%); Sexual harassment (5%;7%)<br>Physical assaults (50%; 57%); Stalking (16%; 30%)	X		

118.	Gadjradj et al. 2021(130)	Neurology	Neurosurgeons and neurosurgical residents	503<br>(20%80%)	e-survey for conference participants	Gender discrimination (90.2 %; 13.0 %)	X	X	X
119.	Albuainain et al., 2022 (131)	Surgical environments	Physicians	788<br>(35%; 65%)	Saudi Arabia	Negative Attitude Questionnaire-R score (42.7; 42.3)		X	X
120.	Nøland et al. 2021(132)	2-cohorts of medical students	Physicians	893<br>(56%; 43%)	Norway	Prevalence of WPV T2= (14.5%; 27.7%); T3 (11.3%; 25.0%); T4 (9.1%; 14.4%); T5 (7.3%; 10.5%)	X		X
121.	Papantoniou, 2022 (133)	Greek NHS	Nurses	1726<br>(71%; 29%)	Greece	Sexual harassment (67%; 41%).			X
122.	Kowalczuk & Krajewska-Kulak, 2017 (134)	General Hospital	Medical, nursing, midwifery, and other personnel	1624<br>Medical (56%; 44%)<br>Nurses (98%; 2%)<br>Midwives (99.6%; 0.4%)	Poland	Mean scores of patient aggression 0-5 scale<br>Nurses (26.6; 34.9)<br>Physicians (17.8; 19.7)<br>Midwives (12; 10.9)	X		
123.	Notaro et al., 2021(135)	Dermatology	Dermatologists and trainees.	330<br>(75%; 24%, 4-unknown)	USA	Sexual harassment (94%; 52%)<br>Sexual assaults (35%; 15%)	X		
124.	Schlick et al., 2021 (136)	301 general surgery programs	Medical residents	6956<br>(41%; 59%)	USA	Gender discrimination (80%; 17%)<br>Sexual harassment (43%; 22%)	X	X	X
125.	Hunter et al., 2022 (137)	Higher Education Institution	Nursing students	138<br>(92%; 8%)	Scotland, UK	Ever experienced Verbal violence (70%; 67%).<br>Ever experienced Physical violence (72%; 41%)	X		
126.	Ferrara et al., 2021(138)	Academic	Nursing students	603<br>(77%; 23%)	Italy	Psychological violence (39%; 22%)<br>Physical violence (9%; 5%)	X	X	X
127.	Pendleton et al., 2021 (139)	Three academic institutions	Medical residents from 12 programs	309<br>(55%; 45%)	Boston, USA	Gender-based discrimination (100%; 69%)	X		
128.	Snavely et al., 2021 (140)	Emergency medicine (EM)	Resident trainees	22<br>(64%; 36%)	New York, USA	Mean number of incidents/shift (3.0; 0.9)	X		
129.	Hock et al, 2021 (141)	ophthalmology, surgery, medicine and others	Medical residents and faculty	91<br>(50%; 48%; No response 2%)	Iowa, USA	Recognition for patient-initiated harassment on a 5-point Likert scale before the workshop (4.0; 3.7) and after workshop participation (4.6; 4.5)	X		
130.	Rodriguez-Acosta et al., 2010 (142)	University Hospitals	Nursing staff	220 injuries<br>(86%; 14%)	Duke, North Carolina, USA	While the number of assaults was greater among women than men, their risk was lower (RR=0.70).	X		
131.	Hahn et al., 2013 (143)	University general hospital settings	Physicians, nurses and midwives	2495<br>(82%; 18%)	Switzerland	Gender factored WPV (no clear description provided)	X		
132.	Wang et al., 2022 (144)	ICU	Nurses	305<br>(68%; 32%)	China	There were increased odds of experiencing WPV among nurses with lower professional titles, male nurses (OR=2.7, CI=1.310 to 5.944), and those with less than five years of experience.	X		
133.	Wright & Khatri, 2015 (145)	Teaching hospital network	Nurses	1078<br>(91%; 9%)	USA	Male nurses experienced significantly higher work-related bullying than female nurses p<.067). No significant differences in person-related bullying, which had a significant positive relationship with both psychological/behavioral responses and medical errors.			X

134.	Bambi et al., 2014 (146)	Prehospital EMS, emergency department, ICU, and OR	Nurses	1202 (61.5%; 38.5%)	Italy	Desire to leave the nursing profession because of the LH was (15.5% and 9%); however, gender was not statistically significant for LH.		X	
135.	Koukia et al., 2014 (147)	General Hospital	Healthcare staff, including nursing and medical personnel	250 (74%;26%)	Greece	Women were more likely to experience sexual (p<0.012) and physical violence (p<0.014).		X	X
136.	al-Omari, 2015 (148)	11 General hospitals	Nurses	468 (47%; 53%)	Jordan	Female nurses were 0.5 times less likely to report being physically attacked than male nurses (p=0.003). Female nurses were 1.5 times more likely to report being verbally abused than male nurses (p=0.046).	X		
137.	Esmaeilpour et al., 2011 (149)	Emergency department	Nurses	186 (89%; 11%)	Iran	Male nurses were the victims of physical violence more often than female nurses (p=0.000).	X		
138.	Joa and Morken, 2012 (150)	Out-of-Hours primary care centres	Physicians, nurses, and others	536 (70%; 30%)	Norway	Men were more at risk of physical abuse (OR=2.36, CI 1.11-5.05) and verbal abuse (OR=1.23, 0.68-2.18).	X		
139.	Serafin & Czarkowska-Pączek, 2019 (151)	Polish healthcare facilities	Nursing personnel	411 (96%; 4%)	Poland	Women were more often affected by 'being humiliated or ridiculed in connection with their work' (p=0.040), 'being ordered to do work below their level of competence' (p=0.010), and 'having key areas of responsibility removed or replaced with more unpleasant tasks' (p=0.005).			X
140.	Kelly et al., 2015 (152)	Forensic hospital	Overall staff	488 (69%; 31%)	California, USA	Men experienced higher scaled frequencies of assault than women (4-point Likert scale, 0-3] mean = (0.46 vs 0.33, p = 0.02).	X		
141.	Fafliora et al., 2015 (153)	Primary, secondary, and tertiary care hospital	Nurses	80 (83%;17%	Greece	Men (OR, 0.08, CI 0.01-0.56) and higher experience nurses (OR, 0.82, CI 0.70-0.097) were less affected by WPV.	X	X	
142.	Askew et al., 2012 (154)	All doctors at Australian Medical Board	Doctors from various department	747 (53%; 47%)	Australia	No significant differences in the prevalence of bullying between the sexes. Victims of bullying had poorer mental health (p<0.001)			X
143.	Lindquist et al., 2020 (155)	National emergency medicine conference	Physicians and medical students	63 (56%; 41%; Missing: 3%)	Myanmar	Women were more likely to experience verbal assault (OR=1.18, 0.42-3.33).	X	X	
144.	Yohe et al., 2020 (156)	Orthopedic residency	Orthopedic residents	1207 (17%; 83%)	USA	Gender was not statistically significant (OR=1.07, 0.63-1.84, p-0.79).	X		X
145.	Farid et al., 2021(157)	Obstetrics and gynecology	Physicians	87 (75%; 25%)	USA	Most physicians (71%) had ever experienced discrimination-attributed to their gender.	X	X	
146.	Vingers 2018 (158)	Nursing Education	Nursing students	107 (87%; 13%)	USA	There was no significant difference in the frequencies of reported bullying behaviors for male and female nursing students (< .05 level).	X		X
147.	Dafny& Beccaria, 2020 (159)	Three regional hospitals	Nurses	23 (74%; 26%)	Australia	Five themes identified including, perpetrators of violence, and gender and the incidents of violence	X		

148.	Ko & Dorri, 2019 (160)	Primary care	Clinicians (nurses & Physicians)	26 (62%; 38%)	USA	Participants reported experiences of bias, harassment, and discrimination based on gender, race/ethnicity, and/or sexual orientation and gender identity.		X	
149.	Neiterman & Bourgeault, 2015 (161)	IMGs and IENs	Medical and nursing personnel	140<br>IMG 69 (36%;11%)<br>IENs 71 (60%; 11%)	Canada	Physicians were concerned with instances of discrimination within their own professional group. Nurses, on the other hand, reported discrimination at the hands of patients and their families as well as racialization by physicians, management, and other nurses.	X	X	X
150.	Al Khatib et al., 2023 (162)	ED- Public hospitals	Physicians and nurses	163<br>(27%; 73%)	Jordan	Physical (2.3%; 43.7%); Verbal (29.5%; 61.3%)	X	X	X
151.	Al-Wathinani et al., 2023 (163)	ED	Physicians, nurses and others	206<br>(43.2%; 56.8%)	Saudi Arabia	Physical assault (48.3%; 66.7%)	X		
152.	Alhassan et al., 2023 (164)	ED	Midwives, nurses, physicians and others	7398<br>(48.7%; 51.3%)	Saudi Arabia	Physical attacks (44%; 56%)	X		
153.	AlHassan et al., 2023 (165)	ED	Midwives, nurses, physicians and others	7398<br>(48.7%; 51.3%)	Saudi Arabia	Sexual attack (61%; 39%)	X	X	
154.	Banga et al., 2023 (166)	Health workers	Nurses, physicians and others	5405<br>(53%; 45%; others 2%)	79 countries	Verbal violence (50.8%; 51%); Physical abuse (19%; 24.2%); Emotional violence (30.6%; 28%); Sexual violence (7.4%; 3.8%)	X	X	X
155.	Barequet et al., 2023(167)	Ophthalmology	Physicians	252<br>(46%; 54%)	Israel	Physical Abuse (18%;13%); Sexual harassment (50%;13%)	X	X	
156.	Bekalu et al., 2023 (168)	Public hospitals	Nurses	534<br>(45%; 55%)	Northeast Ethiopia	Violence (58%; 42%)	X	X	
157.	Crombie et al., 2024 (169)	University	Medical students	443<br>(73%; 27%)	South African	Mistreatment (80.9%; 70.8%)		X	X
158.	DiFiori et al., 2023(170)	Orthopaedic surgery	Surgeons, fellow and residents	105<br>(12%; 84%; others 6%)	USA	Demographic information including sex did not have a statistically significant (p-0.167) association with self-reported bullying	X	X	
159.	Domínguez et al., 2023 (171)	Surgery	General surgery residents	302<br>(42%; 58%)	Colombia	Occasional bullying (26%; 29%); Continuous bullying (22%;21%); Sexual harassment (29.1%; 4.55)		X	X
160.	Ebrahim et al., 2023 (172)	Healthcare workers-Hospital	Nurses, physicians and others	5537	Kenya	Discrimination based on gender (27.3%; 20.2%) Physical abuse (5.4%; 3.8%); Verbal abuse (60.9%; 48.9%)	X	X	X
161.	Forsythe et al., 2023 (173)	Vascular diseases physicians	Consultant, fellows, Residents, interns/ students	587<br>(35.8%; 62.9%; other 1.5%)	International (28 countries)	Experiences of Bullying, undermining behaviour, and harassment (53%; 38%)	X	X	X
162.	Grover et al., 2023 (174)	General surgery and urology	Residents	23<br>(35%;65%)	Mid-Atlantic	Mistreatment (88%; 33%); Verbal assault (50%; 33%)	X		
163.	Ioanidis et al., 2023 (175)	Otolaryngology	Residents and attending Physicians	183<br>(55.6%; 45.4%)	Canada	**Harassment during residency** (75.2%;37.9%)<br>Verbal (87%; 92%); Sexual harassment (45%; 11%)<br>**Harassment- attending physicians** (68%; 31.6%)<br>Verbal (93%; 65%); Sexual harassment (41%; 22%)	**X**	**X**	**X**

164.	Janatolmakan et al., 2023 (176)	Emergency	Hospital nurses	150<br>(58.7%; 41.3%)	Iran	Physical Violence (60.2%; 39.8%)<br>Verbal Violence (58.5%; 41.5%)	X	X	X
165.	Meese et al., 2024 (177)	Healthcare	Healthcare workers, including Nurses	2659<br>(60.5%; 39.5%)	USA	Verbal Mistreatment (26.5%; 19.3%)<br>Physical Violence (17.1%; 10.7%)	X		
166.	Nam et al., 2023 (178)	Departments of Urology and Obgyn at the University	Clinicians	128<br>(76%; 24%)	Michigan	**Urology:** Unwanted sexual attention (68.8%; 22.7%) Gender harassment (84.4%; 40.9%); Sexual Coercion (15.6%; 0%)<br>**OBGYN**; Unwanted sexual attention (68.8%; 54.6%) Gender harassment (84.4%; 68.2%); Sexual Coercion (3.1%; 0%)	X		
167.	Parodi et al., 2023 (179)	Health sector	Physicians and nurses	3056<br>(57%; 43%)	Latin America	WPV (65.8%; 50.4%) ; Verbal violence (97%; 97%)<br>Physical Violence (10%; 9%)	X		
168.	Rashid et al., 2023 (180)	Cardiology departments	Junior physicians	1852<br>(43%; 57%)	Pakistan	Bullying (13.4%; 10.2%)			X
169.	Ryan et al., 2023 (181)	Academic orthopedics	Nurses residents/ fellows, and physicians	173<br>(65%; 45%)	USA	Harassment (all staffs): (27%; 24%)<br>Harassment (residents and faculty) (46%; 24%)	X		
170.	Santosa et al., 2023 (182)	Academic surgery programs	Faculty and residents	Residents/ fellows: 143<br>(58%; 42%)<br>Faculty: 183<br>(41%; 59%)	USA	Incivility experiences among surgeons (77%; 6%)	X	X	X
171.	Shahjalal et al., 2023 (183)	Tertiary care hospitals	Physicians	406<br>(49%; 51%)	Bangladesh	Physical Violence 36%; 64%)	X		
172.	Tavolacci et al., 2023 (184)	Health Campus & nursing school	Midwifery, nursing and medicine students	1152<br>(82.6%; 17%; others: 0.4%)	France	GBV (93.7%; 5.4%)	X	X	
173.	Veronesi et al., 2023 (185)	Two public referral hospitals	Nurses, physicians and others	7982<br>(74.7%; 25.3%)	Italy	WPV (65.7%; 34.3%); Physical violence (47.7%; 67.2%); Verbal (92.3%; 88.1%)	X	X	X
174.	Vu et al., 2023 (186)	University	Medical students	550<br>(75%; 25%)	Vietnam	Physical violence (10%; 22.5%) ; Verbal Violence 25.7%; 29.7%) ; Sexual violence (4.6%; 5.8%)	X	X	X
175.	Yan et al., 2023 (187)	Emergency	Physicians	14848<br>(29.5%; 70.5%)	China.	Any Type of violence (87.7%; 91.6%)<br>Physical (37%; 57.5%); Non-physical (87.2%; 91.2%)	X		
176.	Jaradat et al., 2016 (188)	Hospitals and primary care clinics	Nurses	341<br>(62%; 38)	State of Palestine	There were no significant differences between sex and workplace aggression resulting in psychosomatic symptoms (raged from 0.04-0.09).	X	X	
177.	Li et al., 2021(189)	Emergency department	Emergency room nurses	132<br>(91%; 9%)	Taiwan	Mental violence (54%; 50%)<br>Physical violence (12.5%; 8.3%)	X	X	X
178.	Zachariadou et al., 2018 (190)	Primary Health care clinics and general hospitals	Medical, Nursing, and other personnel	167<br>(71%;29%)	Cyprus	At least one mobbing behavior (49%; 35.7%)			X

Perpetrators for the Three Types of Violence

A total of 178 studies provided information on the perpetrators of either Type II (consumers/patients, including patients' companions), Type III-A (from colleagues), and Type III-B (from administrators and superior authorities within and between professions) violence. Studies included in this review did not consistently provide data for all types of violence and perpetrators; instead, they provided data for any Type/s. Of 178 studies, 141 (79%) reported perpetrators for Type II violence, followed by 93 (52.2%) for Type III-B (vertical) and 92 (51.6%) for Type III-A (horizontal) violence. Only 40 (22.5%) studies (7,19–51,162,166,170,175,176,189) reported information about all three types of violence.

While the search terms yielded many studies, there was significantly less information on the gender of perpetrators of WPV. Of the 178 studies reported on perpetrators, only 34 studies provided data for perpetrators' gender (detailed in Table 3). Across the three types of violence, more men (65%) were responsible for perpetrating WPV compared to women (28%). Both men and women perpetuated violence in the remaining 7% of cases.

Of the 34 studies, 25 studies reported on Type II violence, predominantly perpetrated by men, encompassing general violence (35,38,51–58), physical violence (4,33,42,59–61,176), verbal violence (4,33,42,51,61,170,176), and sexual harassment (6,39,42,61,62). In most of these studies, women experienced a higher prevalence of violence than men. Gender-based workplace violence against nurses emerged as a pressing issue for Type II (56.2%) violence in nine studies (4,6,33,38,51–54,166,176); men perpetrated 80% of the violence while women were responsible for only 19% violence, and almost all studies reported a higher prevalence of WPV against female nurses. A recent study (166) from 79 countries, though reported gender was not significant for WPV, being a nurse had higher odds of experiencing WPV (OR=1.95; 95% CI 1.46 to 2.59, p<0.001) than a physician (OR=1.70;

95% CI 1.33 to 2.18, p<0.001). In this study, most perpetrators were consumers (56%), followed by supervisors (16%) and colleagues (9%), or a combination of all (19%).

Violence perpetrated by colleagues (Type III-A) was reported by 15 studies, including seven for physicians (39,41,63–66,170), three studies for nurses (38,51,176), and five that included both professionals (27,35,42,58,61). Approximately 24% of violence was perpetrated by colleagues (Type III-A) among nurses and physicians. More perpetrators were men (63.5%) than women (23%), and some violence by colleagues was reported as perpetrated by both men and women (13.5%). Only one study (27) reported higher rates of bullying by women (37.9%) than men (10.5%) and by both genders (51.6%). In this study, most perpetrators (40.7%) were supervisors and senior colleagues (Type III-B). Victims were both physicians (53.1%) and nurses (53.6%) with similar intensity, but a higher number of women (n=195, 56.4%) were exposed to bullying than men (n=18, 36%). Additionally, those who experienced bullying had lower levels of psychological health status. Bullying from colleagues (26.4%) and patients/consumers (7.7%) was perceived as less harmful than bullying from supervisors (Type III-B), which was also less reported because of the fear of consequences.

Of the 34 studies reporting on GB-WPV (Table 3), 24 reported on the gender of the perpetrator for Type III-B (vertical) violence, which was more prevalent among physicians (51.5%) than nurses (16%). When it did occur among nurses, more men (77%) perpetuated Type III-B violence than women (18%) and both men and women (5%). Several studies highlighted physicians as perpetrators of WPV against nurses regardless of gender (6,49,51). Similarly, more men (67.5%) than women (24.2%) and both genders (8.2%) perpetuated Type III-B violence among physicians. In seven of ten studies (70%) for Type III-B violence among physicians, male supervisors and administrators perpetuated sexual harassment

(39,62–67). Four studies reported bullying (41,68,170) and emotional abuse (69), which was also perpetrated by men.

Medical residents appear to be particularly vulnerable to Type III-B violence, with more than 60% of studies (39,41,62,64,65,69,170,173) reporting this type of violence in medical residency programs. Furthermore, several studies highlighted that the perpetrator of sexual harassment was most often of the opposite sex (61,62,64). For instance, Freedman-Weiss et al. (64) reported that male residents experienced 65.9% of harassment from men compared to 81.8% from women. On the other hand, female residents reported experiencing more harassment from men (97.7%) compared to women (42.4%). In the same study, the main perpetrators for female resident victims were attending physicians (72.9%), followed by nurses (68.5%), senior colleagues (44.7%) and same-level residents (23.5%). Among male residents, nurses were the most common perpetrator of WPV (69%), followed by attending physicians (62%), senior colleagues (41.9%) and same-level residents (25.6%).

Healthcare professionals in lower hierarchical positions, such as nurses and residents, often contend with stressful conditions and managerial or administrative abuse and harassment, posing challenges to patient care, institutional integrity, and the healthcare system. These experiences also detrimentally impact the victims' health and career progression. For instance, Tekin and Bulut (49) found that Turkish nurses who experienced Type III-B violence reported feelings of anger, humiliation, confusion and sadness. Moreover, these experiences also led to strained relationships with others, decreased performance, and caused them to consider leaving the profession. Although this study did not specify the gender of the offender, women experienced significantly higher verbal abuse. The highest perpetuation for all forms of abuse, including verbal (85.7%), physical (46.4%) and sexual (94.4%), was from physicians. In these cases, gender and status within the organizational hierarchy played a critical role in perpetuating Type III-A and III-B WPV,

which requires serious attention from employers and health organizations to address GB-WPV through a gender-sensitive approach.

Table 3: Gender and the Perpetuation of Workplace Violence among Physicians and Nurses

S. No	Author/s	Population	Form of Violence	Type of Perpetrators			Gender of Perpetrators		
				Consumers	Horizontal	Vertical	Men %	Women %	Both Genders %
1.	Aghajanloo et al., 2011 (4)	Nursing students	Aggression	X			66.70	33.30	0.00
			Verbal abuse			X	49.95	50.05	0.00
2.	Chang et al., 2020 (6)	Nursing students	Sexual harassment	X		X	88.30	10.40	1.00
3.	Al-Ghabeesh & Qattom, 2019 (13)	Nurses	Bullying			X	52.50	17.50	30.00
4.	Chatziionnidis et al., 2018 (27)	Nurses and physicians	Bullying	X	X	X	10.50	37.90	51.60
5.	Ferri et al., 2020 (33)	Nurses	Verbal abuse	X			87.50	12.50	0.00
			Verbal & Physical violence	X			100.00	0.00	0.00
6.	Harthi et al., 2020 (35)	Nursing and medical personnel	Workplace Violence	X	X	X	39.42	20.33	40.25
7.	Lei et al., 2022 (38)	Nurses	Workplace Violence	X	X	X	83.00	17.00	0.00
8.	Menhaji et al., 2022 (39)	OB/Gyn trainees	Sexual Harassment	X	X	X	74.30	10.50	15.20
9.	Orlino et al., 2022 (41)	Medical trainees	Bullying	X	X	X	47.60	16.70	35.70
10.	Pinar et al., 2017 (42)	Health personnel	Physical	X			77.50	13.30	9.20
			Verbal abuse	X	X	X	72.60	18.00	9.40
			Sexual harassment	X		X	78.50	21.50	0.00
11.	Zampieron et al., 2010 (51)	Nursing personnel	Overall Aggression	X	X	X	79.60	20.40	0.00
			Verbal aggression	X	X	X	62.40	37.60	0.00
12.	Speroni et al., 2014 (52)	Nurses	Workplace Violence	X			62.40	27.50	10.10
13.	Wang et al., 2022 (53)	Nurses	Workplace Violence	X			62.40	37.60	0.00
14.	Weldehawaryat et al., 2020 (54)	Nurses	Workplace Violence	X		X	93.60	6.40	0.00
15.	Feng et al., 2022 (55)	General practitioners	Workplace Violence	X			79.49	20.51	0.00
16.	Özdamar Ünal et al., 2022 (56)	Physicians and others	Workplace Violence	X			61.90	38.10	0.00
17.	Turgut et al., 2021 (57)	Physicians	Workplace Violence	X			77.10	22.90	0.00
18.	Vezyridis et al., 2015 (58)	Nurses and physicians	Workplace Violence	X	X		80.60	19.40	0.00
19.	Elston & Gabe, 2016 (59)	General practitioners	Physical assault<br>Verbal abuse	X			82.00	18.00	0.00

20.	Oguz et al., 2020 (60)	Medical and nursing personnel	Physical violence	X			63.00	37.00	0.00
21.	Newman et al., 2011(61)	Midwives, nurses & Physician	Verbal abuse	X	X	X	22.00	55.00	23.00
			Sexual harassment				65.00	20.00	15.00
			Bullying	X	X	X	55.00	30.00	15.00
			Physical Attack	X	X	X	55.00	36.00	9.00
22.	Arnold et al., 2020 (62)	Physicians	Sexual Harassment (W)	X		X	99.00	0.00	1.00
			Sexual Harassment (M)			X	49.40	42.50	8.00
23.	Benzil et al., 2020 (63)	Surgeons	Sexual harassment		X	X	72.00	5.00	23.00
24.	Freedman-Weiss et al., 2020 (64)	Trainee residents	Harassment (W)		X	X	55.40	44.60	0.00
			Sexual Harassment (M)		X	X	30.30	69.70	0.00
25.	Nukala et al., 2020 (65)	Vascular trainees	Sexual harassment		X	X	60.00	40.00	0.00
26.	Smed et al., 2020 (66)	Vascular surgery	Sexual harassment		X	X	54.00	46.00	0.00
27.	Crebbin et al., 2015 (67)	Medical Personnel in Surgery	DBSH			X	79.00	21.00	0.00
			Sexual harassment			X	90.30	9.70	0.00
28.	Jain et al., 2019 (68)	Ophthalmologists	Bullying			X	79.50	20.50	0.00
29.	Lall et al., 2021(69)	E.M. residents	Emotional abuse	X		X	58.20	41.70	0.00
30.	Picakciefe et al., 2017 (70)	Health personnel	Mobbing			X	8.10	91.90	0.00
31.	Vorderwulbecke et al., 2015 (71)	Primary care physicians	Aggression	X			80.00	20.00	0.00
32.	DiFiori et al., 2023 (170)	Surgeons, fellow and residents	Bullying	X	X	X	67%	33%	0
33.	Janatolmakan et al., 2023	Hospital nurses	Physical Violence	X	X	X	89.2%	10.8%	0
			Verbal Violence				88%	12%	0
34.	Zachariadou et al., 2018 (190)	Medical, & Nursing personnel	Mobbing			X	19.80	69.10	11.00
**Average Perpetuation**							**65.07**	**28.18**	**6.74**

Discussion

Our examination explores the complexities of gender dynamics concerning both the perpetrator and the victims of workplace violence within the global healthcare community, mainly focusing on nurses and physicians. While 178 studies provided sex-segregated findings for workplace violence, only 34 (19%) of these reported the gender of the perpetrator, specifically for Type II and Type III violence. These findings provided insights into how gender and an individual's position within the organization create unique vulnerabilities to WPV. The consequences of such violence against health workers not only affect patient care but also have broader implications for healthcare organizations and workforce landscapes. In our review, men were found to be the primary instigators, accounting for 65% of incidences of WPV, while women were responsible for 28% of instances. Both men and women perpetrated the remaining 7% of incidents. Additionally, our analysis identified distinctive behaviour patterns among male and female offenders. Recognizing that each type of violence requires a different approach for its management and prevention, we will discuss the divergent behavioural patterns of men and women perpetrators of Type II and Type III violence. We will examine the underlying factors contributing to these differences and discuss the implications of adopting gender-sensitive approaches to prevent and manage GB-WPV.

Type II WPV- Client/Patient

Of the 34 studies that provided the gender of perpetrators for any type/s of violence, the majority (74%, n=25) reported on Type II WPV perpetrated by patients, their families, or visitors. In this context, male perpetrators were more prevalent, targeting both nurses (77.9%) and physicians (70%). The majority of studies that reported on Type II violence indicated a higher prevalence of various forms of violence against female nurses and physicians. The higher perpetration of WPV by men is linked to societal norms associating aggression and

dominance with masculinity. At the same time, violence against a feminized nursing workforce is normalized as part of the job (191).

These norms stem from social relations which dictate gender roles and responsibilities, and healthcare institutions are no exception to these forces. Type II violence typically targets healthcare providers in the performance of their professional duties and is characterized by acts of physical violence (4,33,42,59–61,164); verbal violence (4,33,42,51,61), and sexual harassment/ violence (6,39,42,61,62,165). Most of these studies reported a higher prevalence of WPV for women for all forms of violence (6,7,42,51,60,62,167,172,174); however, men were not immune to WPV, particularly physical violence (42,59,164,183). Three additional studies (35,57,187) reported a higher prevalence of both physical and verbal violence against male victims in the emergency department in Saudi Arabia, Turkey and China. In Turkey, male physicians experienced this violence at a rate of 62.4%, in contrast to their female counterparts at 37.6% (57). A similar pattern emerged in Saudi Arabia, with male physicians and nurses reporting a prevalence of 57.8%, while their female counterparts reported 42.8% (35). These two studies identified several factors for the high occurrence of WPV among male healthcare workers from patients and their relatives, including dissatisfaction with the treatment, long wait times and lack of staff (35,57), overcrowding and lack of security (35).

In contrast, women's experience of severe sexual harassment was associated with pregnancy, family responsibilities, and occupational segregation (61). Newman et al. (61) explained that occupational segregation, on the one hand, can create a vertical hierarchy where women are assigned to lower-level tasks (typically front-line care providers). On the other hand, it relates to the negative stereotypes that women are weak, indecisive and incompetent, which, in turn, sets the stage for higher rates of violence and discrimination. Considering the prevalence of Type II violence for both men and women linked to sex-

segregated responsibilities and societal structures. Jafree (192) calls on policymakers to ensure security and protection for the health workforce, particularly women; legislative reforms for healthcare governance and zero-tolerance policies for violence were also recommended. Other authors have also advocated for zero-tolerance policies and emphasized the need for a managerial approach that takes all complaints seriously, reports investigation outcomes and enforces sanctions to eliminate impunity (7). Collaborative community efforts are required to alter the patriarchal culture and reduce violence against women by creating awareness about the public role through various forums, such as the media (192).

Several contributing factors have been identified in the context of Type II WPV, such as noise levels, inadequate communication skills (72), perceived/actual staff incompetence or unsympathetic attitudes, dissatisfaction with service provision, prolonged wait times, and poor communication (51,193). These circumstances can escalate emotions and increase the likelihood of violent encounters. Furthermore, specific treatment specialties, such as emergency departments (33,73,189), psychiatric units (74,75), and geriatric care (24,74), have demonstrated a higher risk of Type II workplace violence. Factors specific to these settings include a lack of privacy and personal space, unrealistic expectations of clients, insufficient staffing and resources, poor staff skills mix, healthcare systems and processes not understood by clients, perceived favouritism, overcrowding in emergency departments, delays in providing analgesia, and inflexible visiting hours (193). These challenges, compounded by a shortage of skilled professionals, unclear expectations and communication, scheduling issues, and environmental stressors can generate increased stress and, thus, uncertainty. Addressing these factors constitutes the initial step in decreasing or eliminating the risk of violence for both men and women (194).

Both primary research and systematic reviews have acknowledged the difficulty associated with addressing multifactorial violence, given the diversity in population and

setting and the types/classifications of violence. For instance, a recent umbrella review examined 32 systematic reviews for WPV prevalence and characteristics. This comprehensive assessment reported that the overall prevalence from the meta-analysis of 11 reviews was 57.9%, ranging from 34.1% to 78.9% among healthcare providers and most affected were nurses working in psychiatric wards (195). This prevalence aligns with the findings of this review. Of note, the umbrella review did not provide prevalence based on gender and stated that the included reviews had reported variable results for men and women; however, it did underscore how gender imbalances in emergency departments could increase the risk of violence among women. Studies in our review recommended ensuring gender equality in the health workforce and leadership positions to reduce the prevalence of WPV among women (7,28,61).

Type III WPV-Worker-on-Worker

Type III-A (Horizontal or lateral) workplace violence perpetrated by one healthcare worker against another may stem from interpersonal conflicts, workplace stress (10), or other factors contributing to a hostile work environment. Among studies that provided data on Type III-A violence, most perpetrators were men (63.5%) compared to women (23%). Horizontal WPV was reported more frequently by physicians (39,41,63–67) than among nurses (38,51). The studies that sampled both nurses and physicians (27,35,42,58,61) also reported that men perpetuated all forms of violence in most cases for both male and female victims (35,42,61). In some instances, women experienced violence from both men and women (61).

Type III violence is rooted in cultural norms and societal expectations that allocate roles and responsibilities based on gender (61). This phenomenon transcends the household and is also seen in the workplace and healthcare institutions. These gendered roles and responsibilities often position men in leadership positions, while women are assigned roles

with less authority and responsibility, perpetuating discriminatory practices that negatively impact women (61,68). This dynamic prevails in both wealthy and lower- and middle-income countries. For instance, in Australia and New Zealand, women experienced significantly higher discrimination (31% vs. 8%) and sexual harassment (23% vs. 0.5%) than men, primarily due to family responsibilities, lack of mentorship and rigid promotion criteria (68). In Rwanda, women's experiences of childbearing and care, including managing pregnancy, motherhood and work, and the widespread negative stereotyping of women at work led to discrimination that co-occurred with sexual harassment within health workplaces (61). Additionally, relational and managerial issues, including organizational affairs within large, complex health organizations, shifting duties and cohabitation of various teams on the same unit, were identified as factors contributing to Type III-A violence in Italy (51).

Given the social reality of women's lives and career development, flexible human resource development and management policies empower women to balance their work and family responsibilities. Zampieroni et al.(51) recommend adopting realistic workloads and skill-mixed staffing, promoting gender equality in staff allocation, and participatory leadership to overcome relational conflict and managerial actions that enhance working conditions. Nurse managers must play the role of cultural gatekeepers, hold individuals accountable and foster staff empowerment; utilizing research-informed methods such as 'cognitive rehearsal and crucial conversations' (18) and conducting team-building workshops will assist in mitigating the impact of horizontal violence (19).

Type III-B (vertical) violence is primarily perpetrated by senior colleagues, supervisors, and administrative personnel occupying higher positions in the organizational hierarchy than the victim. Among the 34 studies, 66% reported perpetrators' gender for Type III-B violence; men perpetuated 77% among nurses and 67.5% among physicians. The causative factors for vertical violence included organizational structure, leadership and

administrative authorities, and power struggles in the health workplaces. These factors not only perpetuated WPV but also prohibited reporting of the instances due to fear of reprisal (27,61,64). Two prevalent forms of violence linked to hierarchical/ organizational structure were sexual harassment and bullying/mobbing. The majority of studies reporting Type III-B violence reported sexual violence from male supervisors and administrators (6,39,42,61–67), particularly in medical residency programs - placing these trainee residents in a vulnerable position (39,64,65). Additionally, vertical violence was the only type reported to be perpetuated by women at higher levels in the organizational hierarchy, particularly bullying (women: 37.9% vs. men: 10.5%) among nurses and physicians (27). Additionally, two studies reported higher rates of mobbing behaviours by women than men among healthcare professionals, including nurses and physicians (70,190).

Type III-B violence is emblematic of the hierarchical and inflexible organizational culture historically dominated by male medical professionals (196). This stemmed from beliefs and negative stereotypes, such as women being weak, unwilling to speak up, indecisive and incompetent (61). Such perceptions reinforce the structural power held by men, particularly with male managers and physicians. In addition, it reflects gender socialization whereby men are encouraged to exhibit assertive behaviours, and women are discouraged from assertiveness and confrontation, which can explain the perpetuation of domination among men and submissiveness among women (197). A qualitative study (198) from Estonia exposed this dynamic of domination and sexual harassment among nurses; it highlighted the association between power and the use of sexualized language. A nurse stated, “I am more disturbed by their patronizing behaviour"; the nurse characterizes physicians' attitude as: “I am a man, I am a physician, I can do and say whatever comes to my mind” (Nurse 18, p.30). Lamesoo (198) further explained that nurses placed themselves in the hospital hierarchy between physicians and patients and acknowledged that they could not

challenge a physician’s incivility. However, these nurses can easily ask patients to refrain from such behaviour without hesitation because patients have less power than nurses, and patients are expected to follow hospital rules (198) dictated by nurses. These instances explain organizational power as a protective factor for offenders. However, women's underrepresentation in positions of power places them in a vulnerable position.

A qualitative study in Uganda by Newman et al. (7) reported that women applicants in the workforce were promised or given jobs in exchange for sexual favours. The authors stated that "Sexual coercion started during recruitment of health workers and continued after hiring, perpetrated by men in hierarchically superior decision-making positions supervisors, senior managers (including human resources) or medical superintendents”(7). These severe human rights violations necessitate a transformation in the mindset of individuals in the workforce and a cultural shift at organizational levels to rectify the dominant, hierarchical and permissive environment (63). Ensuring gender equality at the upper echelons of healthcare organizations and in decision-making positions is crucial to establishing a secure and equitable environment for all, regardless of gender. A scoping review of three evidence-based guidelines and 33 systematic reviews on strategies to prevent and manage WPV in healthcare settings reported a correlation between strong leadership to cultivate and enforce a culture of inclusivity, support and respect as a prerequisite for successful prevention of WPV (199). Therefore, healthcare organizations' leadership must proactively seek organizational solutions to end gender-based WPV and prioritize gender equality and protecting employees' rights as part of their human resources for health (HRH) policy (7).

Sexual harassment in academia was found to be an issue across various contexts, particularly among women in medical residency programs. A study (76) in a U.S. medical college reported that one-third of respondents experienced sexual harassment, including medical students (51.7%), residents/fellows (31%) and faculty members (25%), which was

inversely proportional to their position in the program. Similarly, sexual harassment was more prevalent among women in vascular surgery in the U.S. (65), ophthalmology in Australia and New Zealand (77) and cardiothoracic surgery, reported by a global survey (26), and rates of sexual harassment in almost all contexts were higher among female trainees. In one instance, in the U.S., male (70%) and female (69%) residents (39) in obstetrics and gynecology residents experienced sexual harassment at similar levels (39). Additionally, one study in the U.S. with a large, representative sample (n=6000) from a national survey reported that higher women's representation within a specialty was associated with lower sexual harassment for both men and women from coworkers and patients (78). This observation held true in the Canadian context where reporting of sexual harassment incidents was low (2.9%) in a study with female participants constituting 53% of the sample (31). These women did report slightly higher rates of intimidation, harassment, and discrimination (IHD) based on gender (males 40.4%; females 48.0%). Hence, findings underscore the recurring recommendation of gender equality in the health workforce and the role of leadership in preventing Type II and Type III violence, including harassment.

Recognizing sexual harassment as a prevalent problem is the crucial initial step in formulating a successful strategy to prevent its occurrence (63,200); a comprehensive strategy should encompass a zero-tolerance statement across the specialty with a transparent and fair mechanism for reporting sexual harassment (63,76). Moreover, it is essential to provide trainees with both direct face-to-face and electronic routes for anonymous and confidential reporting to alleviate concerns related to personal reattribution and academic detriment (62,76). Standardized, transparent reporting mechanisms with well-delineated consequences for the offender must be established. Additionally, the institutions should ensure the availability of links to all the required resources is the first hit on online searches, displaying posters/presentations/ads (76).

Recognizing harassment as an institutional structural issue, senior leadership can have a protective role by serving as role models. A qualitative study conducted in Germany (194) representing women nurses (50%) and physicians (50%) explored preventive options for sexual harassment in academia. The findings revealed that leadership commitment and clear statements can significantly influence multiple levels by demonstrating openness to address taboo topics, raise awareness, and place the issue at the decision table. A participant stated, "A culture of political correctness is communicated from the top down, with the management committee and senior management acting as role models" (p.12). Another participant stated, "It is the senior staff that creates a team culture that should be supportive and transparent, with clear boundaries..... I have an open door and open eyes policy and try to initiate rituals that allow us to work together in the correct way" (p.12). While commitment and stated actions are essential, meaningful cultural change necessitates the consistent, active, structured, and continued engagement of all health workforce members, including students and trainees, staff and especially from senior leadership. Senior leadership must be actively engaged in this process, particularly male leaders. Therefore, engaging individuals at various levels in open, nonjudgmental conversations is paramount to breaking the silence (28) and ingraining these principles into the organizational culture.

The review categorized the studies into distinctive categories of Type II and Type III WPV. Limitations to this approach include the heterogeneity of the forms of violence reported by the included studies according to gender. While studies reported victims' exposure to Types II, and/or III, the gender of perpetrators in each case was not specified. As a result, we presented the prevalence of the various forms and identified the perpetrators' type for all the studies (178) in Table 2. The final set of studies (19%) that reported on the gender of the perpetrators was analyzed. Since fewer studies provided information about the gender of perpetrators across the types/forms of violence, future research must focus on conducting

and reporting gender-segregated findings for perpetrators that will strengthen recognition of the gender-based WPV and could lead to gender-sensitive strategies at the local and international levels. In addition, our review focused on gender as a binary based on the data provided. Considering this limitation, we recommend that future research include gender-diverse populations (LGBTQ).

Conclusion

The review revealed a higher prevalence of Type II and Type III WPV among women compared to men. In parallel, it was observed that men predominantly perpetrated all forms of violence against both men and women healthcare providers. Only Type III-B violence, including bullying/ mobbing, was occasionally perpetuated by women. Both Types II and III violence have roots in societal structures, and women were more frequently victimized. This increased victimization of women can be attributed to their lower status in society and in the healthcare settings that assign roles and responsibilities based on this status. Additionally, women's reproductive realities, including managing pregnancy, motherhood and work, and widespread negative stereotyping contributed to their vulnerability to gender-based WPV.

Conversely, men's domination in leadership, decision-making and supervisory positions in most contexts creates a hierarchical and permissive environment that perpetuates violence against women. Therefore, understanding gender implications concerning both the victim and perpetrator among the critical health workforce of nurses and physicians across the globe is essential. Healthcare organizations and professional stakeholders must seriously consider zero-tolerance policies, transparent mechanisms for handling violent incidents, and the provision of appropriate support to victims. These measures will empower individual professionals, enhance patient care, and positively impact healthcare institutions and society as a whole.

References

1. International Labour Organization, International Council of Nurses, World Health Organization, Public Services International. Framework guidelines for addressing workplace violence in the health sector. [Internet]. International Labour Office; 2002 [cited 2021 Apr 19]. Available from: http://www.icn.ch/images/stories/documents/pillars/sew/sew_framework_guidelines_for_addressing_workplace_violence.pdf

2. Cruz A, Klinger S. Gender-based violence in the world of work: Overview and selected annotated bibliography. 2011; Available from: https://www.ilo.org/wcmsp5/groups/public/---dgreports/---gender/documents/publication/wcms_155763.pdf

3. Lanctôt N, Guay S. The aftermath of workplace violence among healthcare workers: A systematic literature review of the consequences. Aggress Violent Behav. 2014 Sep;19(5):492–501.

4. Aghajanloo A, Nirumand-Zandi K, Safavi-Bayat Z, Alavi-Majd H. Clinical violence in nursing students. Iran J Nurs Midwifery Res. 2011;16(4):284–7.

5. Brown J, Drury L, Raub K, Levy B, Brantner P, Krivak TC, et al. Workplace Harassment and Discrimination in Gynecology: Results of the AAGL Member Survey. J Minim Invasive Gynecol. 2019;26(5):838–46.

6. Chang TS, Tzeng YL, Teng YK. Sexual Harassment Experiences, Knowledge, and Coping Behaviors of Nursing Students in Taiwan During Clinical Practicum. Int J Environ Res Public Health [Internet]. 2020;17(13). Available from: https://pubmed.ncbi.nlm.nih.gov/32630320/

7. Newman C, Nayebare A, Neema S, Agaba A, Akello LP. Uganda's response to sexual harassment in the public health sector: from "Dying Silently" to gender-transformational HRH policy. Hum Resour Health. 2021;19(1):59.

8. Public Services Health and Safety Association. Public Services Health and Safety Association. [cited 2023 Nov 14]. Public Services Health and Safety Association | Workplace Violence - Complying with the OHS Act. Available from: https://www.pshsa.ca/resources/workplace-violence-complying-with-the-ohs-act

9. Registered Nurses' Association of Ontario. Best Practice Guideline:Preventing Violence, Harassment and Bullying Against Health Workers [Internet]. RNAO; 2019 [cited 2023 Nov 14]. Available from: https://rnao.ca/bpg/guidelines/preventing-violence-harassment-and-bullying-against-health-workers

10. Jacobson AA, Colletti JE, Raukar NP. Horizontal Violence Toward Emergency Medicine Residents: Gender as a Risk Factor. West J Emerg Med Integrating Emerg Care Popul Health. 2022;23(5):633–6.

11. Workplace Violence: A Report to the Nation | Office of Justice Programs [Internet]. 2001 [cited 2023 Sep 27]. Available from: https://www.ojp.gov/ncjrs/virtual-library/abstracts/workplace-violence-report-nation

12. International Labour Organization. Statistics of work, employment and labour underutilization: 19th International Conference of Labour Statisticians [Internet]. Geneva; 2013. (ICLS/19/2013/2). Available from: https://www.ilo.org/wcmsp5/groups/public/---dgreports/---stat/documents/publication/wcms_220535.pdf

13. Al-Ghabeesh SH, Qattom H. Workplace bullying and its preventive measures and productivity among emergency department nurses. Bmc Health Serv Res. 2019;19(1):445.

14. George AS, McConville FE, de Vries S, Nigenda G, Sarfraz S, McIsaac M. Violence against female health workers is tip of iceberg of gender power imbalances. BMJ. 2020 Oct 27;m3546.

15. Arnetz JE, Hamblin L, Russell J, Upfal MJ, Luborsky M, Janisse J, et al. Preventing patient-to-worker violence in hospitals: outcome of a randomized controlled intervention. J Occup Environ Med. 2017 Jan;59(1):18–27.

16. Baig L, Tanzil S, Shaikh S, Hashmi I, Khan MA, Polkowski M. Effectiveness of training on de-escalation of violence and management of aggressive behavior faced by health care providers in a public sector hospital of Karachi. Pak J Med Sci. 2018;34(2):294–9.

17. Ceravolo DJ, Schwartz DG, Foltz-Ramos KM, Casenter J. Strengthening communication to overcome lateral violence. J Nurs Manag. 2012;20:599–606.

18. Sellers KF, Millenbach L, Ward K, Scribani M. The Degree of Horizontal Violence in RNs Practicing in New York State. J Nurs Adm. 2012;42(10):483–7.

19. Alameddine M, Mourad Y, Dimassi H. A National Study on Nurses' Exposure to Occupational Violence in Lebanon: Prevalence, Consequences and Associated Factors. Plos One. 2015;10(9):e0137105.

20. Al-Surimi K, Al Omar M, Alahmary K, Salam M. Prevalence of Workplace Bullying and Its Associated Factors at a Multi-Regional Saudi Arabian Hospital: A Cross-Sectional Study. Risk Manag Healthc Policy. 2020;13:1905–14.

21. Anand T, Grover S, Kumar R, Kumar M, Ingle GK. Workplace violence against resident doctors in a tertiary care hospital in Delhi. Natl Med J India. 2016;29(6):344–8.

22. Boafo IM, Hancock P, Gringart E. Sources, incidence and effects of non-physical workplace violence against nurses in Ghana. Nurs Open. 2016;3(2):99–109.

23. Brooks JT, Porter SE, Middleton KK, Carson EW, Ode GE. The Majority of Black Orthopaedic Surgeons Report Experiencing Racial Microaggressions During Their

Residency Training. Clin Orthop [Internet]. 2022; Available from: https://pubmed.ncbi.nlm.nih.gov/36342502/

24. Campbell JC, Messing JT, Kub J, Agnew J, Fitzgerald S, Fowler B, et al. Workplace Violence: Prevalence and Risk Factors in the Safe at Work Study. J Occup Environ Med. 2011 Jan;53(1):82–9.

25. Cavalcanti AL, Belo EDR, Marcolino E de C, Fernandes A, Cavalcanti YW, de Carvalho DF, et al. Occupational Violence against Brazilian Nurses. Iran J Public Health. 2018;47(11):1636–43.

26. Ceppa DP, Dolejs SC, Boden N, Phelan S, Yost KJ, Donington J, et al. Sexual Harassment and Cardiothoracic Surgery: #UsToo? Ann Thorac Surg. 2020;109(4):1283–8.

27. Chatziioannidis I, Bascialla FG, Chatzivalsama P, Vouzas F, Mitsiakos G. Prevalence, causes and mental health impact of workplace bullying in the Neonatal Intensive Care Unit environment. Bmj Open [Internet]. 2018;8(2). Available from: https://bmjopen.bmj.com/content/bmjopen/8/2/e018766.full.pdf

28. Chen W, Schilling BK, Bourne DA, Myers S, De La Cruz C. A Report of Gender Bias and Sexual Harassment in Current Plastic Surgery Training: A National Survey. Plast Reconstr Surg. 2021;147(6):1454–68.

29. Cheung T, Yip PSF. Workplace violence towards nurses in Hong Kong: prevalence and correlates. Bmc Public Health. 2017;17(1):196.

30. Cho H, Pavek K, Steege L. Workplace verbal abuse, nurse-reported quality of care and patient safety outcomes among early-career hospital nurses. J Nurs Manag. 2020;28(6):1250–8.

31. Crutcher RA, Szafran O, Woloschuk W, Chatur F, Hansen C. Family medicine graduates' perceptions of intimidation, harassment, and discrimination during residency training. Bmc Med Educ. 2011;11:88.

32. Dettmer S, Wenzel A, Trenkwalder T, Tiefenbacher C, Regitz-Zagrosek V. Gender and career in cardiology-a cross-sectional study. Herz. 2021;46(2):150–7.

33. Ferri P, Stifani S, Accoto A, Bonetti L, Rubbi I, Di Lorenzo R. VIOLENCE AGAINST NURSES IN THE TRIAGE AREA : A MIXED-METHODS STUDY. J Emerg Nurs. 2020;46(3):384–97.

34. Firenze A, Santangelo OE, Gianfredi V, Alagna E, Cedrone F, Provenzano S, et al. Violence on doctors. An observational study in Northern Italy. Med Lav. 2020;111(1):46–53.

35. Harthi M, Olayan M, Abugad H, Abdel Wahab M. Workplace violence among health-care workers in emergency departments of public hospitals in Dammam, Saudi Arabia. East Mediterr Health J Rev Sante Mediterr Orient Al-Majallah Al-Sihhiyah Li-Sharq Al-Mutawassit. 2020;26(12):1473–81.

36. Hu YY, Ellis RJ, Hewitt DB, Yang AD, Cheung EO, Moskowitz JT, et al. Discrimination, Abuse, Harassment, and Burnout in Surgical Residency Training. N Engl J Med. 2019;381(18):1741–52.

37. Kemper KJ, Schwartz A. Bullying, Discrimination, Sexual Harassment, and Physical Violence: Common and Associated With Burnout in Pediatric Residents. Acad Pediatr. 2020;20(7):991–7.

38. Lei Z, Yan S, Jiang H, Feng J, Han S, Herath C, et al. Prevalence and Risk Factors of Workplace Violence Against Emergency Department Nurses in China. Int J Public Health. 2022;67:1604912.

39. Menhaji K, Pan S, Hardart A. Sexual Harassment Prevalence Among OBGYN Trainees and Cultural Climate of their Training Programs: Result From a Nationwide Survey. J Surg Educ. 2022;79(5):1113–23.

40. Obeidat RF, Qan'ir Y, Turaani H. The relationship between perceived competence and perceived workplace bullying among registered nurses: A cross sectional survey. Int J Nurs Stud. 2018;88:71–8.

41. Orlino JP, Sura TA, Pei KY, Smeds MR. Bullying of vascular surgery trainees. J Vasc Surg. 2022;75(6):2065-2071.e3.

42. Pinar T, Acikel C, Pinar G, Karabulut E, Saygun M, Bariskin E, et al. Workplace Violence in the Health Sector in Turkey: A National Study. J Interpers Violence. 2017;32(15):2345–65.

43. Scruggs BA, Hock LE, Cabrera MT, Wang K, Oetting TA, Abramoff MD, et al. A U.S. Survey of Sexual Harassment in Ophthalmology Training Using a Novel Standardized Scale. J Acad Ophthalmol. 2020;12(1):e27–35.

44. Sharma G, Douglas PS, Hayes SN, Mehran R, Rzeszut A, Harrington RA, et al. Global Prevalence and Impact of Hostility, Discrimination, and Harassment in the Cardiology Workplace. J Am Coll Cardiol. 2021;77(19):2398–409.

45. Siller H, Tauber G, Komlenac N, Hochleitner M. Gender differences and similarities in medical students' experiences of mistreatment by various groups of perpetrators. Bmc Med Educ. 2017;17(1):134.

46. Simoes MRL, Barroso HH, de-Azevedo DS da S, Duarte ACM, Barbosa REC, Fonseca GC, et al. Workplace violence among municipal health care workers in Diamantina, Minas Gerais, Brazil, 2017. Rev Bras Med Trab Publicacao Of Assoc Nac Med Trab-ANAMT. 2020;18(1):82–90.

47. Subbiah IM, Markham MJ, Graff SL, Matt-Amaral LB, Close JL, Griffith KA, et al. Incidence, Nature, and Consequences of Oncologists' Experiences With Sexual Harassment. J Clin Oncol Off J Am Soc Clin Oncol. 2022;40(11):1186–95.

48. Swed S, Shoib S, Almoshantaf MB, Bohsas H, Hassan ASEM, Motawea KR, et al. A National Cross-Sectional Survey of Bullying in Syrian Graduate Medical Education. Front Public Health. 2022;10:916385.

49. Tekin YE, Bulut H. Verbal, Physical and Sexual Abuse Status Against Operating Room Nurses in Turkey. Sex Disabil. 2014;32(1):85–97.

50. Urnberg H, Gluschkoff K, Saukkonen P, Elovainio M, Vanska J, Heponiemi T. The association between stress attributed to information systems and the experience of workplace aggression: a cross-sectional survey study among Finnish physicians. Bmc Health Serv Res. 2022;22(1):724.

51. Zampieron A, Galeazzo M, Turra S, Buja A. Perceived aggression towards nurses: study in two Italian health institutions. J Clin Nurs. 2010;19(15–16):2329–41.

52. Speroni KG, Fitch T, Dawson E, Dugan L, Atherton M. INCIDENCE AND COST OF NURSE WORKPLACE VIOLENCE PERPETRATED BY HOSPITAL PATIENTS OR PATIENT VISITORS. J Emerg Nurs. 2014;40(3):218–28.

53. Wang LJ, Tanious A, Go C, Coleman DM, McKinley SK, Eagleton MJ, et al. Gender-based discrimination is prevalent in the integrated vascular trainee experience and serves as a predictor of burnout. J Vasc Surg. 2020;71(1):220–7.

54. Weldehawaryat HN, Weldehawariat FG, Negash FG. Prevalence of Workplace Violence and Associated Factors Against Nurses Working in Public Health Facilities in Southern Ethiopia. Risk Manag Healthc Policy. 2020;13:1869–77.

55. Feng J, Lei Z, Yan S, Jiang H, Shen X, Zheng Y, et al. Prevalence and associated factors for workplace violence among general practitioners in China: a national cross-sectional study. Hum Resour Health. 2022;20(1):42.

56. Ozdamar Unal G, Iscan G, Unal O. The occurrence and consequences of violence against healthcare workers in Turkey: before and during the COVID-19 pandemic. Fam Pract. 2022;39(6):1001–8.

57. Turgut K, Yavuz E, Yildiz MK, Poyraz MK. Violence toward emergency physicians: A prospective-descriptive study. World J Emerg Med. 2021;12(2):111–6.

58. Vezyridis P, Samoutis A, Mavrikiou PM. Workplace violence against clinicians in Cypriot emergency departments: a national questionnaire survey. J Clin Nurs. 2015;24(9–10):1210–22.

59. Elston MA, Gabe J. Violence in general practice: a gendered risk? Sociol Health Illn. 2016;38(3):426–41.

60. Oguz M, Sayin E, Gurses D. Violence against health employees in a child health and diseases clinic: A tertiary-level hospital example. Turk Pediatri Arsivi. 2020;55(2):117–23.

61. Newman C, de Vries DH, d'Arc Kanakuze J, Ngendahimana G. Workplace violence and gender discrimination in Rwanda's health workforce: Increasing safety and gender equality. Hum Resour Health. 2011 Jul 19;9(1):19.

62. Arnold LF, Zargham SR, Gordon CE, McKinley WI, Bruenderman EH, Weaver JL, et al. Sexual Harassment during Residency Training: A Cross-Sectional Analysis. Am Surg. 2020;86(1):65–72.

63. Benzil DL, Muraszko KM, Soni P, Air EL, Orrico KO, Rutka JT. Toward an understanding of sexual harassment in neurosurgery. J Neurosurg. 2020;1–10.

64. Freedman-Weiss MR, Chiu AS, Heller DR, Cutler AS, Longo WE, Ahuja N, et al. Understanding the Barriers to Reporting Sexual Harassment in Surgical Training. Ann Surg. 2020;271(4):608–13.

65. Nukala M, Freedman-Weiss M, Yoo P, Smeds MR. Sexual Harassment in Vascular Surgery Training Programs. Ann Vasc Surg. 2020;62:92–7.

66. Smeds MR, Aulivola B. Gender disparity and sexual harassment in vascular surgery practices. J Vasc Surg. 2020;72(2):692–9.

67. Crebbin W, Campbell G, Hillis DA, Watters DA. Prevalence of bullying, discrimination and sexual harassment in surgery in Australasia. Anz J Surg. 2015;85(12):905–9.

68. Jain NS, Kersten HM, Watson SL, Danesh-Meyer HV. Gender differences in Australasian ophthalmologists' experiences of the workplace. Clin Experiment Ophthalmol. 2019;47(6):706–12.

69. Lall MD, Bilimoria KY, Lu DW, Zhan T, Barton MA, Hu YY, et al. Prevalence of Discrimination, Abuse, and Harassment in Emergency Medicine Residency Training in the US. Jama Netw Open. 2021;4(8):e2121706.

70. Picakciefe M, Acar G, Colak Z, Kilic I. The Relationship Between Sociodemographic Characteristics, Work Conditions, and Level of "Mobbing" of Health Workers in Primary Health Care. J Interpers Violence. 2017;32(3):373–98.

71. Vorderwulbecke F, Feistle M, Mehring M, Schneider A, Linde K. Aggression and violence against primary care physicians-a nationwide questionnaire survey. Dtsch Arzteblatt Int. 2015;112(10):159–65.

72. Xie Z, Li J, Chen Y, Cui K. The effects of patients initiated aggression on Chinese medical students' career planning. Bmc Health Serv Res. 2017;17(1):849.

73. Al-Maskari SA, Al-Busaidi IS, Al-Maskari MA. Workplace violence against emergency department nurses in Oman: a cross-sectional multi-institutional study. Int Nurs Rev. 2020;67(2):249–57.

74. Ferri P, Silvestri M, Artoni C, Di Lorenzo R. Workplace violence in different settings and among various health professionals in an Italian general hospital: a cross-sectional study. Psychol Res Behav Manag. 2016 Sep 23;9:263–75.

75. James BO, Isa EW, Oud N. Patient aggression in psychiatric services: the experience of a sample of nurses at two psychiatric facilities in Nigeria. Afr J Psychiatry. 2011;14(2):130–3.

76. Hsiao CJ, Akhavan NN, Singh Ospina N, Yagnik KJ, Neilan P, Hahn P, et al. Sexual Harassment Experiences Across the Academic Medicine Hierarchy. Cureus. 2021;13(2):e13508.

77. Meyer JA, Troutbeck R, Oliver GF, Gordon LK, Danesh-Meyer HV. Bullying, harassment and sexual discrimination among ophthalmologists in Australia and New Zealand. Clin Experiment Ophthalmol. 2021;49(1):15–24.

78. Hu X, Conrad S, Dill M. Specialty Gender Composition and Prevalence of Physician Harassment. J Womens Health 2002 [Internet]. 2022; Available from: http://ovidsp.ovid.com/ovidweb.cgi?T=JS&PAGE=reference&D=medp&NEWS=N&AN=36576791

79. Forrest LE, Herath PM, McRae IS, Parker RM. A national survey of general practitioners' experiences of patient-initiated aggression in Australia. Med J Aust. 2011;194(11):605–8.

80. Abed M, Morris E, Sobers-Grannum N. Workplace violence against medical staff in healthcare facilities in Barbados. Occup Med. 2016 Oct;66(7):580–3.

81. Vyas S, Saini RS, Semwal J, Sharma N, Chaturvedi M, Ansari MWF. Are Health Caregivers safe from workplace violence? A cross sectional study on workplace safety from Tertiary Care Hospital of Uttarakhand. Indian J Community Health. 2022;34(4):469–76.

82. Giglio V, Schneider P, Bond Z, Madden K, McKay P, Bozzo A, et al. Prevalence of gender-based and sexual harassment within orthopedic surgery in Canada. Can J Surg J Can Chir. 2022;65(1):E45–51.

83. Abrams KM, Robinson GE. Stalking by Patients: Doctors' Experiences in a Canadian Urban Area. J Nerv Ment Dis. 2011 Oct;199(10):738–43.

84. Afkhamzadeh A, Amjad Mohamadi B, Moloudi B, Safari H, Bakhtiar P. Workplace violence against physicians and medical students in west part of Iran. Int J Hum Rights Healthc. 2019;12(2):116–23.

85. Alhamad R, Suleiman A, Bsisu I, Santarisi A, Al Owaidat A, Sabri A, et al. Violence against physicians in Jordan: An analytical cross-sectional study. Plos One [Internet]. 2021;16(1). Available from: ://WOS:000613081500066https://storage.googleapis.com/plos-corpus-prod/10.1371/journal.pone.0245192/

86. Al-Shamlan NA, Jayaseeli N, Al-Shawi MM, Al-Joudi AS. Are nurses verbally abused? A cross-sectional study of nurses at a university hospital, Eastern Province, Saudi Arabia. J Fam Community Med. 2017;24(3):173–80.

87. Balch Samora J, Van Heest A, Weber K, Ross W, Huff T, Carter C. Harassment, Discrimination, and Bullying in Orthopaedics: A Work Environment and Culture Survey. J Am Acad Orthop Surg. 2020;28(24):e1097–104.

88. Bhandari S, Jha P, Cooper C, Slawski B. Gender-Based Discrimination and Sexual Harassment Among Academic Internal Medicine Hospitalists. J Hosp Med. 2021;16(2):84–9.

89. Jaradat Y, Nielsen MB, Kristensen P, Nijem K, Bjertness E, Stigum H, et al. Workplace aggression, psychological distress, and job satisfaction among Palestinian nurses: A cross-sectional study. Appl Nurs Res. 2016;32:190–8.

90. Cashmore AW, Indig D, Hampton SE, Hegney DG, Jalaludin BB. Workplace violence in a large correctional health service in New South Wales, Australia: a retrospective review of incident management records. Bmc Health Serv Res. 2012;12:245.

91. Chen X, Lv M, Wang M, Wang X, Liu J, Zheng N, et al. Incidence and risk factors of workplace violence against nurses in a Chinese top-level teaching hospital: A cross-sectional study. Appl Nurs Res ANR. 2018;40:122–8.

92. Cheng S, Dawson J, Thamby J, Liaw WR, King EB. How do aggression source, employee characteristics and organisational response impact the relationship between workplace aggression and work and health outcomes in healthcare employees? A cross-sectional analysis of the National Health Service staff surve. Bmj Open. 2020;10(8):e035957.

93. Dal Pai D, Lautert L, de Souza SBC, Marziale MHP, Tavares JP. Violence, burnout and minor psychiatric disorders in hospital work. Rev Esc Enferm USP. 2015;49(3):457–64.

94. Dehghan-Chaloshtari S, Ghodousi A. Factors and Characteristics of Workplace Violence Against Nurses: A Study in Iran. J Interpers Violence. 2020;35(1–2):496–509.

95. Demeur V, Devos S, Jans E, Schoenmakers B. Aggression towards the GP: can we profile the GP-victim? A cross-section survey among GPs. BJGP Open. 2018;2(3):bjgpopen18X101604.

96. Difazio RL, Vessey JA, Buchko OA, Chetverikov DV, Sarkisova VA, Serebrennikova NV. The incidence and outcomes of nurse bullying in the Russian Federation. Int Nurs Rev. 2019;66(1):94–103.

97. Fitzgerald CA, Smith RN, Luo-Owen X, Turay D, Ferrada P, Han J, et al. Screening for Harassment, Abuse, and Discrimination among Surgery Residents: An EAST Multicenter Trial. Am Surg. 2019;85(5):456–61.

98. Fnais N, al-Nasser M, Zamakhshary M, Abuznadah W, Dhukair SA, Saadeh M, et al. Prevalence of harassment and discrimination among residents in three training hospitals in Saudi Arabia. Ann Saudi Med. 2013;33(2):134–9.

99. Fute M, Mengesha ZB, Wakgari N, Tessema GA. High prevalence of workplace violence among nurses working at public health facilities in Southern Ethiopia. Bmc Nurs. 2015;14:9.

100. Hills DJ, Joyce CM, Humphreys JS. A national study of workplace aggression in Australian clinical medical practice. Med J Aust. 2012;197(6):336–40.

101. Honarvar B, Ghazanfari N, Raeisi Shahraki H, Rostami S, Lankarani KB. Violence against Nurses: A Neglected and Health-threatening Epidemic in the University Affiliated Public Hospitals in Shiraz, Iran. Int J Occup Environ Med. 2019;10(3):111–23.

102. Kisiel MA, Kuhner S, Stolare K, Lampa E, Wohlin M, Johnston N, et al. Medical students' self-reported gender discrimination and sexual harassment over time. Bmc Med Educ. 2020;20(1):503.

103. Lafta RK, Falah N. Violence against health-care workers in a conflict affected city. Med Confl Surviv. 2019;35(1):65–79.

104. Li M, Liu J, Zheng J, Liu K, Wang J, Amy Miner R, et al. The relationship of workplace violence and nurse outcomes: Gender difference study on a propensity score matched sample. J Adv Nurs. 2020;76(2):600–10.

105. Li SF, Grant K, Bhoj T, Lent G, Garrick JF, Greenwald P, et al. Resident experience of abuse and harassment in emergency medicine: ten years later. J Emerg Med. 2010;38(2):248–52.

106. Lu DW, Lall MD, Mitzman J, Heron S, Pierce A, Hartman ND, et al. #MeToo in EM: A Multicenter Survey of Academic Emergency Medicine Faculty on Their Experiences with Gender Discrimination and Sexual Harassment. West J Emerg Med. 2020;21(2):252–60.

107. Lucas-Guerrero V, Pascua-Sole M, Ramos Rodriguez JL, Trinidad Borras A, Gonzalez de Pedro C, Jover Navalon JM, et al. Burnout in General Surgery Residents. Survey From the Spanish Association of Surgeons. Desgaste Prof O Burn En Los Resid Cirugia Gen Encuesta Asoc Espanola Cir. 2020;98(8):442–9.

108. Margavi MK, Bagheri-Nesami M, Mousavinasab N, Lolaty HA. Frequency of violence against nurses and its related factors during cardiopulmonary resuscitation in emergency wards. J Nurs Midwifery Sci. 2020;7(4):262–8.

109. McKinley SK, Wang LJ, Gartland RM, Westfal ML, Costantino CL, Schwartz D, et al. "Yes, I'm the Doctor": One Department's Approach to Assessing and Addressing Gender-Based Discrimination in the Modern Medical Training Era. Acad Med J Assoc Am Med Coll. 2019;94(11):1691–8.

110. Mirza NM, Amjad AI, Bhatti ABH, tuz Zahra Mirza F, Shaikh KS, Kiani J, et al. Violence and abuse faced by junior physicians in the emergency department from patients and their caretakers: a nationwide study from Pakistan. J Emerg Med. 2012;42(6):727–33.

111. Moman RN, Maher DP, Hooten WM. Workplace Violence in the Setting of Pain Management. Mayo Clin Proc Innov Qual Outcomes. 2020;4(2):211–5.

112. Moylan LB, Cullinan MB, Kimpel JE. Differences in Male and Female Nurses’ Responses to Physical Assault by Psychiatric Patients. J Psychosoc Nurs Ment Health Serv. 2014;52(12):36–42.

113. Nieto-Gutierrez W, Toro-Huamanchumo CJ, Taype-Rondan A, Timana-Ruiz R, Alva Diaz C, Jumpa-Armas D, et al. Workplace violence by specialty among Peruvian medical residents. Plos One. 2018;13(11):e0207769.

114. Park SH, Choi EH. The Cycle of Verbal Violence Among Nurse Colleagues in South Korea. J Interpers Violence. 2020;886260520945680.

115. Pol A, Carter M, Bouchoucha S. Violence and aggression in the intensive care unit: What is the impact of Australian National Emergency Access Target? Aust Crit Care Off J Confed Aust Crit Care Nurses. 2019;32(6):502–8.

116. Prajapati R, Baral B, Karki KB, Neupane M. Perception of security by health workforce at workplace in Nepal. J Nepal Health Res Counc. 2013;11(24):153–7.

117. Rosta J, Aasland OG. Perceived bullying among Norwegian doctors in 1993, 2004 and 2014-2015: a study based on cross-sectional and repeated surveys. Bmj Open [Internet]. 2018;8(2). Available from: ://WOS:000433129800193

118. Rouse LP, Gallagher-Garza S, Gebhard RE, Harrison SL, Wallace LS. Workplace Bullying Among Family Physicians: A Gender Focused Study. J Womens Health. 2016;25(9):882–8.

119. Sachdeva S, Jamshed N, Aggarwal P, Kashyap SR. Perception of Workplace Violence in the Emergency Department. J Emerg Trauma Shock. 2019;12(3):179–84.

120. Sakellaropoulos A, Pires J, Estes D, Jasinski D. Workplace aggression: assessment of prevalence in the field of nurse anesthesia. AANA J. 2011;79(4 Suppl):S51-7.

121. Tian Y, Yue Y, Wang J, Luo T, Li Y, Zhou J. Workplace violence against hospital healthcare workers in China: a national WeChat-based survey. Bmc Public Health. 2020;20(1):582.

122. Vargas EA, Brassel ST, Cortina LM, Settles IH, Johnson TRB, Jagsi R. #MedToo: A Large-Scale Examination of the Incidence and Impact of Sexual Harassment of Physicians and Other Faculty at an Academic Medical Center. J Womens Health. 2020;29(1):13–20.

123. Viottini E, Politano G, Fornero G, Pavanelli PL, Borelli P, Bonaudo M, et al. Determinants of aggression against all health care workers in a large-sized university hospital. Bmc Health Serv Res. 2020;20(1):215.

124. Williams KE, Baskin ML, Brito AL, Bae S, Willett LL. Supporting Trainees by Addressing Inappropriate Behaviors by Patients. South Med J. 2021;114(2):111–5.

125. Camm CF, Joshi A, Moore A, Sinclair HC, Westwood M, Greenwood JP, et al. Bullying in UK cardiology: a systemic problem requiring systemic solutions. Heart Br Card Soc. 2022;108(3):212–8.

126. Rowe SG, Stewart MT, Van Horne S, Pierre C, Wang H, Manukyan M, et al. Mistreatment Experiences, Protective Workplace Systems, and Occupational Distress in Physicians. Jama Netw Open. 2022;5(5):e2210768–e2210768.

127. Vidal-Alves MJ, Pina D, Puente-Lopez E, Luna-Maldonado A, Luna Ruiz-Cabello A, Magalhaes T, et al. Tough Love Lessons: Lateral Violence among Hospital Nurses. Int J Environ Res Public Health [Internet]. 2021;18(17). Available from: https://www.ncbi.nlm.nih.gov/pmc/articles/PMC8431196/

128. Kibunja BK, Musembi HM, Kimani RW, Gatimu SM. Prevalence and Effect of Workplace Violence against Emergency Nurses at a Tertiary Hospital in Kenya: A Cross-Sectional Study. Saf Health Work. 2021;12(2):249–54.

129. Sabak M, Al-Hadidi A, Oktay MM, Al B, Kazaz T, Kowalenko T, et al. Workplace Violence in Emergency Departments in Turkey. Avicenna J Med. 2021;11(3):111–7.

130. Gadjradj PS, Ghobrial JB, Booi SA, de Rooij JD, Harhangi BS. Mistreatment, discrimination and burn-out in Neurosurgery. Clin Neurol Neurosurg. 2021;202:106517.

131. Albuainain HM, Alqurashi MM, Alsadery HA, Alghamdi TA, Alghamdi AA, Alghamdi RA, et al. Workplace bullying in surgical environments in Saudi Arabia: A multiregional cross-sectional study. J Fam Community Med. 2022;29(2):125–31.

132. Noland ST, Taipale H, Mahmood JI, Tyssen R. Analysis of Career Stage, Gender, and Personality and Workplace Violence in a 20-Year Nationwide Cohort of Physicians in Norway. Jama Netw Open. 2021;4(6):e2114749.

133. Papantoniou P. Comparative analysis of sexual harassment between male and female nurses: A cross-sectional study in Greece. J Nurs Manag. 2022;30(6):1470–81.

134. Kowalczuk K, Krajewska-Kulak E. Patient aggression towards different professional groups of healthcare workers. Ann Agric Environ Med AAEM. 2017;24(1):113–6.

135. Notaro E, Pascoe VL, Hippe DS, Lachance K, Shinohara MM, DeNiro KL. Prevalence of sexual harassment and sexual assault from patient to provider among women in dermatology and across specialties. Int J Womens Dermatol. 2021;7(3):270–5.

136. Schlick CJR, Ellis RJ, Etkin CD, Greenberg CC, Greenberg JA, Turner PL, et al. Experiences of Gender Discrimination and Sexual Harassment Among Residents in General Surgery Programs Across the US. JAMA Surg. 2021;156(10):942–52.

137. Hunter EJ, Eades CE, Evans JMM. Violence experienced by undergraduate nursing students during clinical placements: An online survey at a Scottish University. Nurse Educ Pract. 2022;61:103323.

138. Ferrara P, Terzoni S, Destrebecq A, Ruta F, Sala E, Formenti P, et al. Violence and unsafety in Italian hospitals: Experience and perceptions of nursing students. Work Read Mass. 2022;73(1):211–7.

139. Pendleton AA, McKinley SK, Pendleton VE, Ott QC, Petrusa ER, Srivastava SD, et al. A multi-institutional study of patient-derived gender-based discrimination experienced by resident physicians. Am J Surg. 2021;221(2):309–14.

140. Snavely C, Romeo M, Ciardiello A, Mojica M. The pandemic of workplace violence: the gendered experience of emergency medicine trainees. AEM Educ Train. 2021;5(3):e10630.

141. Hock LE, Barlow PB, Scruggs BA, Oetting TA, Martinez DA, Abramoff MD, et al. Tools for Responding to Patient-Initiated Verbal Sexual Harassment: A Workshop for Trainees and Faculty. MedEdPORTAL J Teach Learn Resour. 2021;17:11096.

142. Rodriguez-Acosta RL, Myers DJ, Richardson DB, Lipscomb HJ, Chen JC, Dement JM. Physical assault among nursing staff employed in acute care. Work Read Mass. 2010;35(2):191–200.

143. Hahn S, Muller M, Hantikainen V, Kok G, Dassen T, Halfens RJG. Risk factors associated with patient and visitor violence in general hospitals: Results of a multiple regression analysis. Int J Nurs Stud. 2013;50(3):374–85.

144. Wang T, Abrantes ACM, Liu Y. Intensive care units nurses' burnout, organizational commitment, turnover intention and hospital workplace violence: A cross-sectional study. Nurs Open. 2022;(2):t.

145. Wright W, Khatri N. Bullying among nursing staff: Relationship with psychological/behavioral responses of nurses and medical errors. Health Care Manage Rev. 2015;40(2):139–47.

146. Bambi S, Becattini G, Giusti GD, Mezzetti A, Guazzini A, Lumini E. Lateral hostilities among nurses employed in intensive care units, emergency departments, operating rooms, and emergency medical services. A national survey in Italy. Dimens Crit Care Nurs DCCN. 2014;33(6):347–54.

147. Koukia E, Mangoulia P, Papageorgiou DE, Gonis N, Katostaras T. Violence against health care staff by peers and managers in a general hospital in Greece: a questionnaire-based study. Aust J Adv Nurs. 2014;31(4):45–56.

148. Al-Omari H. Physical and verbal workplace violence against nurses in Jordan. Int Nurs Rev. 2015;62(1):111–8.

149. Esmaeilpour M, Salsali M, Ahmadi F. Workplace violence against Iranian nurses working in emergency departments. Int Nurs Rev. 2011;58(1):130–7.

150. Joa TS, Morken T. Violence towards personnel in out-of-hours primary care: a cross-sectional study. Scand J Prim Health Care. 2012;30(1):55–60.

151. Serafin LI, Czarkowska-Paczek B. Prevalence of bullying in the nursing workplace and determinant factors: a nationwide cross-sectional Polish study survey. Bmj Open. 2019;9(12):e033819.

152. Kelly EL, Subica AM, Fulginiti A, Brekke JS, Novaco RW. A cross-sectional survey of factors related to inpatient assault of staff in a forensic psychiatric hospital. J Adv Nurs. 2015;71(5):1110–22.

153. Fafliora E, Bampalis VG, Zarlas G, Sturaitis P, Lianas D, Mantzouranis G. Workplace violence against nurses in three different Greek healthcare settings. Work Read Mass. 2015;53(3):551–60.

154. Askew DA, Schluter PJ, Dick ML, Rego PM, Turner C, Wilkinson D. Bullying in the Australian medical workforce: cross-sectional data from an Australian e-Cohort study. Aust Health Rev Publ Aust Hosp Assoc. 2012;36(2):197–204.

155. Lindquist B, Feltes M, Niknam K, Koval KW, Ohn H, Newberry J, et al. Experiences of Workplace Violence Among Healthcare Providers in Myanmar: A Cross-sectional Survey Study. Cureus. 2020;12(4):e7549.

156. Yohe N, Swiggett SJ, Razi A, Bowman JR, Watson SL, Pearson JM, et al. Acute Workplace Hazards in Orthopedic Surgery: Resident Survey Regarding Splash and Workplace Violence Events. J Surg Educ. 2020;77(6):1638–45.

157. Farid H, Stack-Dunniber H, Molina R, Nosal C, Mendiola M, Hacker M. Discrimination, Microaggressions, and Perceptions of Institutional Response in an Academic Obstetrics and Gynecology Department. Cureus. 2021;13(6):e15993.

158. Vingers JA. How Gender Affects Experiences With Bullying in Prelicensure BSN Programs: A Pilot Study. Nurs Educ Perspect. 2018;39(4):230–2.

159. Dafny HA, Beccaria G. I do not even tell my partner: Nurses' perceptions of verbal and physical violence against nurses working in a regional hospital. J Clin Nurs. 2020;29(17–18):3336–48.

160. Ko M, Dorri A. Primary Care Clinician and Clinic Director Experiences of Professional Bias, Harassment, and Discrimination in an Underserved Agricultural Region of California. Jama Netw Open. 2019;2(10):e1913535.

161. Neiterman E, Bourgeault IL. The shield of professional status: Comparing internationally educated nurses' and international medical graduates' experiences of discrimination. Health Lond Engl 1997. 2015;19(6):615–34.

162. Al Khatib O, Taha H, Al Omari L, Al-Sabbagh MQ, Al-Ani A, Massad F, et al. Workplace Violence against Health Care Providers in Emergency Departments of Public Hospitals in Jordan: A Cross-Sectional Study. Int J Environ Res Public Health. 2023;20(4).

163. Al-Wathinani AM, Barten DG, Al-Balawi H, Al-Aqeel S, Al-Dulijan N, Al-Ghamdi K, et al. Reducing Violence in Riyadh's Emergency Departments: The Critical Role of Healthcare Providers. Healthc Basel Switz. 2023;11(6).

164. Alhassan A.K., Alsaqat R.T., Al Sweleh F.S. Physical workplace violence in the health sector in Saudi Arabia. Med U S. 2023;102(29):E34094.

165. AlHassan AK, AlSaqat RT, Al Sweleh FS. Sexual workplace violence in the health sector in Saudi Arabia: a cross sectional study. BMC Health Serv Res. 2023;23(1):1065.

166. Banga A, Mautong H, Alamoudi R, Faisal UH, Bhatt G, Amal T, et al. ViSHWaS: Violence Study of Healthcare Workers and Systems-a global survey. BMJ Glob Health. 2023;8(9).

167. Barequet IS, Rosenblatt A, Schaap Fogler M, Pedut-Kloizman T, Gaton D, Loewenstein A, et al. Gender related trends among Israeli ophthalmologists professional career and personal life performance. Eye Lond Engl. 2023;37(16):3496–501.

168. Bekalu YE, Wudu MA. Prevalence of Workplace Violence and Associated Factors Against Nurses Working in Public Hospitals in Northeastern Ethiopia, 2022. SAGE Open Nurs. 2023;9(101724853):23779608231171776.

169. Crombie KE, Crombie KD, Salie M, Seedat S. Medical Students' Experiences of Mistreatment by Clinicians and Academics at a South African University. Teach Learn Med. 2024 Jan;36(1):13–22.

170. DiFiori MM, Gupta SS, Cannada LK, Pei KY, Stamm MA, Mulcahey MK. Bullying in Orthopaedic Surgery: A Survey of US Orthopaedic Trainees and Attending Surgeons. J Am Acad Orthop Surg Glob Res Rev. 2023;7(5).

171. Dominguez LC, Torregrosa L, Cuevas L, Pena L, Sanchez S, Pedraza M, et al. Workplace bullying and sexual harassment among general surgery residents in Colombia. Acoso Labor Sex En Resid Cirugia Gen En Colomb. 2023;43(2):252–60.

172. Ebrahim FA, Shah J, Sharma K, Kunyiha N, Korom R, Ali SK. Discrimination and Abuse Among Healthcare Workers from Patients and their Relatives at a Tertiary Hospital in Kenya. Behav Med Wash DC. 2023;(beh, 8804264):1–8.

173. Forsythe RO, Van Herzeele I, Zayed H, Argyriou A, Stavroulakis K, Saratzis A. International Cross-Sectional Survey of Bullying, Undermining, and Harassment in the Vascular Workplace. "Research Collaborative in Peripheral Artery Disease" (RCPAD) Collaborative, editor. Eur J Vasc Endovasc Surg Off J Eur Soc Vasc Surg. 2023;65(5):748–55.

174. Grover A, Santen S, Hemphill R, Goldberg S, Savage N. Patient and Family Mistreatment of Residents in the Surgical Learning Environment. Am Surg. 2023;89(4):533–8.

175. Ioanidis K, Naismith K, Dzioba A, MacNeil SD, Paradis J, Nayan S, et al. Canadian Women in Otolaryngology—Head and Neck Surgery part 1: the relationship of gender

identity to career trajectory and experiences of harassment. J Otolaryngol -- Head Neck Surg. 2023 Apr 24;52(1):1–8.

176. Janatolmakan M, Abdi A, Rezaeian S, Framarzi Nasab N, Khatony A. Violence against Emergency Nurses in Kermanshah-Iran: Prevalence and Associated Factors. Nurs Res Pract. 2023;2023(101561211):9362977.

177. Meese KA, Boitet LM, Schmidt JJ, Borkowski N, Sweeney KL. Exploring National Trends and Organizational Predictors of Violence and Mistreatment From Patients and Visitors. J Healthc Manag. 2024 Feb 1;69(1):29–44.

178. Nam CS, Gupta P, Stroumsa D, Byrd KM, Lee KT, Goh M, et al. Self-reported Patient-perpetrated Sexual Harassment and Subsequent Reporting Among Health Care Clinicians in Urology and Obstetrics-Gynecology: A Cohort Survey. Urology. 2023;182(wsy, 0366151):239–43.

179. Parodi JB, Burgos LM, Garcia-Zamora S, Liblik K, Pulido L, Gupta S, et al. Gender differences in workplace violence against physicians and nurses in Latin America: a survey from the Interamerican Society of Cardiology. Public Health Elsevier. 2023 Dec;225:127–32.

180. Rashid S, Ullah A, Satti DI, Malik J, Iqbal H, Mehmoodi A, et al. Bullying in Cardiology: Pakistan's Perspective. Curr Probl Cardiol. 2023;48(7):101691.

181. Ryan S, Bozoghlian M, Lawler E, Patterson B. Patient Initiated Discrimination and Harassment-A Descriptive Survey of Experiences Within a Single Academic Department. Iowa Orthop J. 2023;43(1):1–6.

182. Santosa KB, Hayward L, Matusko N, Kubiak CA, Strong AL, Waljee JF, et al. Attributions and perpetrators of incivility in academic surgery. Glob Surg Educ J Assoc Surg Educ. 2023;2(1):56.

183. Shahjalal Md, Parvez Mosharaf Md, Mahumud RA. Effect of workplace violence on health workers injuries and workplace absenteeism in Bangladesh. Glob Health Res Policy. 2023 Aug 22;8(1):1–11.

184. Tavolacci MP, Karmaly A, El Gharbi-Hamza N, Veber B, Ladner J. Gender-based violence among healthcare students: Prevalence, description and associated factors. PloS One. 2023;18(11):e0288855.

185. Veronesi G, Ferrario MM, Giusti EM, Borchini R, Cimmino L, Ghelli M, et al. Systematic Violence Monitoring to Reduce Underreporting and to Better Inform Workplace Violence Prevention Among Health Care Workers: Before-and-After Prospective Study. JMIR Public Health Surveill. 2023;9(101669345):e47377.

186. Vu LG, Nguyen Hoang L, Le Vu Ngoc M, Nguyen Si Anh H, Nathan N, Trong Dam VA, et al. Professional Preparedness Implications of Workplace Violence against Medical Students in Hospitals: A Cross-Sectional Study. Inq 00469580. 2023 Jun 15;1–9.

187. Yan S, Feng J, Gan Y, Wang R, Song X, Luo Z, et al. Prevalence and predictors of workplace violence against emergency physicians in China: a cross-sectional study. Hum Resour Health. 2023;21(1):8.

188. Jaradat Y, Nielsen MB, Bast-Pettersen R. Psychosomatic symptoms among Palestinian nurses exposed to workplace aggression. Am J Ind Med. 2018;61(6):533–7.

189. Li JH, Chen TW, Lee HF, Shih WM. The Effects of Emergency Room Violence toward Nurse’s Intention to Leave-Resilience as a Mediator. Healthc Basel Switz. 2021;9(507).

190. Zachariadou T, Zannetos S, Chira SE, Gregoriou S, Pavlakis A. Prevalence and Forms of Workplace Bullying Among Health-care Professionals in Cyprus: Greek Version of “Leymann Inventory of Psychological Terror” Instrument. Saf Health Work. 2018;9(3):339–46.

191. Choiniere JA, MacDonnell JA, Campbell AL, Smele S. Conceptualizing structural violence in the context of mental health nursing. Nurs Inq. 2014;21(1):39–50.

192. Jafree SR. Workplace violence against women nurses working in two public sector hospitals of Lahore, Pakistan. Nurs Outlook. 2017;65(4):420–7.

193. Beattie J, Griffiths D, Innes K, Morphet J. Workplace violence perpetrated by clients of health care: A need for safety and trauma-informed care. J Clin Nurs. 2019;28(1–2):116–24.

194. Jenner SC, Djermester P, Oertelt-Prigione S. Prevention Strategies for Sexual Harassment in Academic Medicine: A Qualitative Study. J Interpers Violence. 2020;886260520903130.

195. Rossi MF, Beccia F, Cittadini F, Amantea C, Aulino G, Santoro PE, et al. Workplace violence against healthcare workers: an umbrella review of systematic reviews and meta-analyses. Public Health. 2023 Aug 1;221:50–9.

196. Siller H, Beck-Rabanser C, Hochleitner PDM, Exenberger DS. “Not a Woman-Question, But a Power-Question”: A Qualitative Study of Third Parties on Psychological Violence in Academic Medicine. Workplace Health Saf. 2021;69(1):41–9.

197. Mathews E, Hammarlund R, Kullar R, Mulligan L, Le T, Lauve S, et al. Sexual Harassment in the House of Medicine and Correlations to Burnout: A Cross-Sectional Survey. Ochsner J. 2019;19(4):329–39.

198. Lamesoo K. “Some Things Are Just More Permissible for Men”: Estonian Nurses’ Interpretations of Sexual Harassment. NORA Nord J Womens Stud. 2013;21(2):123–39.

199. Fricke J, Siddique SM, Douma C, Ladak A, Burchill CN, Greysen R, et al. Workplace Violence in Healthcare Settings: A Scoping Review of Guidelines and Systematic Reviews. Trauma Violence Abuse. 2022 Nov 6;15248380221126476.

200. Lee SK, Song JE, Kim S. Experience and perception of sexual harassment during the clinical practice of korean nursing students. Asian Nurs Res. 2011;5(3):170–6.

List of Legends

Legend	Description
BUH	Bullying, Undermining Behaviour, and Harassment
DBSH	Discrimination, Bullying, Sexual harassment, and Harassment
ED	Emergency Department
EM	Emergency Medicine
EMS	Emergency Medical services
GBDB	Gender-based Discrimination and Bias
GB-WPV	Gender-based Workplace Violence
HRH	Human Resources for Health
HV	Horizontal Violence
HWE	Hostile Work Environment
ICN	International Council of Nurses
ICU	Intensive Care Unit
IHD	Intimidation, Harassment and discrimination
ILO	International Labour Office
IMG	International Medical Graduates
JBI	Joanna Briggs Institute
LGBTQ	Lesbian, Gay, Bisexual, and Transgender
LH	Lateral Hostilities
NHS	National Health System
OBGYN	Obstetrics and Gynecology
PHC	Primary Health Care
PRISMA	Preferred Reporting Items for Systematic Reviews and Meat-Analysis
PSI	Public Services International
WHO	World Health Organization
WPV	Workplace Violence

Figure
Click here to access/download;Figure;Figure 1 PRISMA Flow Diagram.doc

Figure 1: PRISMA Flow Diagram for Screening and Selection

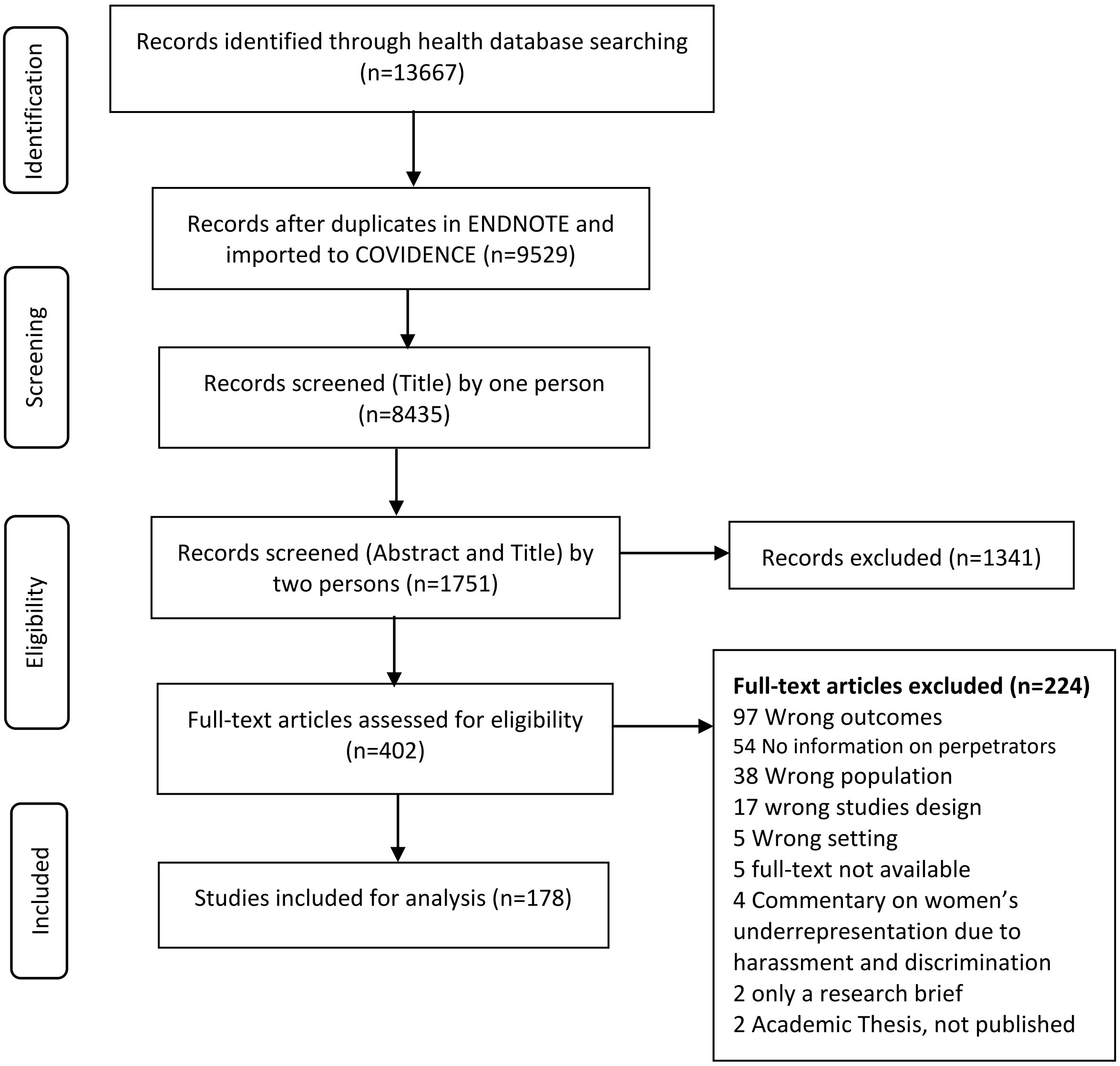

Click here to access/download
Supporting Information
S1 Appendix A- Revised Search Stretegy Ovid Revid MEDLINE .pdf

Click here to access/download
Supporting Information
S2 Appendix B-Definitions of forms of WPV.docx

Click here to access/download
Supporting Information
S3 PRISMA-ScR-filled-Checklist.pdf

Click here to access/download
Supporting Information
S4 Registered Protocol-WPV Protocal among Health workforce.pdf
