# Supplementary material for: Perpetrators of gender-based workplace violence amongst nurses and physicians–A scoping review of the literature
Source: PLOS Glob Public Health. 2024 Sep 6;4(9):e0003646. doi: 10.1371/journal.pgph.0003646 (PMC11379169; doi:10.1371/journal.pgph.0003646)
Supplement: Attachment — Submitted filename: Response to reviewers comments.docx [file pgph.0003646.s008.docx]

July 30, 2024

Dear Editors and Reviewers,

Thank you for reviewing our manuscript, "Perpetrators of Gender-based Workplace Violence Amongst Nurses and Physicians –A Scoping Review of the Literature." We sincerely thank the reviewers for their helpful suggestions that helped improve our manuscript. We have resubmitted the manuscript with changes highlighted in Yellow. We have also included our responses to the reviewers' comments below in Green.

**Reviewers' comments:**

**Reviewer #1:** Workplace violence among healthcare workers is an important area of investigation. I am glad this paper attempts to synthesise findings from other studies to highlight the perpetrator's characteristics for committing Type II and Type III GB WPV among nurses and physicians. However, there are a few issues that remain unclear. I want the author to clarify those issues as follows:

**Authors' Response:** Thank you for the review and insightful feedback. We have addressed the issues highlighted (in Yellow) in the manuscript and responses (in Green) with each comment in the following section.

***1. What is GB WPV?*** How does one identify if a particular workplace violence is gendered or non-gendered? I think a clear conceptual definition and some ideas of indicators of what constitutes gender-based workplace violence will help the reader understand the nuances of the undercurrents of workplace violence.

**Authors Response:** We capitalized on the definition by the International Labour Organization (2019) definition of "gender-based violence and harassment" means violence and harassment directed at persons because of their sex or gender, or affecting persons of a particular sex or gender disproportionately". Please see the introduction section in the manuscript.

2. Gender is defined in the paper in binary terms mainly because there are very few studies on workplace violence among the non-binary population. I would, however, suggest keeping the non-binary definition of gender and highlighting what those few studies have brought out and explaining why there are only a few studies on workplace violence among non-binary healthcare workers. This finding is in itself important from a gendered violence perspective.

**Authors Response:** First of all, thank you very much for this insightful feedback. The studies included in this review did not focus on GBV. We included studies that either reported on sex-segregated findings and/or specified gender as a factor for workplace violence or a predictor for the consequences of violence. A few studies included either non-binary (less than 4%) (DiFiori et al., 2023; Hu et al., 2022) individuals or mentioned as others (less than 4%) (Ceppa et al., 2020; Chen et al., 2021) or unknown (less than 9%) (Abrams & Robinson, 2011; Rowe et al., 2022), in the analysis of the total population in Table 2. Even those studies did not provide findings for those minority populations or address it as a limitation. Therefore, we reported findings based on gender binary. We have addressed this issue in the discussion section.

3. The paper mainly highlights the quantitative numbers of the perpetrators of workplace violence. There is no clear analysis of who these perpetrators are besides their gender. Why did they commit workplace violence? How did the original study classify that violence- gendered or non-gendered? Did the select studies describe the nature, intensity and frequency of violence? What are the other explanatory variables besides gender that the studies included that could help identify contextual responses?

**Authors Response:** Based on our preliminary search, and to accommodate the heterogeneous (operational definitions by most literature) and complex nature of the literature on violence, we set our objectives mainly at the descriptive level and summarized the perpetrator based on the gender binary, which we rationalized in the earlier comment. Keeping in mind the objectives, we landed on a scoping review methodology, which is the appropriate method to explore such a complex topic from a global perspective. Regarding other variables, such as race/ethnicity/nationality, age, years of experience, practice setting, etc., as risk factors are reported in an earlier publication on a gender-based review of workplace violence amongst the global health workforce, accessible at <https://journals.plos.org/globalpublichealth/article?id=10.1371/journal.pgph.0003336>.

This is the second paper using the same protocol. The earlier paper served as the impetus for summarizing the perpetrators based on the gender binary. Most papers included in this review provided frequency for various forms of workplace violence, but very few (34) studies provided numerical data for perpetrators for those forms of violence. In the earlier paper, we reported on 226 studies. The findings of this scoping review could provide the state of existing knowledge to recommend direction for future empirical research studies. To address this comment, we emphasized this gap and future research in the manuscript.

4. This paper does not dwell on the measurement of GB WPV. It is important first to do a critical analysis of how and in how many different ways GB WPV is being measured or described by different researchers. Is it 'ever' or 'last time', or do studies use a reference period -- last six months, or one year or so? How does one ensure that measurements across different studies are comparable?

**Authors' Response:** As we responded to an earlier comment that all the included studies did not focus on GB WPV. We included studies that either reported on sex-segregated findings and/or specified gender as a factor for workplace violence or a predictor for consequences of violence to respond to the broad question of describing the types and nature of WPV perpetrators. Some studies defined the period as six or 12 months, while others were silent. So, we defined it in the protocol to include all studies in which participants experienced WPV during their careers (please see box 1, the selection criteria in the manuscript).

5. Could GB WPV also vary according to the level of health settings? For example, nurses and physicians in primary healthcare settings could experience different kinds of pressure and expectations from clients than those working in secondary and tertiary healthcare settings. Classifying the studies according to the health settings will help us understand contextual factors that determine the GB WPV.

**Authors' Response:** The studies reported on various forms of WPV in various settings, which we presented as one of the factors for WPV in the earlier paper, now accessible from <https://journals.plos.org/globalpublichealth/article?id=10.1371/journal.pgph.0003336>.

6. Why has this review excluded other systematic/scoping reviews? The other reviews could provide useful baseline information against which this review could compare its results.

**Authors' Response:** We reviewed several systematic and scoping reviews before preparing the protocol for this review. We could not find a comprehensive review, which aimed on all forms of violence in the global context and the population we aimed at. Given the complexity of the topic and variabilities in those reviews and manageability for us, we excluded those reviews and gray literature.

**Reviewer #2: Dear Authors:**

Thank you for contributing to this growing body of work highlighting workplace related issues that often predominate, but do not receive enough attention.

This is an interesting work but does need additional attention substantiating many of the claims being made throughout, especially with regard to gender norms. I have made line by line comments in the attached version of the document. I question the helpfulness of using the workplace violence categorization given that not all of the types were used and there was a predominant focus on harassment specifically in the discussion.

Would be helpful to address how this sector relates to others with regard to workplace violence. Additionally, a limitations section, gaps to address, and recommendations section is needed.

**Author's Response:** Thank you very much for your kind feedback and the detailed line-by-line comments in the manuscript. We have copied all the comments from the manuscript with line numbers and have addressed them in the manuscript, highlighted in Yellow, and responses to comments in the following section in Green.

***Pg 8- line 88 in the table:*** where does violence perpetuated by the workers on clients come in? Would be interesting to see what proportion of perpetrators that are committing horizontal and vertical violence are also harming those within the community as well. These trends are well documented with cases of SEAH

**Authors Response:**

We believe that sexual exploitation, abuse and sexual harassment (SEAH) and gender-based violence (GBV) are closely related. However, regarding the SEAH by workers on patients/ consumers is a different area of work, and it could be interesting to explore as another review. However, the concept of SEAH linked with Structural/hierarchical inequalities within organizations, e.g., between senior and junior staff, has been catered by Type III violence, as this paper focused on Type II and III violence, which have severe effects on the health workforce and impact on patient care, healthcare organizations and system.

***Pg 9-111- women’s preponderance in the health workforce-*** is this really a factor? women who are the minority in some STEM fields like engineering also experience a high level of harassment.

**Authors Response:** Women are at higher risk than men for workplace violence in all sectors. However, there is evidence that the health sector is at a higher risk of workplace violence because of several factors, including a stressful environment, dealing with vulnerable populations, public accessibility, etc. In the discussion section, we have made some comparisons with other sectors.

***P9- 112- Societal gender norms-*** do you mean patriarchal societies or "patriarchal norms". I don't know that we should normalize these non-equitable societies with terms like "gender norms"

**Authors Response:** Patriarchal/ hierarchical societies and gendered norms

***119- gender-segregated--***is this based on a gender binary? would list this as a limitation

**Authors Response:** Yes- we used gender as binary in our research and discussed it in the discussion section.

***Table 2:Prevalence of Various Types/Forms:*** Would be helpful to create a global map showing where the majority of studies were originating from - this could show how pervasive and global this issue is and also highlight global localities that likely need to publish more on this topic

**Authors Response:** We tried mapping out countries in various organizational patterns regarding various forms of violence or gendered aspects. However, every time we looked at it, the maps misrepresented various forms of violence because of the heterogeneous nature of the studies. In addition, most of these studies were conducted in isolated parts of the countries rather than at the national level, which could also misrepresent the data. Therefore, we kept it in tabular form with the cited reference.

***In table 2, for clinical setting:*** was there any designation for and comparison of private vs public facilities?

**Authors Response:** A couple of studies specified the private/public sector; there were no significant differences. Therefore, we did not address that aspect. Instead, we focused on clinical areas/settings; some clinical settings were reported to be at a higher risk for various types of violence than others. For instance, the emergency department and psychiatric setting had a higher incidence of physical violence, and the surgical specialty had a higher prevalence of verbal violence and sexual harassment, reported in a prior publication from this review as risk factors.

***In table 3, Forms of violence:*** is there a supplemental document that defines each of these to ensure the definitions across these studies are similar? Also is there a way to make a gradation from least to most severe? Or even offenses that are punishable versus not? This last part could highlight the lack of workplace policy in place needed to protect staff.

**Authors Response:** Yes- While definitions of various forms of violence vary widely in various legal jurisdictions, contexts, and research, we have provided a supplemental document that defines these forms of violence based on the joint framework of the ILO, ICH, WHO, and PSI in 2002.

***Discussion:*** This whole section should be cut down, focused, and streamlined so that it does not repeat your results. You need to be relating your results to other global studies. Also SEVERAL statements made in tis section are not well substantiated. One citation from one country is not adequate to generalize behavioral norms seen globally.

**Authors Response:** We have modified the section. Please see in the manuscript.

***296-298- About the proportion of perpetrator (men and women):*** maybe also good to recognize the trends in the type of behavior most frequently perpetuated by gender - e.g., sexual harassment by men and bullying by women.

**Authors' Response:** The specific patterns of these behaviours are explained in the subsequent discussion sections for Type II and III violence.

311 the ***higher perpetration of WPV by men is linked to societal norms associating aggression and dominance with masculinity. At the same time, violence against a feminized nursing***

***313 workforce is normalized as part of the job (191).*** These kind of statements may not need to be made if there is a global assumption that violence in the workplace is wrong no matter who this is perpetuated against.

**Authors Response:** There were studies that explained the normalization of violent behaviours for various populations. The data provides evidence that some groups are at higher risk than others. We added evidence to explain the statement.

***314-315- These norms stem from social relations which dictate gender roles and***

***responsibilities, and healthcare institutions are no exception to these forces***. what citations do you have to substantiate this?

**Authors Response:** Added in the manuscript

***325-326- A similar pattern emerged in Saudi Arabia, with male physicians and nurses reporting a prevalence of 57.8%, while their female counterparts reported 42.8% (35).*** Could this be location specific? What proportion of physicians are male? Do the women physicians work in private facilities or serve a different clientele? Do women have the agency to report violence they experience?

**Authors Response:** Please see the added explanation for this, highlighted in Yellow in the manuscript.

***332-333- can create a vertical hierarchy where women are assigned to lower-level tasks-*** would be good to relate to/cite studies that discuss women in leadership roles globally in the field of medicine to substantiate this.

**Authors Response:** Added evidence

***334-335- On the other hand, it relates to the negative stereotypes that women are weak, indecisive and incompetent, which, in turn, sets the stage for higher rates of violence and discrimination.*** What do you have to substantiate this?

**Authors Response:** Added evidence.

337***Jafree (192) calls on policymakers to ensure security and protection for the health workforce, particularly women; legislative reforms for healthcare governance and zero-tolerance policies for violence were also recommended.***

How many of these papers discussed reporting mechanisms, retaliation, policies in place, recommended or enacted? Would be helpful to enumerate this to help identify actionable steps/recommendations for how to address workplace violence.

**Authors' Response:** Several studies explain different rates and reporting mechanisms, regardless of gender, that we did not quantify to keep our paper’s focus on the gendered aspect. We do provide some material on the legal aspects and reporting mechanisms in various contexts.

***336- affected were nurses working in psychiatric wards (195).*** How much of the violence perpetuated by patients to staff is unintended? e.g. people who are demented, etc. and don't have the ability to control violent outbursts. This would be important to note in this section ESPECIALLY if you are not going to cover abuse or violence that the health care staff could be inflicting on this patient population (e.g. thinking about withholding pain medicine or other medications (gender affirming care), violence, SEAH, etc.)

**Authors' Response:** This is an important aspect, and we have discussed it as a risk factor (clinical settings) of workplace violence in a prior publication of this comprehensive review. As mentioned in an earlier comment about SEAH by workers on patients/ consumers, it is a different area of work and requires a different set of interventions to deal with—it could be interesting to explore in another review.

***384- Type III violence is rooted in cultural norms and societal expectations that allocate***

***roles and responsibilities based on gender (61).*** do not think this citation alone is enough to substantiate this statement

**Authors' Response:** Several of our studies highlighted this aspect; we have added the references.

***389-391- women experienced significantly higher.*** would be could to compare the outcomes you are seeing here for healthcare workers to other workplace settings like the public health field, other STEM fields, etc RATHER than more general citations - because you will want to find solutions for eliminating this - what are other sectors doing to address this?

**Authors Response:** Added a couple of sources from STEM explaining differences in factors factors.

***401-403*- realistic workloads and- skill*-mixed staffing, promoting gender equality in staff allocation, and participatory leadership to overcome relational conflict and managerial actions that enhance working conditions.*** people need to be held accountable - realistic workloads is not going to solve issues around sexual harassment.

**Authors' Response:** We believe the highlighted aspects are important for transforming organizational culture and adding evidence-based data. The subsequent section provided recommendations addressing individuals’ accountability.

**425-426- This stemmed from beliefs and negative stereotypes, such as women being weak, unwilling to speak up, indecisive and incompetent (61).**

One citation is not adequate in making a generalization on why this is happening across all these studies.

**Authors Response:** We have added additional references.

***430- among women (197).*** This is a US specific study - you can't use this to make a global statement

**Authors' Response:** We modified this section by removing some aspects.

***518- The review revealed a higher prevalence of Type II and Type III WPV among women-*** reflecting on the focus around harassment in the discussion section I am wondering if this categorization is the best for your study. It may be more beneficial to look specifically at workplace harassment since several of these categories were not even addressed and this framework does not include harassment or violence perpetuated by the healthcare staff/sector to the community/population they serve.

**Authors' Response:** Given the variability in the definitions from various contexts and research in our data, we defined and operationalized the forms of violence from the seminal work of Di Martino (2002) based on the joint program of ILO, WHO, ICN, and PSI, which also included harassment as a form of violence (please also refer to Appendix A). Many of our papers, too, included harassment as a form of violence, as presented in Table 2.

***521- Only Type III-B violence, including bullying/ mobbing, was occasionally perpetuated by women.*** felt this was not adequately discussed

**Authors' Response:** We made some additions to the results and discussion.
